# Synthesis, Fungicidal Activity and SAR of 2-Thiazolamide/Pyrazolamide-Cyclohexylsulfonamides against *Botrytis cinerea*

**DOI:** 10.3390/molecules24142607

**Published:** 2019-07-17

**Authors:** Shen Zhang, Siqi Meng, Yong Xie, Yonggui Yang, Yumeng Zhang, Lu He, Kai Wang, Zhiqiu Qi, Mingshan Ji, Peiwen Qin, Xinghai Li

**Affiliations:** 1 Department of Pesticide Science, Plant Protection College, Shenyang Agricultural University, Shenyang 110866, Liaoning, China; 2 State Key Laboratory of the Discovery and Development of Novel Pesticide (Shenyang Sinochem Agrochemicals R&D Co. Ltd.), Shenyang 110866, Liaoning, China

**Keywords:** cyclohexylsulfonamides, thiazole, pyrazole, fungicidal activity, *Botrytis cinerea*

## Abstract

In order to explore more efficient sulfonamides against *Botrytis cinereal*, 36 novel cyclohexylsulfonamides were synthesized by *N*-(3-dimethylaminopropyl)-*N*′-ethylcarbodiimide (EDCI) and 1-hydroxybenzotriazole (HOBt) condensation reaction using chesulfamide as a lead compound, introducing thiazole and pyrazole active groups. Their structures were characterized by ^1^H-NMR, ^13^C-NMR, mass spectrum (MS), and elemental analysis. Compound III -31 was further confirmed by X-ray single crystal diffraction. The in vitro and in vivo fungicidal activities against *B. cinerea* were evaluated by three bioassay methods. The results of mycelial growth demonstrated that median effective concentration (EC_50_) values of nine compounds were close to boscalid (EC_50_ = 1.72 µg/mL) and procymidone (EC_50_ = 1.79 µg/mL) against *B. cinerea* (KZ-9). In the spore germination experiment, it was found that compounds III-19 and III-31 inhibited germination 93.89 and 98.00%, respectively; at 10 µg/mL, they approached boscalid (95.97%). In the tomato pot experiment, the control effects of two compounds (III-21 and III-27) were 89.80 and 87.90%, respectively, at 200 µg/mL which were significantly higher than boscalid (81.99%). The structure–activity relationship (SAR) was also discussed, which provided a valuable idea for developing new fungicides.

## 1. Introduction

*Botrytis cinerea* is one of the top 10 fungal pathogens that causes grey mould or Botrytis blight. It mainly affects vegetables and fruits, as well as a large number of flowers, shrubs, weeds and trees worldwide [1,2,3,4,5]. Many fungicides have failed to control *B. cinerea* because of their high risk for fungicide resistance development [6]. Therefore, it is worth developing and synthesizing new safe products that can effectively control *B. cinerea*.

It is a common familiarity that sulfonamide compounds have excellent biological activities not only in the field of pharmaceuticals [7,8] but also as agrochemicals [9,10,11,12], such as herbicides and fungicides. A novel sulfonamide fungicide chesulfamide [13,14] is different from other commercial fungicides; it showed excellent fungicidal activity against *B. cinerea* due to its unique mechanism of action, including (1) acting on mycelium cell membrane, (2) disturbing DNA, and (3) inducing disease resistance of plants [13,15]. Therefore, chesulfamide was used as a lead compound.

**Figure 1:**
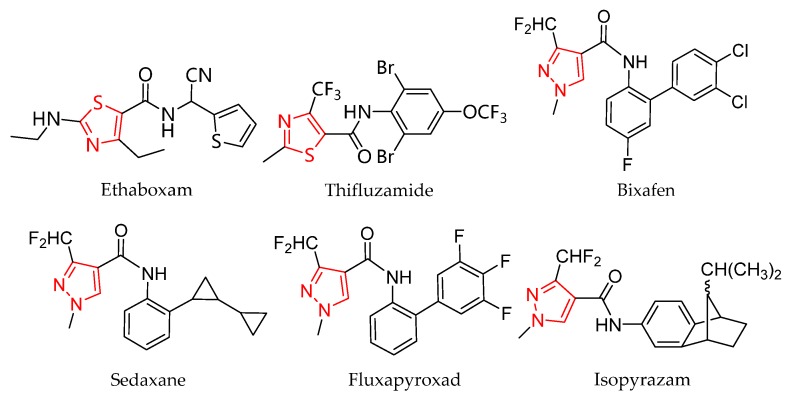
Chemical structure containing thiazole and pyrazole fungicides.

**Scheme 1:**
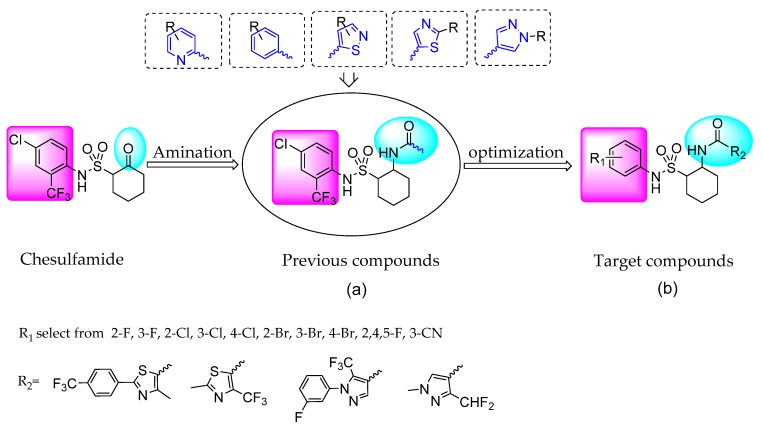
Design ideas for the target compounds.

In the current situation, thiazolamide and pyrazolamide fungicides have become the dominant fungicide varieties, such as Ethaboxam, Thfluzamide [16], Bixafen [17], Sedaxane [18], Fluxapyroxad [19], and Isopyrazam [20] (Figure 1). Among them, the alkyl-thiazole/pyrazole group was selected. In the previous study, our laboratory placed emphasis on the study of chesulfamide and synthesized a series of 2-pyridyl, 2-phenyl 2-isothiazolyl, 2-thiazolyl and 2-pyrazolyl cyclohexylsulfonamides against *B. cinerea* [21,22,23,24,25] (Scheme 1a). Phenylthiazole and phenylpyrazole active substructures were screened out. Hence, the fungicidal activities of two substituted thiazoles/pyrazoles compounds were contrasted.

**Scheme 2:**
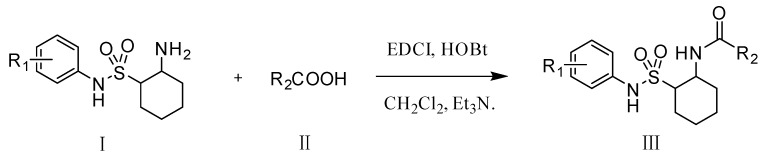
Synthetic route of the target compounds III.

In this study, we used chesulfamide as a lead compound, reducing the carbonyl group to an amino group [26,27], and the EDCI/HOBt condensation method [24,25], interfacing with the pyrazole carboxylic acid and thiazole carboxylic acid. In order to improve the fungicidal activities against *B. cinerea*, the benzene ring (linked to NH) was optimized, which was different from the structure of 2-trifluoromethyl-4-chlorobenzene before. Moreover, 18 novel 2-thiazolamide cyclohexylsulfonamides and 18 novel 2-pyrazolamide cyclohexylsulfonamides were designed and synthesized (Scheme 1b). The in vivo and in vitro fungicidal activities against *B. cinerea* were evaluated and the structure–activity relationship was analyzed. The synthetic route of the target compounds was shown in Scheme 2.

## 2. Results

### 2.1. Chemistry

The corresponding aminocyclohexylsulfonamide (I), HOBt and EDCI in the presence of triethylamine (Et_3_N) in dichloromethane (CH_2_Cl_2_) as solvent were allowed to react with different carboxylic acids (II) at 0 °C. Finally, 36 novel cyclohexylsulfonamides compounds III were synthesized.

**Figure 2:**
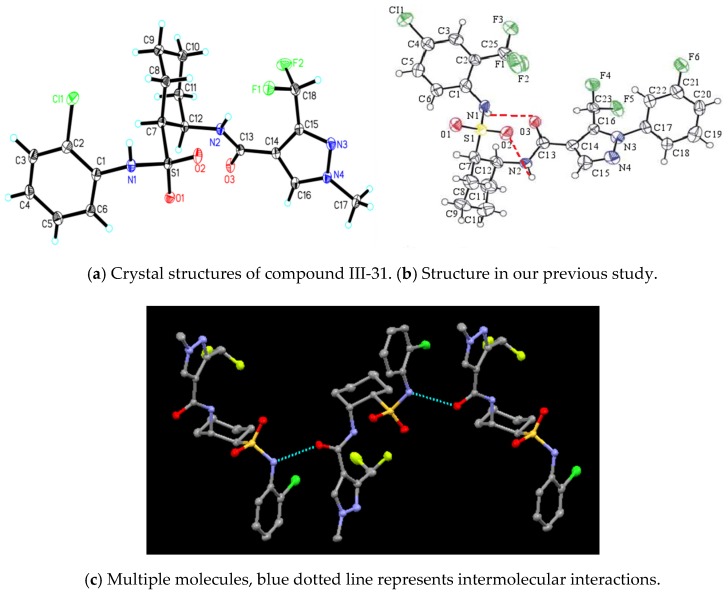
X-ray crystal structures.

The structure of compound III-31 was analyzed by X-ray single crystal diffraction. The structure (CCDC: 1917351) was shown in Figure 2a and available in Supplementary Data. X-ray diffraction provided a relative configuration of chiral centers, hence it proved the *cis*-configuration at the cyclohexane ring. Probably, the compound III-31 was racemates, which mixed both enantiomers (“C_7_”R, “C_12_”S and “C_7_”S, “C_12_”R). A compound reported (Figure 2b) [26] with a similar structure showed that there were two intramolecular hydrogen bonds in the structure, N_1_-H^…^O_3_ and N_2_-H^…^O_2_. On the contrary, in our study, compound III-31 had no intramolecular hydrogen bonds, but had intermolecular hydrogen bonds, with N_1_ in one molecule being linked to O_3_ in another molecule, eventually forming the chain structure (Figure 2c).

**Figure 3:**
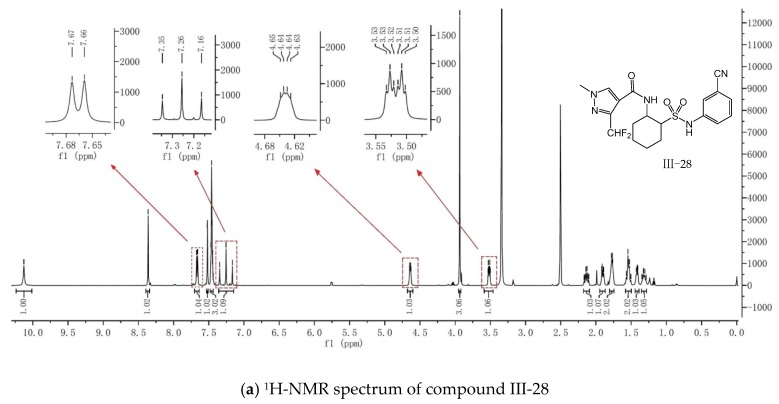

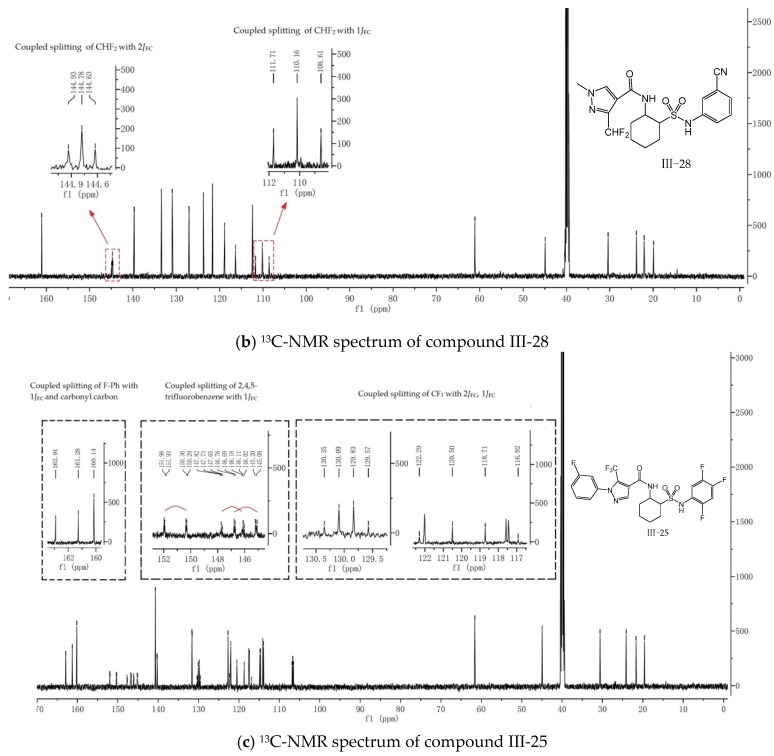
Structure elucidation of compounds III.

Additionally, in this work, the chemical structures of all the synthesized compounds were characterized by nuclear magnetic resonance (NMR), mass spectrum (MS) and elemental analysis. The ^1^H NMR and ^13^C NMR spectra are available in Supplementary Data. In the ^1^H NMR spectra (600 MHz, DMSO-*d*_6_) of compound III-28 (Figure 3a), the protons on the benzene ring were revealed in a low field in the range of *δ*_H_ 7.4 to 7.6 ppm, while cyclohexyl and methyl group gave signals in the range of *δ*_H_ 1.0 to 5.0 ppm. However, pyrazole-H and SO_2_NH appeared at *δ*_H_ 8.36 ppm and 10.13 ppm, respectively. The carboxamide proton of NH–C=O showed doublet (d) at *δ*_H_ 7.67 ppm, *J* = 8.4 Hz. The signal of proton of CH-SO_2_ at *δ*_H_ 4.64 ppm revealed only coupling constants less than 9 Hz, indicating the equatorial position of this proton. Analogously, the axial orientation of the proton on CH-N was revealed by its splitting to doublet of triplets (dt) at *δ*_H_ 3.52 ppm with one diaxial coupling of *J* = 11.4 Hz and two axial equatorial couplings of *J* = 3.6 Hz. The splitting pattern of protons on CHF_2_ is very characteristic. Generally, the proton of CHF_2_ showed triplet (t) at *δ*_H_ 7.26ppm, *J* = 54 Hz.

In the ^13^C-NMR spectra (151 MHz, DMSO-*d*_6_) of compound III-28 (Figure 3b), the signals in the range of *δ*_C_ 0 to 70 ppm were assigned to methyl and cyclohexyl groups. Benzene ring carbons and most of the pyrazole carbons resonated in the range of *δ*_C_ 112 to 140 ppm and the signal at *δ*_C_ 161 ppm belonged to C=O. The difluoromethyl group caused the triplet at *δ*_C_ 110.16 ppm with ^1^*J*_FC_ = 234.05 Hz, while the adjacent carbon signal at *δ*_C_ 144.78 ppm is split into a triplet due to ^2^*J*_FC_ = 22.80 Hz.

In the ^13^C-NMR spectra (151 MHz, DMSO-*d*_6_) of compound III-25 (Figure 3c), there were very interesting phenomena due to seven fluorine atoms. Carbon signals of C-F in the F-Ph showed a doublet at *δ*_C_ 162.10 ppm, ^1^*J*_FC_ = 246.43 Hz, while carbon signals of C-F in 2,4,5-trifluorobenzene, which appeared in the range of *δ*_C_ 145 to 151 ppm, showed ^1^*J*_FC_ couplings of 245.38, 244.77, 239.03 Hz, respectively, and further long-range C-F coupling. The carbon signals of CF_3_ appeared as a quartet at *δ*_C_ 119.61 ppm with ^1^*J*_FC_ = 270.74 Hz, while the quartet of the adjacent carbon at *δ*_C_ 129.96 ppm showed the ^2^*J*_FC_ coupling of 39.26 Hz. 

### 2.2. Biological Assay and Structure—Activity Relationship Study

In order to screen out active compounds quickly and correctly, the target compounds were tested by an in vitro mycelium growth inhibition assay and the spore germination method. Then, active compounds were tested in tomato pot experiments. 

#### 2.2.1. In Vitro Fungicidal Activity against *B. cinerea*

The germicidal virulence test of 36 target compounds was tested against the *B. cinerea* strain (KZ-9), with boscalid and procymidone as standard fungicides. The EC_50_ values of 17 compounds were lower than 10 μg/mL. Compounds III-19 and III-27 showed better in vitro fungicidal activity against KZ-9, with the EC_50_ values of 1.99 and 2.04 μg/mL (Table 1). Some compounds showed excellent fungicidal activity against KZ-9 and were rescreened out by another *B. cinerea* strain (CY-09). The EC_50_ value of compound III-21 against CY-09 was 2.33 μg/mL, which showed its excellent fungicidal activity (Table 2).

The results represented in Table 3 indicated that several compounds can inhibit the spore germination of *B. cinerea* at the concentration of 10 μg/mL. Among these, compounds III-19 and III-31 showed superb activity on spore germination, which had higher inhibition rates (93.89 and 98.0%) were comparable to boscalid (95.97%).

#### 2.2.2. In Vivo Fungicidal Activity against *B. cinerea*

After that, the in vivo control effect of active compounds against *B. cinerea* on tomato leaves were tested at 200 μg/mL. The results showed that the control effects of compounds III-19, III-21 and III-27 were 78.00, 89.80 and 87.90%, respectively, which were similar to boscalid (81.99%). The three compounds showed excellent activities in in vivo tests (Table 4).

Accordingly, by analyzing the experimental results, the structure–activity relationship (SAR) was summarized as follows: (1) Compounds III-10–III-18 had the same alkyl-thiazole structure as that of Thifluzamide while compounds III-1–III-9 had phenylthiazole structure. It was noted that compounds III-1–III-9 showed significantly improved fungicidal activities. In addition, compounds containing pyrazoles revealed the same results; it showed that fungicidal activities of compounds III-19–III-27 were better than compounds III-28–III-36. In total, compounds with benzene ring-substituted thiazole or pyrazole active groups showed more highly fungicidal activities. (2) For 2-thiazolamide-cyclohexylsulfonamides (III-1–III-18), the activity was significantly increased when the benzene ring (linked to NH) contained fluorine-containing groups (2-F, 3-F and 2,4,5-F), such as compounds III-1, III-2, III-9 and III-11. When the benzene ring (linked to NH) contained chlorine, the phenylthiazole compounds III-3 and III-4 showed excellent fungicidal activity. However, fungicidal activity was mediocre on the benzene ring containing bromine. (3) For 2-pyrazolamide-cyclohexylsulfonamides (III-19–III-36), the activity was higher when the benzene ring (linked to NH) contained fluorine-containing groups (2-F, 3-F), such as phenylpyrazole compounds III-19 and III-27, but, if the substituent was a 2,4,5-fluorine, the fungicidal activity slightly decreased. Additionally, when the benzene ring (linked to NH) contained 4-chlorine, the compounds III-21 and III-36 showed excellent activity. When the benzene ring (linked to NH) contained bromine, the phenylpyrazole compounds III-23 and III-24 showed excellent activity. 4) It could be seen that the compounds III containing pyrazole substituent showed higher fungicidal activity than thiazole substituent.

## 3. Materials and Methods

### 3.1. Materials and Instrumentation

The reagents and solvents were commercially available for analytical reagent (AR) grade and were used as received or were dried prior to use, as needed. The melting point was measured by X-5 binocular microscope melting point apparatus (Beijing Tech Instruments Co. Ltd., Beijing, China). Using dimethyl sulfoxide (DMSO-*d*_6_) as solvent and tetramethylsilane (TMS) as the internal standard, ^1^H-NMR spectra were recorded on 300 MHz and 600 MHz spectrometers and ^13^C-NMR spectra were recorded on 75 MHz and 151 MHz spectrometers (Bruker, Karlsruhe, Germany). MS data were obtained on the 7000C Triple Quad GC/MS and 6460 Triple Quad LC/MS Mass Spectrometers (Agilent Technologies, Santa Clara, CA, USA). Elemental analyses were determined on a Vario EL III elemental analyser (Elementar Analysensysteme GmbH, Frankfurt, Germany). 

### 3.2. Synthesis

#### 3.2.1. Synthesis of *N*-Substituted Phenyl-2-Aminocyclohexylsulfonamides I

The synthesis of *N*-substituted phenyl-2-aminocyclohexylsulfonamide I was done according to the reported method [10,21,27]. In this paper, 2-trifluoromethyl-4-chloroaniline in the reference was replaced by different substituted aniline, but the experimental methods were consistent. The yield of the compounds I was 40–75%.

#### 3.2.2. Synthesis of Target Compounds III

Under nitrogen atmosphere, carboxylic acid II (3mmol), EDCI (3.3 mmol), HOBT (3.3 mmol) and Et_3_N (1.8 mmol) were placed in a three-necked flask with 40 mL CH_2_Cl_2_, and stirred for 2 h at 0 °C; then, compound I (2.4 mmol) was added to the flask and allowed to react for 3 h at 0 °C. The reaction was monitored by thin-layer chromatography (TLC) (all reactions could be completed in 3 h) and, on completion of the reaction, the mixture was washed with saturated NaHCO_3_ solution and water, respectively. Then, it was dried over anhydrous Na_2_SO_4_, filtered and evaporated on rotavapor in vacuum. Subsequently, crude products III-1–III-18 were purified by silica gel column chromatography [V (CH_2_Cl_2_): V (EA) = 3:1] and crude products III-19–III-36 were purified by silica gel column chromatography [V (PE): V (EA) = 3:1]. Finally, products were recrystallized with the dichloromethane/petroleum ether to obtain pure target compounds.

*N-(2-fluorophenyl)-2-[4-methyl-2-(4-trifluoromethylphenyl)-5-thiazolecarboxylamino]-cyclohexylsulfonamide* III-1. White solid, yield: 85%. mp 156.8~157.7 °C. ^1^H-NMR (300 MHz, DMSO-*d_6_*): δ (ppm) 9.63 (s, 1H, NH-SO_2_), 8.22–7.17 (m, 9H, NH–C=O + C_6_H_4_ + C_6_H_4_), 4.68 (dd, *J =* 7.5, 3.3 Hz, 1H, CH–SO_2_), 3.33–3.30 (m, 1H, CH–N) (overlap with water peak), 2.63 (s, 3H, CH_3_), 2.16–1.27 (m, 8H, 4CH_2_). ^13^C-NMR (75 MHz, DMSO-*d*_6_): δ (ppm) 162.86 (d, ^1^*J*_FC_ = 237.0 Hz, F-Ph), 157.33, 154.92, 154.08, 141.65, 136.46, 130.83 (d, ^2^*J*_FC_ = 32.25 Hz), 128.81, 127.43, 127.37, 126.86, 126.81, 126.77, 125.28, 125.17, 116.63, 116.37, 60.21, 45.96, 30.41, 24.17, 22.07, 19.89, 17.31. Electron impact mass spectrometry (EIMS), *m/z* 541.58 (M). Elemental analysis for C_24_H_23_F_4_N_3_O_3_S_2_: found C 53.23, H 4.28, N 7.76; calcd C 53.02, H 4.12, N 7.98.

*N-(3-fluorophenyl)-2-[4-methyl-2-(4-trifluoromethylphenyl)-5-thiazolecarboxylamino]-cyclohexylsulfonamide* III-2. White solid, yield: 82%. mp 160.6~161.7 °C. ^1^H-NMR (300 MHz, DMSO-*d_6_*): δ (ppm) 10.06 (s, 1H, NH-SO_2_), 8.21-6.87 (m, 9H, NH–C=O + C_6_H_4_ + C_6_H_4_), 4.65 (dd, *J =* 6.9, 3.6 Hz, 1H, CH–SO_2_), 3.46 (dt, *J =* 11.1, 3.3 Hz, 1H, CH–N), 2.64 (s, 3H, CH_3_), 2.15–1.23 (m, 8H, 4CH_2_). ^13^C-NMR (151 MHz, DMSO-*d*_6_): δ (ppm) 164.41, 162.91 (d, ^1^*J*_FC_ = 243.11 Hz, F-Ph), 161.11, 155.04, 140.56 (d, ^3^*J*_FC_ = 10.57 Hz), 136.48, 131.51 (d, ^3^*J*_FC_ = 10.57 Hz), 130.84 (q, ^2^*J*_FC_ = 31.71 Hz), 128.71, 127.37, 126.76, 124.40 (q, ^1^*J*_FC_ = 273.31 Hz, CF_3_), 115.19, 110.56, 110.42, 106.26, 106.10, 60.39, 46.14, 30.26, 23.86, 22.26, 20.04, 17.33. EIMS, *m/z* 541.58 (M). Elemental analysis for C_24_H_23_F_4_N_3_O_3_S_2_: found C 53.23, H 4.28, N 7.76; calcd C 53.51, H 4.07, N 7.88.

*N-(2-chlorophenyl)-2-[4-methyl-2-(4-trifluoromethylphenyl)-5-thiazolecarboxylamino]-cyclohexylsulfonamide* III-3. White solid, yield: 84%. mp 117.0~118.4 °C. ^1^H-NMR (300 MHz, DMSO-*d_6_*): δ (ppm) 9.46 (s, 1H, NH-SO_2_), 8.19–7.21 (m, 9H, NH–C=O + C_6_H_4_ + C_6_H_4_), 4.71 (dd, *J =* 7.2, 3.0 Hz, 1H, CH–SO_2_), 3.41–3.37 (m, 1H, CH–N) (overlap with water peak), 2.63 (s, 3H, CH_3_), 2.09–1.23 (m, 8H, 4CH_2_). ^13^C-NMR (75 MHz, DMSO-*d*_6_): δ (ppm) 164.44, 161.36, 154.90, 136.46, 134.56, 131.03, 130.61, 130.32, 128.81, 128.70, 128.33, 128.24, 127.53, 127.38, 126.82, 126.76, 126.70, 62.37, 45.93, 30.52, 24.30, 22.16, 19.84, 17.31. EIMS, *m/z* 558.03 (M). Elemental analysis for C_24_H_23_ClF_3_N_3_O_3_S_2_: found C 51.66, H 4.15, N 7.53; calcd C 51.78, H 4.02, N 7.66.

*N-(3-chlorophenyl)-2-[4-methyl-2-(4-trifluoromethylphenyl)-5-thiazolecarboxylamino]-cyclohexylsulfonamide* III-4. White solid, yield: 85%. mp 116.5~117.8 °C. ^1^H-NMR (300 MHz, DMSO-*d_6_*): δ (ppm) 10.12 (s, 1H, NH-SO_2_), 8.31–7.36 (m, 9H, NH–C=O + C_6_H_4_ + C_6_H_4_), 4.71 (dd, *J* = 7.2, 3.0 Hz, 1H, CH–SO_2_), 3.54–3.45 (m, 1H, CH–N) (overlap with water peak), 2.61 (d, *J* = 9.0 Hz, 3H, CH_3_), 2.13–1.31 (m, 8H, 4CH_2_). ^13^C-NMR (75 MHz, DMSO-*d*_6_): δ(ppm) 164.40, 161.15, 154.96, 136.45, 134.65, 134.30, 129.75, 128.72, 127.37, 127.12, 126.93, 126.76, 122.59, 117.29, 109.99, 109.34, 109.14, 62.21, 45.93, 30.50, 24.35, 22.18, 19.83, 17.31. EIMS, *m/z* 558.03 (M). Elemental analysis for C_24_H_23_ClF_3_N_3_O_3_S_2_: found C 51.66, H 4.15, N 7.53; calcd C 51.79, H 4.01, N 7.62.

*N-(4-chlorophenyl)-2-[4-methyl-2-(4-trifluoromethylphenyl)-5-thiazolecarboxylamino]-cyclohexylsulfonamide* III-5. White solid, yield: 86%. mp 198.0~198.9 °C. ^1^H-NMR (300 MHz, DMSO-*d_6_*): δ (ppm) 9.93 (s, 1H, NH-SO_2_), 8.20–7.21 (m, 9H, NH–C=O + C_6_H_4_ + C_6_H_4_), 4.63 (dd, 1H, *J* = 5.4, 2.1 Hz, 1H, CH–SO_2_), 3.37 (dt, *J* = 11.4, 3.3 Hz, 1H, CH–N), 2.63 (s, 3H, CH_3_), 2.08–1.24 (m, 8H, 4CH_2_). ^13^C-NMR (151 MHz, DMSO-*d*_6_): δ (ppm) 164.52, 161.16, 155.05, 137.57, 136.43, 130.86 (q, ^2^*J*_FC_ = 33.22 Hz), 129.70 (two carbon atoms), 128.67, 128.23, 127.68, 127.38, 126.79 (d, ^3^*J*_FC_ = 4.53 Hz), 124.38 (q, ^1^*J*_FC_ = 273.31 Hz, CF_3_) 121.48 (two carbon atoms), 120.79, 60.30, 46.10, 30.27, 23.88, 22.22, 19.97, 17.31. EIMS, *m/z* 558.03 (M). Elemental analysis for C_24_H_23_ClF_3_N_3_O_3_S_2_: found C 51.66, H 4.15, N 7.53; calcd C 51.52, H 3.99, N 7.81.

*N-(2-bromophenyl)-2-[4-methyl-2-(4-trifluoromethylphenyl)-5-thiazolecarboxylamino]-cyclohexylsulfonamide* III-6. White solid, yield: 83%. mp 112.3~113.3 °C. ^1^H-NMR (300 MHz, DMSO-*d_6_*): δ (ppm) 9.35 (s, 1H, NH-SO_2_), 8.19–7.14 (m, 9H, NH–C=O + C_6_H_4_ + C_6_H_4_), 4.72 (dd, *J* = 7.2, 2.7 Hz, 1H, CH–SO_2_), 3.44–3.39 (m, 1H, CH–N) (overlap with water peak), 2.63 (s, 3H, CH_3_), 2.10–1.39 (m, 8H, 4CH_2_). ^13^C-NMR (151 MHz, DMSO-*d*_6_): δ (ppm) 164.44, 161.34, 154.91, 136.46, 135.94, 133.56, 130.84 (q, ^2^*J*_FC_ = 31.71 Hz), 130.10, 128.90, 128.82, 128.02, 127.95, 127.38, 126.78, 124.40 (q, ^1^*J*_FC_ = 273.31 Hz, CF_3_), 119.41, 99.99, 62.66, 45.95, 30.56, 24.32, 22.19, 19.82, 17.32. EIMS, *m/z* 602.49 (M). Elemental analysis for C_24_H_23_BrF_3_N_3_O_3_S_2_: found C 47.85, H 3.85, N 6.97; calcd C 47.62, H 4.01, N 7.11.

*N-(3-bromophenyl)-2-[4-methyl-2-(4-trifluoromethylphenyl)-5-thiazolecarboxylamino]-cyclohexylsulfonamide* III-7. White solid, yield: 84%. mp 187.2~188.3 °C. ^1^H-NMR (300 MHz, DMSO-*d_6_*): δ (ppm) 10.02 (s, 1H, NH-SO_2_), 8.19–7.22 (m, 9H, NH–C=O + C_6_H_4_ + C_6_H_4_), 4.65 (dd, *J* = 5.7, 2.4 Hz, 1H, CH–SO_2_), 3.45 (dt, *J* = 11.1, 3.6 Hz, 1H, CH–N), 2.64 (s, 3H, CH_3_), 2.14–1.28 (m, 8H, 4CH_2_). ^13^C-NMR (151 MHz, DMSO-*d_6_*): δ (ppm) 164.41, 161.10, 155.08, 140.40, 139.85, 136.49, 134.55, 131.75, 130.84 (q, ^2^*J*_FC_ = 31.71 Hz), 128.67, 127.37, 126.81, 126.68, 124.40 (q, ^1^*J*_FC_ = 271.8 Hz, CF_3_), 122.50, 121.77, 118.24, 60.59, 46.16, 30.25, 23.84, 22.30, 20.06, 17.36. EIMS, *m/z* 602.49 (M). Elemental analysis for C_24_H_23_BrF_3_N_3_O_3_S_2_: found C 47.85, H 3.85, N 6.97; calcd C 48.02, H 3.66, N 7.12.

*N-(4-bromophenyl)-2-[4-methyl-2-(4-trifluoromethylphenyl)-5-thiazolecarboxylamino]-cyclohexylsulfonamide* III-8. White solid, yield: 88%. mp 209.5~210.6 °C. ^1^H-NMR (300 MHz, DMSO-*d_6_*): δ (ppm) 9.95 (s, 1H, NH-SO_2_), 8.19–7.16 (m, 9H, NH–C=O + C_6_H_4_ + C_6_H_4_), 4.64 (dd, *J* = 6.9, 3.0 Hz, 1H, CH–SO_2_), 3.41–3.39 (m, 1H, CH–N) (overlap with water peak), 2.64 (s, 3H, CH_3_), 2.09–1.28 (m, 8H, 4CH_2_). ^13^C-NMR (151 MHz, DMSO-*d_6_*): δ (ppm) 164.43, 161.11, 155.01, 138.09, 136.48, 132.61 (two carbon atoms), 130.84 (q, ^2^*J*_FC_ = 31.71 Hz), 128.75, 127.38, 126.81, 126.78, 124.40 (q, ^1^*J*_FC_ = 271.8 Hz, CF_3_), 121.95, 121.69 (two carbon atoms), 116.16, 60.27, 46.08, 30.29, 23.92, 22.22, 19.98, 17.35. EIMS, *m/z* 602.49 (M). Elemental analysis for C_24_H_23_BrF_3_N_3_O_3_S_2_: found C 47.85, H 3.85, N 6.97; calcd C 47.69, H 4.00, N 7.12.

*N-(2,4,5-trifluorophenyl)-2-[4-methyl-2-(4-trifluoromethylphenyl)-5-thiazolecarboxylamino]-cyclohexylsulfonamide* III-9. White solid, yield: 86%. mp 167.0~168.0 °C. ^1^H-NMR (300 MHz, DMSO-*d_6_*): δ (ppm) 9.86 (s, 1H, NH-SO_2_), 8.19–7.47 (m, 7H, NH–C=O + C_6_H_2_ + C_6_H_4_), 4.66 (dd, *J* = 6.9, 2.1 Hz, 1H, CH–SO_2_), 3.48-3.42 (m, 1H, CH–N) (overlap with water peak), 2.62(s, 3H, CH_3_), 2.12-1.44 (m, 8H, 4CH_2_). ^13^C-NMR (151 MHz, DMSO-*d_6_*): δ (ppm) 170.78, 164.45, 161.23, 155.00, 151.23 (dd, ^1^*J*_FC_ = 243.11, 9.82 Hz, F-Ph), 147.03 (dt, *J*_FC_ = 248.24, 11.33 Hz, F-Ph), 146.07 (dd, ^1^*J*_FC_ = 245.68, 13.44 Hz, F-Ph) 136.46, 130.85 (q, ^2^*J*_FC_ = 30.2 Hz), 128.66, 127.37, 126.76, 124.39 (q, ^1^*J*_FC_ = 271.8 Hz, CF_3_),122.10, 114.87,114.83, 106.77 (dd, ^2^*J*_FC_ = 26.73, 22.20 Hz), 60.21, 45.85, 30.40, 24.15, 21.21, 17.28, 14.53. EIMS, *m/z* 577.56 (M). Elemental analysis for C_24_H_21_F_6_N_3_O_3_S_2_: found C 49.91, H 3.67, N 7.28; calcd C 50.11, H 3.82, N 7.10.

*N-(2-fluorophenyl)-2-(2-methyl-4-trifluoromethyl-5-thiazolecarboxylamino]-cyclohexylsulfonamide* III-10. White solid, yield: 88%. mp 175.6~176.8 °C. ^1^H-NMR (300 MHz, DMSO-*d_6_*): 9.52 (s, 1H, NH–SO_2_), 8.91 (d, *J* = 8.7 Hz, 1H, NH–C=O), 7.46–7.18 (m, 4H, C_6_H_4_), 4.68 (dd, *J* = 8.4, 2.7 Hz, 1H, CH–SO_2_), 3.25 (dt, *J* = 12.3, 3.3 Hz, 1H, CH–N), 2.73 (s, 3H, CH_3_), 2.00–1.24 (m, 8H, 4CH_2_). ^13^C-NMR (75 MHz, DMSO-*d_6_*): δ (ppm) 167.52, 158.95, 155.83 (d, ^1^*J*_FC_ = 243.83 Hz, F-Ph), 138.64 (d, ^2^*J*_FC_ = 35.78 Hz), 135.09, 127.43 (d, ^3^*J*_FC_ = 7.43 Hz), 127.09, 125.21, 120.79 (q, ^1^*J*_FC_ = 269.63 Hz, CF_3_), 116.62, 116.36, 61.37, 45.37, 30.61, 24.29, 21.59, 19.48, 18.97. EIMS, *m/z* 465.48 (M). Elemental analysis for C_18_H_19_F_4_N_3_O_3_S_2_: found C 46.45, H 4.11, N 9.03; calcd C 46.29, H 4.32, N 9.21.

*N-(3-fluorophenyl)-2-(2-methyl-4-trifluoromethyl-5-thiazolecarboxylamino]-cyclohexylsulfonamide* III-11. White solid, yield: 81%. mp 159.0~160.1 °C. ^1^H-NMR (300 MHz, DMSO-*d_6_*): 9.99 (s, 1H, NH–SO_2_), 8.95 (d, *J* = 9.0 Hz, 1H, NH–C=O), 7.39–6.86 (m, 4H, C_6_H_4_), 4.69 (dd, *J* = 7.8, 2.4 Hz, 1H, CH–SO_2_), 3.38–3.35 (m, 1H, CH–N) (overlap with water peak), 2.74 (s, 3H, CH_3_), 2.05–1.20 (m, 8H, 4CH_2_). ^13^C-NMR (75 MHz, DMSO-*d*_6_): δ (ppm) 167.49, 162.89 (d, ^1^*J*_FC_ = 241.20 Hz, F-Ph), 158.84, 140.64 (d, ^3^*J*_FC_ = 10.65 Hz), 138.71 (d, ^2^*J*_FC_ = 35.63 Hz), 135.11, 131.49 (d, ^3^*J*_FC_ = 9.45 Hz), 115.17, 110.42 (d, ^2^*J*_FC_ = 20.70 Hz), 106.30, 105.96, 60.45, 45.46, 30.49, 24.04, 21.75, 19.51, 18.97. EIMS, *m/z* 465.48 (M). Elemental analysis for C_18_H_19_F_4_N_3_O_3_S_2_: found C 46.45, H 4.11, N 9.03; calcd C 46.57, H 3.96, N 8.91.

*N-(2-chlorophenyl)-2-(2-methyl-4-trifluoromethyl-5-thiazolecarboxylamino]-cyclohexylsulfonamide* III-12. White solid, yield: 85%. mp 188.8~189.8 °C. ^1^H-NMR (300 MHz, DMSO-*d*_6_): δ (ppm) 9.34 (s, 1H, NH–SO_2_), 8.92 (d, *J* = 9.0 Hz, 1H, NH–C=O), 7.53–7.21 (m, 4H, C_6_H_4_), 4.70 (dd, *J* = 8.7, 2.1 Hz, 1H, CH–SO_2_), 3.29 (dt, *J* = 11.1, 3.9 Hz, 1H, CH–N), 2.73 (s, 3H, CH_3_), 2.04–1.24 (m, 8H, 4CH_2_). ^13^C-NMR (75 MHz, DMSO-*d*_6_): δ (ppm) 167.53, 159.02, 135.13, 134.57, 130.30, 128.50, 128.30, 127.75, 127.62, 122.58, 118.99, 62.38, 45.38, 30.71, 24.41, 21.66, 19.42, 18.97. EIMS, *m/z* 481.93 (M). Elemental analysis for C_18_H_19_ClF_3_N_3_O_3_S_2_: found C 44.86, H 3.97, N 8.72; calcd C 44.69, H 3.75, N 8.94.

*N-(3-chlorophenyl)-2-(2-methyl-4-trifluoromethyl-5-thiazolecarboxylamino]-cyclohexylsulfonamide* III-13. White solid, yield: 82%. mp 160.5~161.8 °C. ^1^H-NMR (300 MHz, DMSO-*d*_6_): δ (ppm) 10.10 (d, *J* = 31.8 Hz, 1H, NH–SO_2_), 8.89 (dd, *J* = 14.7, 8.4 Hz, 1H, NH–C=O), 7.86–7.37 (m, 4H, C_6_H_4_), 4.72-4.10 (m, 1H, CH–SO_2_), 3.36–3.28 (m, 1H, CH–N) (overlap with water peak), 2.71 (d, *J* = 10.2 Hz, 3H, CH_3_), 2.08-1.23 (m, 8H, 4CH_2_). ^13^C-NMR (75 MHz, DMSO-*d*_6_): δ (ppm) 167.82, 158.86, 157.93, 139.93, 134.58, 134.27, 127.23, 127.01, 117.47, 117.33, 109.52, 63.96, 48.44, 32.34, 26.84, 24.06, 21.70, 19.01. EIMS, *m/z* 481.93 (M). Elemental analysis for C_18_H_19_ClF_3_N_3_O_3_S_2_: found C 44.86, H 3.97, N 8.72; calcd C 45.01, H 4.20, N 8.59.

*N-(4-chlorophenyl)-2-(2-methyl-4-trifluoromethyl-5-thiazolecarboxylamino]-cyclohexylsulfonamide* III-14. White solid, yield: 84%. mp 184.0~185.1 °C. ^1^H-NMR (300 MHz, DMSO-*d*_6_): δ (ppm) 9.87 (s, 1H, NH–SO_2_), 8.93 (d, *J* = 9.0 Hz, 1H, NH–C=O), 7.41–7.21 (m, 4H, C_6_H_4_), 4.67 (dd, *J* = 8.7, 2.7 Hz, 1H, CH–SO_2_), 3.27 (dt, *J* = 12.0, 3.9 Hz, 1H, CH–N), 2.74 (s, 3H, CH_3_), 2.09-1.18 (m, 8H, 4CH_2_). ^13^C-NMR (151 MHz, DMSO-*d*_6_): δ (ppm) 167.50, 158.84, 138.71 (q, ^2^*J*_FC_ = 35.79 Hz), 137.73, 135.10, 129.70 (two carbon atoms), 128.08, 121.37 (two carbon atoms), 120.80 (q, ^1^*J*_FC_ = 271.50 Hz, CF_3_), 60.28, 45.45, 30.53, 24.08, 21.71, 19.48, 18.99. EIMS, *m/z* 481.93 (M). Elemental analysis for C_18_H_19_ClF_3_N_3_O_3_S_2_: found C 44.86, H 3.97, N 8.72; calcd C 44.98, H 3.77, N 8.50.

*N-(2-bromophenyl)-2-(2-methyl-4-trifluoromethyl-5-thiazolecarboxylamino]-cyclohexylsulfonamide***III-15**. White solid, yield: 85%. mp 173.9~174.0 °C. ^1^H-NMR (300 MHz, DMSO-*d*_6_): δ (ppm) 9.24 (s, 1H, NH–SO_2_), 8.92 (d, *J* = 9.3 Hz, 1H, NH–C=O), 7.69–7.15 (m, 4H, C_6_H_4_), 4.71 (dd, *J* = 8.7, 2.1 Hz, 1H, CH–SO_2_), 3.32–3.28 (m, 1H, CH–N) (overlap with water peak), 2.73 (s, 3H, CH_3_), 2.10–1.29 (m, 8H, 4CH_2_). ^13^C-NMR (75 MHz, DMSO-*d*_6_): δ (ppm) 167.51, 159.00, 138.59 (q, ^2^*J*_FC_ = 35.78 Hz), 135.95, 135.12, 133.53, 128.86, 128.19 (d, ^3^*J*_FC_ = 13.28 Hz), 120.79 (q, ^1^*J*_FC_ = 269.70 Hz, CF_3_), 119.65, 109.99, 62.65, 45.39, 30.75, 24.45, 21.71, 19.40, 18.97. EIMS, *m/z* 526.39 (M). Elemental analysis for C_18_H_19_BrF_3_N_3_O_3_S_2_: found C 41.07, H 3.64, N 7.98; calcd C 40.95, H 3.51, N 8.14.

*N-(3-bromophenyl)-2-(2-methyl-4-trifluoromethyl-5-thiazolecarboxylamino]-cyclohexylsulfonamide***III-16**. White solid, yield: 86%. mp 196.0~196.9 °C. ^1^H-NMR (300 MHz, DMSO-*d*_6_): δ (ppm) 9.96 (s, 1H, NH–SO_2_), 8.94 (d, *J* = 9.0 Hz, 1H, NH–C=O), 7.40–7.21 (m, 4H, C_6_H_4_), 4.68 (dd, *J* = 8.1, 2.1 Hz, 1H, CH–SO_2_), 3.32–3.31 (m, 1H, CH–N) (overlap with water peak), 2.74 (s, 3H, CH_3_), 2.08–1.24 (m, 8H, 4CH_2_). ^13^C-NMR (151 MHz, DMSO-*d*_6_): δ (ppm) 167.49, 158.84, 140.50, 138.71 (q, ^2^*J*_FC_ = 35.79 Hz), 135.10, 131.75, 126.62, 122.48, 121.76, 120.80 (q, ^1^*J*_FC_ = 271.50 Hz, CF_3_), 118.20, 60.68, 45.48, 30.51, 24.03, 21.77, 19.50, 18.98. EIMS, *m/z* 526.39 (M). Elemental analysis for C_18_H_19_BrF_3_N_3_O_3_S_2_: found C 41.07, H 3.64, N 7.98; calcd C 41.26, H 3.81, N 8.06.

*N-(4-bromophenyl)-2-(2-methyl-4-trifluoromethyl-5-thiazolecarboxylamino]-cyclohexylsulfonamide* III-17. White solid, yield: 83%. mp 198.1~199.1 °C. ^1^H-NMR (300 MHz, DMSO-*d*_6_): δ (ppm) 9.88 (s, 1H, NH–SO_2_), 8.94 (d, *J* = 9.0 Hz, 1H, NH–C=O), 7.53–7.15 (m, 4H, C_6_H_4_), 4.67 (dd, *J* = 8.1, 2.4 Hz, 1H, CH–SO_2_), 3.28 (dt, *J* = 12.0, 3.6 Hz, 1H, CH–N), 2.74 (s, 3H, CH_3_), 2.07-1.15 (m, 8H, 4CH_2_). ^13^C-NMR (151 MHz, DMSO-*d*_6_): δ (ppm) 167.50, 158.85, 138.72 (q, ^2^*J*_FC_ = 35.79 Hz), 138.19, 135.10, 132.59 (two carbon atoms), 121.66 (two carbon atoms), 120.80 (q, ^1^*J*_FC_ = 271.20 Hz, CF_3_), 116.07, 60.29, 45.44, 30.53, 24.08, 21.71, 19.48, 18.98. EIMS, *m/z* 526.39 (M). Elemental analysis for C_18_H_19_BrF_3_N_3_O_3_S_2_: found C 41.07, H 3.64, N 7.98; calcd C 40.89, H 3.47, N 7.69.

*N-(3-cyanophenyl)-2-(2-methyl-4-trifluoromethyl-5-thiazolecarboxylamino]-cyclohexylsulfonamide* III-18. White solid, yield: 88%. mp 196.0~197.0 °C. ^1^H-NMR (300 MHz, DMSO-*d*_6_): δ (ppm) 10.17 (s, 1H, NH–SO_2_), 8.95 (d, *J* = 9.1 Hz, 1H, NH–C=O), 7.58–7.49 (m, 4H, C_6_H_4_), 4.71 (dd, *J* = 8.1, 2.4 Hz, 1H, CH–SO_2_), 3.43 (dt, *J* = 12.3, 3.3 Hz, 1H, CH–N), 2.74 (s, 3H, CH_3_), 2.08–1.23 (m, 8H, 4CH_2_). ^13^C-NMR (151 MHz, DMSO-*d*_6_): δ (ppm) 167.49, 158.84, 139.80, 138.74 (q, ^2^*J*_FC_ = 35.79 Hz), 135.08, 131.20, 127.39, 124.01, 121.89, 120.80 (q, ^1^*J*_FC_ = 271.35 Hz, CF_3_), 118.99, 112.60, 60.92, 45.42, 30.51, 24.06, 21.71, 19.44, 18.97. EIMS, *m/z* 472.5 (M). Elemental analysis for C_19_H_19_F_3_N_4_O_3_S_2_: found C 48.30, H 4.05, N 11.86; calcd C 48.18, H 3.89, N 11.67. 

*N-(3-fluorophenyl)-2-[1-(3-fluorophenyl)-5-trifluoromethyl-1H-pyrazole-4-carboxylamino]-cyclohexylsulfonamide.* III-19. White solid, yield: 70%. mp 167.7~170.5 °C. ^1^H-NMR (600 MHz, DMSO-*d*_6_): δ (ppm) 10.00 (s, 1H, NH-SO_2_), 8.51 (d, *J* = 8.9 Hz, 1H, NH–C=O), 8.08 (s, 1H, pyrazole-H), 7.68–6.88 (m, 8H, C_6_H_4_ + C_6_H_4_), 4.70 (dd, *J*= 7.2, 3.0 Hz, 1H, CH–SO_2_), 3.40 (dt, *J* = 11.4, 3.6 Hz, 1H, CH–N), 2.16–1.24 (m, 8H, 4CH_2_). ^13^C-NMR (151 MHz, DMSO-*d*_6_): δ (ppm) 162.83 (d, ^1^*J*_FC_ = 242.81 Hz, F-Ph), 162.10 (d, ^1^*J*_FC_ = 246.73 Hz, F-Ph), 160.07, 140.71, 140.60 (d, ^3^*J*_FC_ = 10.87 Hz), 140.28 (d, ^3^*J*_FC_ = 10.27 Hz), 131.62 (d, ^3^*J*_FC_ = 8.91 Hz), 131.41 (d, ^3^*J*_FC_ = 9.66 Hz), 129.97 (d, ^2^*J*_FC_ = 39.11 Hz), 122.76, 122.09, 119.64 (q, ^1^*J*_FC_ = 270.44 Hz, CF_3_), 117.52 (d, ^2^*J*_FC_ = 20.84 Hz), 115.01, 114.05 (d, ^2^*J*_FC_ = 24.46 Hz), 110.30 (d, ^2^*J*_FC_ = 20.84 Hz), 106.00 (d, ^2^*J*_FC_ = 25.67 Hz), 60.41, 45.10, 30.52, 23.90, 21.92, 19.77. EIMS, *m/z* 527.10 (M-H^+^). Elemental analysis for C_23_H_21_F_5_N_4_O_3_S: found C 52.27, H 4.01, N 10.60; calcd C 52.03, H 4.21, N 10.43.

*N-(2-chlorophenyl)-2-[1-(3-fluorophenyl)-5-trifluoromethyl-1H-pyrazole-4-carboxylamino]-cyclohexylsulfonamide.* III-20. White solid, yield: 84%. mp 112.1~115.3 °C. ^1^H-NMR (600 MHz, DMSO-*d*_6_): δ (ppm) 9.38 (s, 1H, NH-SO_2_), 8.48 (d, *J* = 9.0 Hz, 1H, NH–C=O), 8.05(s, 1H, pyrazole-H), 7.68–7.23 (m, 8H, C_6_H_4_ + C_6_H_4_), 4.73 (dd, *J*= 7.8, 2.4 Hz, 1H, CH–SO_2_), 3.31(m, 1H, CH–N) (overlap with water peak), 2.15–1.31 (m, 8H, 4CH_2_). ^13^C-NMR (151 MHz, DMSO-*d*_6_): δ (ppm) 162.09 (d, ^1^*J*_FC_ = 246.58 Hz, F-Ph), 160.27, 140.70, 140.25 (d, ^3^*J*_FC_ = 10.42 Hz), 134.55, 131.62 (d, ^3^*J*_FC_ = 9.21 Hz), 130.24, 129.91 (q, ^2^*J*_FC_ = 38.96 Hz), 128.28 (d, ^3^*J*_FC_ = 14.50 Hz), 127.51 (d, ^3^*J*_FC_ = 8.61 Hz), 122.75, 122.10, 119.62 (q, ^1^*J*_FC_ = 270.74 Hz, CF_3_), 117.59, 117.45, 114.14, 113.98, 62.37, 44.96, 30.77, 24.32, 21.81, 19.62. EIMS, *m/z* 543.00 (M-H^+^). Elemental analysis for C_23_H_21_ClF_4_N_4_O_3_S: found C 50.69, H 3.88, N 10.28; calcd C 50.56, H 4.03, N 10.42.

*N-(4-chlorophenyl)-2-[1-(3-fluorophenyl)-5-trifluoromethyl-1H-pyrazole-4-carboxylamino]-cyclohexylsulfonamide.* III-21. White solid, yield: 81%. mp 156.9~158.8 °C. ^1^H-NMR (600 MHz, DMSO-*d*_6_): δ (ppm) 9.88 (s, 1H, NH-SO_2_), 8.50 (d, *J* = 8.9 Hz, 1H, NH–C=O), 8.07 (s, 1H, pyrazole-H), 7.68–7.23 (m, 8H, C_6_H_4_ + C_6_H_4_), 4.69 (dd, *J*= 7.2, 3.0 Hz, 1H, CH–SO_2_), 3.32-3.31 (m, 1H, CH–N) (overlap with water peak), 2.16–1.24 (m, 8H, 4CH_2_). ^13^C-NMR (151 MHz, DMSO-*d*_6_): δ(ppm) 162.10(d, ^1^*J*_FC_ = 246.58 Hz, F-Ph), 160.08, 140.74, 140.28 (d, ^3^*J*_FC_ = 10.27 Hz), 137.68, 131.61 (d, ^3^*J*_FC_ = 9.06 Hz), 129.97 (q, ^2^*J*_FC_ = 39.26 Hz), 129.62, 127.95, 122.74, 122.10, 121.22, 119.63 (q, ^1^*J*_FC_ = 270.74 Hz, CF_3_), 117.58, 117.44, 114.13, 113.96, 60.22, 45.02, 30.59, 23.97, 21.84, 19.70. EIMS, *m/z* 543.00 (M-H^+^). Elemental analysis for C_23_H_21_ClF_4_N_4_O_3_S: found C 50.69, H 3.88, N 10.28; calcd C 50.45, H 4.01, N 10.51.

*N-(2-bromophenyl)-2-[1-(3-fluorophenyl)-5-trifluoromethyl-1H-pyrazole-4-carboxylamino]-cyclohexylsulfonamide.* III-22. White solid, yield: 92%. mp 101.5~104.8 °C. ^1^H-NMR (600 MHz, DMSO-*d*_6)_: δ (ppm) 9.28 (s, 1H, NH-SO_2_), 8.47 (d, *J* = 9.0 Hz, 1H, NH–C=O), 8.05 (s, 1H, pyrazole-H), 7.68–7.16 (m, 8H, C_6_H_4_ + C_6_H_4_), 4.73 (dd, *J*= 8.4, 3.0 Hz, 1H, CH–SO_2_), 3.31 (m, 1H, CH–N) (overlap with water peak), 2.11–1.32 (m, 8H, 4CH_2_). ^13^C-NMR (151 MHz, DMSO-*d*_6_): δ (ppm) 162.10 (d, ^1^*J*_FC_ = 246.58 Hz, F-Ph), 160.25, 140.70, 140.26 (d, ^3^*J*_FC_ = 10.27 Hz), 133.47, 131.62 (d, ^3^*J*_FC_ = 9.06 Hz), 129.91 (q, ^2^*J*_FC_ = 39.26 Hz) 128.79, 128.02 (d, ^2^*J*_FC_ = 18.72 Hz), 122.75, 122.12, 119.63 (q, ^1^*J*_FC_ = 270.74 Hz, CF_3_), 119.48, 117.58, 117.44, 114.14, 113.97, 62.65, 44.98, 30.81, 24.34, 21.88, 19.61. EIMS, *m/z* 589.00 (M+H^+^). Elemental analysis for C_23_H_21_BrF_4_N_4_O_3_S: found C 46.87, H 3.59, N 9.51; calcd C 46.98, H 3.33, N 9.72.

*N-(3-bromophenyl)-2-[1-(3-fluorophenyl)-5-trifluoromethyl-1H-pyrazole-4-carboxylamino]-cyclohexylsulfonamide.* III-23. White solid, yield: 85%. mp 97.7~100.2 °C. ^1^H-NMR (600 MHz, DMSO-*d*_6_): δ (ppm) 9.97 (s, 1H, NH-SO_2_), 8.51 (d, *J* = 9.0 Hz, 1H, NH–C=O), 8.07 (s, 1H, pyrazole-H), 7.68–7.23 (m, 8H, C_6_H_4_ + C_6_H_4_), 4.70 (dd, *J*= 7.8, 3.0 Hz, 1H, CH–SO_2_), 3.39 (dt, *J* = 12.0, 3.6 Hz, 1H, CH–N), 2.18–1.24 (m, 8H, 4CH_2_). ^13^C-NMR (151 MHz, DMSO-*d*_6_): δ (ppm) 162.10 (d, ^1^*J*_FC_ = 246.58 Hz, F-Ph), 160.07, 140.71, 140.28 (d, ^3^*J*_FC_ = 10.12 Hz), 131.63 (d, ^3^*J*_FC_ = 11.63 Hz), 129.97 (q, ^2^*J*_FC_ = 39.11 Hz), 126.50, 122.75, 122.40, 122.08, 121.60, 119.63 (q, ^1^*J*_FC_ = 270.74 Hz, CF_3_), 118.07, 117.59, 117.45, 114.13, 113.97, 60.60, 45.06, 30.54, 23.91, 21.92, 19.73. EIMS, *m/z* 589.00 (M+H^+^). Elemental analysis for C_23_H_21_BrF_4_N_4_O_3_S: found C 46.87, H 3.59, N 9.51; calcd C 46.59, H 3.74, N 9.32.

*N-(4-bromophenyl)-2-[1-(3-fluorophenyl)-5-trifluoromethyl-1H-pyrazole-4-carboxylamino]-cyclohexylsulfonamide.* III-24. White solid, yield: 91%. mp 169.7~171.2 °C. ^1^H-NMR (600 MHz, DMSO-*d*_6_): δ (ppm) 10.00 (s, 1H, NH-SO_2_), 8.51 (d, *J* = 9.0 Hz, 1H, NH–C=O), 8.08(s, 1H, pyrazole-H), 7.68–6.88 (m, 8H, C_6_H_4_ + C_6_H_4_), 4.70 (dd, *J*= 7.2, 3.0 Hz, 1H, CH–SO_2_), 3.40 (dt, *J* = 11.4, 3.6 Hz, 1H, CH–N), 2.18-1.24 (m, 8H, 4CH_2_). ^13^C-NMR (151 MHz, DMSO-*d*_6_): δ (ppm) 162.10 (d, ^1^*J*_FC_ = 246.58 Hz, F-Ph), 160.08, 140.74, 140.28 (d, ^3^*J*_FC_ = 10.12 Hz), 138.14, 132.52, 131.61 (d, ^3^*J*_FC_ = 9.06 Hz), 129.97 (q, ^2^*J*_FC_ = 38.96 Hz), 122.74, 122.09, 121.51, 119.64 (q, ^1^*J*_FC_ = 270.74 Hz, CF_3_), 117.58, 117.44, 115.94, 114.13, 113.97, 60.23, 45.01, 30.59, 23.97, 21.84, 19.69. EIMS, *m/z* 589.00 (M+H^+^). Elemental analysis for C_23_H_21_BrF_4_N_4_O_3_S: found C 46.87, H 3.59, N 9.51; calcd C 46.65, H 3.48, N 9.29.

*N-(2,4,5-trifluorophenyl)-2-[1-(3-fluorophenyl)-5-trifluoromethyl-1H-pyrazole-4-carboxylamino]-cyclohexylsulfonamide.* III-25. White solid, yield: 70%. mp 100.0~121.1 °C. ^1^H-NMR (600 MHz, DMSO-*d*_6_): δ (ppm) 9.81 (s, 1H, NH-SO_2_), 8.47 (s, 1H, NH–C=O), 8.06(s, 1H, pyrazole-H), 7.66–7.39 (m, 6H, C_6_H_4_ + C_6_H_2_), 4.69 (s, 1H, CH–SO_2_), 3.41 (s, 1H, CH–N), 2.14–1.32 (m, 8H, 4CH_2_). ^13^C-NMR (151 MHz, DMSO-*d*_6_): δ (ppm) 162.10 (d, ^1^*J*_FC_ = 246.43 Hz, F-Ph), 160.14, 151.17 (dd, *J*_FC_ = 245.38, 9.66 Hz, F-Ph), 146.92 (dt, *J*_FC_ = 244.77, 11.17 Hz, F-Ph), 145.99 (dd, *J*_FC_ = 239.03, 12.68 Hz, F-Ph), 140.68, 140.27 (d, ^3^*J*_FC_ = 10.42 Hz), 131.61 (d, ^3^*J*_FC_ = 9.06 Hz), 129.96 (q, ^2^*J*_FC_ = 39.26 Hz), 122.73, 122.02, 119.61 (q, ^1^*J*_FC_ = 270.74 Hz, CF_3_), 117.51 (d, ^2^*J*_FC_ = 21.14 Hz), 114.75 (d, ^2^*J*_FC_ = 21.14 Hz), 114.03 (d, ^2^*J*_FC_ = 25.67 Hz), 106.76 (d, ^2^*J*_FC_ = 22.20 Hz), 106.58(d, ^2^*J*_FC_ = 22.05 Hz), 61.60, 44.91, 30.60, 24.13, 21.68, 19.63. EIMS, *m/z* 563.00 (M-H^+^). Elemental analysis for C_23_H_21_F_7_N_4_O_3_S: found C 48.94, H 3.39, N 9.93; calcd C 48.76, H 3.46, N 9.71.

*N-(3-chlorophenyl)-2-[1-(3-fluorophenyl)-5-trifluoromethyl-1H-pyrazole-4-carboxylamino]-cyclohexylsulfonamide.* III-26. White solid, yield: 83%. mp 137.2~140.8 °C. ^1^H-NMR (300 MHz, DMSO-*d*_6_): δ (ppm) 10.07 (s, 1H, NH-SO_2_), 8.50 (d, *J* = 9.0 Hz, 1H, NH–C=O), 8.05(s, 1H, pyrazole-H), 7.86–7.38 (m, 8H, C_6_H_4_ + C_6_H_4_), 4.73 (dd, *J*= 8.7, 3.0 Hz, 1H, CH–SO_2_), 3.45–3.38 (m, 1H, CH–N) (overlap with water peak), 2.16–1.36 (m, 8H, 4CH_2_). ^13^C-NMR (151 MHz, DMSO-*d*_6_): δ (ppm) 162.09 (d, ^1^*J*_FC_ = 246.58 Hz, F-Ph), 160.09, 140.67, 140.27 (d, ^3^*J*_FC_ = 10.12 Hz), 139.62, 134.56, 134.23, 131.61 (d, ^3^*J*_FC_ = 9.21 Hz), 129.93 (q, ^2^*J*_FC_ = 38.81 Hz), 127.21, 127.00, 122.75, 122.10, 119.62 (q, ^1^*J*_FC_ = 270.59 Hz, CF_3_), 117.51 (d, ^2^*J*_FC_ = 20.84 Hz), 114.06 (d, ^2^*J*_FC_ = 24.61 Hz), 109.43, 62.19, 44.93, 30.74, 24.21, 21.87, 19.63. EIMS, *m/z* 544.10 (M). Elemental analysis for C_23_H_21_ClF_4_N_4_O_3_S: found C 50.69, H 3.88, N 10.28; calcd C 50.77, H 3.74, N 10.39.

*N-(2-fluorophenyl)-2-[1-(3-fluorophenyl)-5-trifluoromethyl-1H-pyrazole-4-carboxylamino]-cyclohexylsulfonamide.* III-27. White solid, yield: 77%. mp 128.3~131.5 °C. ^1^H-NMR (600 MHz, DMSO-*d*_6_): δ (ppm) 9.56 (s, 1H, NH-SO_2_), 8.47 (d, *J* = 8.4 Hz, 1H, NH–C=O), 8.05(s, 1H, pyrazole-H), 7.68–7.17 (m, 8H, C_6_H_4_ + C_6_H_4_), 4.70 (dd, *J*= 8.4, 3.6 Hz, 1H, CH–SO_2_), 3.29 (dt, *J* = 12.0, 3.6 Hz, 1H, CH–N), 2.16–1.27 (m, 8H, 4CH_2_). ^13^C-NMR (151 MHz, DMSO-*d*_6_): δ (ppm) 162.10 (d, ^1^*J*_FC_ = 246.43 Hz, F-Ph), 160.23, 155.68 (d, ^1^*J*_FC_ = 245.68 Hz, F-Ph), 140.72, 140.27 (d, ^3^*J*_FC_ = 10.27 Hz), 131.61 (d, ^3^*J*_FC_ = 9.06 Hz), 129.92 (d, ^2^*J*_FC_ = 39.11 Hz), 127.26 (d, ^3^*J*_FC_ = 7.40 Hz), 126.85, 125.29 (d, ^3^*J*_FC_ = 12.68 Hz), 125.14, 122.74, 122.11, 119.63 (q, ^1^*J*_FC_ = 270.74 Hz, CF_3_), 117.51 (d, ^2^*J*_FC_ = 21.14 Hz), 116.42 (d, ^2^*J*_FC_ = 19.93 Hz), 114.05 (d, ^2^*J*_FC_ = 24.61 Hz), 61.36, 44.98, 30.67, 24.19, 21.75, 19.68. EIMS, *m/z* 527.10 (M-H^+^). Elemental analysis for C_23_H_21_F_5_N_4_O_3_S: found C 52.27, H 4.01, N 10.60; calcd C 52.45, H 3.88, N 10.81.

*N-(3-cyanophenyl)-2-(1-methyl-3-difluoromethyl-1H-pyrazole-4-carboxylamino]-cyclohexylsulfonamide* III-28. White solid, yield: 71%. mp 213.8~215.2 °C. ^1^H-NMR (600 MHz, DMSO-*d*_6_):δ (ppm) 10.13 (s, 1H, NH-SO_2_), 8.36 (s, 1H, pyrazole-H), 7.67(d, *J* = 8.4 Hz, 1H, NH–C=O), 7.52–7.44 (m, 4H, C_6_H_4_), 7.26 (t, *J* = 54 Hz, 1H, HCF_2_), 4.64 (dd, *J* = 7.2, 3.0 Hz, 1H, CH–SO_2_), 3.93 (s, 3H, CH_3_), 3.52 (dt, *J* = 11.4, 3.6 Hz, 1H, CH–N), 2.16–1.29 (m, 8H, 4CH_2_). ^13^C-NMR (151 MHz, DMSO-*d*_6_): δ (ppm) 161.06, 144.78 (t, ^2^*J*_FC_ = 22.80 Hz), 139.71, 133.46, 130.96, 127.06, 123.73, 121.63, 118.87, 116.34, 112.38, 110.16 (t, ^1^*J*_FC_ = 234.05 Hz, HCF_2_), 61.11, 44.92, 40.40, 30.38, 23.84, 22.06, 19.91. EIMS, *m/z* 436.10 (M-H^+^). Elemental analysis for C_19_H_21_F_2_N_5_O_3_S: found C 52.17, H 4.84, N 16.01; calcd C 52.37, H 4.69, N 15.83.

*N-(2-fluorophenyl)-2-(1-methyl-3-difluoromethyl-1H-pyrazole-4-carboxylamino]-cyclohexylsulfonamide* III-29. White solid, yield: 65%. mp 164.7~165.9 °C. ^1^H-NMR (600 MHz, DMSO-*d*_6_): δ (ppm) 9.55 (s, 1H, NH-SO_2_), 8.38 (s, 1H, pyrazole-H), 7.67 (d, *J* = 8.4 Hz, 1H, NH–C=O), 7.42–7.13 (m, 4H, C_6_H_4_), 7.33(t, *J* = 53.4 Hz, 1H, HCF_2_), 4.65 (dd, *J* = 7.8, 3.6 Hz, 1H, CH–SO_2_), 3.93 (s, 3H, CH_3_), 3.30 (dt, *J* = 12.0, 3.6 Hz, 1H, CH–N), 2.13–1.30 (m, 8H, 4CH_2_). ^13^C-NMR (151 MHz, DMSO-*d*_6_): δ (ppm) 161.24, 155.57 (d, ^1^*J*_FC_ = 245.68 Hz, F-Ph), 144.67 (t, ^2^*J*_FC_ = 23.41 Hz), 133.56, 127.17 (d, ^3^*J*_FC_ = 7.55 Hz), 126.66, 125.27 (d, ^3^*J*_FC_ = 12.68 Hz), 125.10 (d, ^3^*J*_FC_ = 3.47 Hz), 116.43, 116.30, 110.17 (t, ^1^*J*_FC_ = 234.20 Hz, HCF_2_), 61.32, 44.87, 40.41, 30.49, 24.05, 21.93, 19.89. EIMS, *m/z* 429.10 (M-H^+^). Elemental analysis for C_18_H_21_F_3_N_4_O_3_S: found C 50.23, H 4.92, N 13.02; calcd C 49.98, H 5.21, N 13.19.

*N-(2-bromophenyl)-2-(1-methyl-3-difluoromethyl-1H-pyrazole-4-carboxylamino]-cyclohexylsulfonamide* III-30. White solid, yield: 90%. mp 183.3~184.1°C. ^1^H-NMR (600 MHz, DMSO-*d*_6_): δ (ppm) 9.25 (s, 1H, NH-SO_2_), 8.39 (s, 1H, pyrazole-H), 7.69 (d, *J* = 8.4 Hz, 1H, NH–C=O), 7.66–7.13 (m, 4H, C_6_H_4_), 7.25(t, *J* = 54.6 Hz, 1H, HCF_2_), 4.69 (dd, *J* = 7.8, 3.6 Hz, 1H, CH–SO_2_), 3.93 (s, 3H, CH_3_), 3.37 (dt, *J* = 11.4, 3.6 Hz, 1H, CH–N), 2.09–1.36 (m, 8H, 4CH_2_). ^13^C-NMR (151 MHz, DMSO-*d*_6_): δ (ppm) 161.28, 144.69 (t, ^2^*J*_FC_ = 23.25 Hz), 135.92, 133.56, 133.44, 128.75, 127.84, 127.77, 119.27, 116.41, 110.17 (t, ^1^*J*_FC_ = 234.20 Hz, HCF_2_), 62.56, 44.86, 40.81, 30.62, 24.20, 22.04, 19.83. EIMS, *m/z* 491.00 (M+H^+^). Elemental analysis for C_18_H_21_BrF_2_N_4_O_3_S: found C 44.00, H 4.31, N 11.40; calcd C 44.21, H 4.19, N 11.56.

*N-(2-chlorophenyl)-2-(1-methyl-3-difluoromethyl-1H-pyrazole-4-carboxylamino]-cyclohexylsulfonamide.* III-31. White solid, yield: 67%. mp 190.8~192.3 °C. ^1^H-NMR (300 MHz, DMSO-*d*_6_): δ(ppm) 9.37 (s, 1H, NH-SO_2_), 8.39 (s, 1H, pyrazole-H), 7.70 (d, *J* = 8.4 Hz, 1H, NH–C=O), 7.51–7.19 (m, 4H, C_6_H_4_), 7.24(t, *J* = 54.3 Hz, 1H, HCF_2_), 4.68 (dd, *J* = 7.5, 3.3 Hz, 1H, CH–SO_2_), 3.94 (s, 3H, CH_3_), 3.33–3.31 (m, 1H, CH–N) (overlap with water peak), 2.16–1.34 (m, 8H, 4CH_2_). ^13^C-NMR (151 MHz, DMSO-*d*_6_): δ (ppm) 161.29, 144.68 (t, ^2^*J*_FC_ = 23.56 Hz), 134.54, 133.57, 130.20, 128.17, 128.15, 127.35, 127.26, 116.41, 110.17 (t, ^1^*J*_FC_ = 234.20 Hz, HCF_2_), 62.31, 44.86, 40.42, 30.58, 24.17, 21.99, 19.84. EIMS, *m/z* 445.10 (M-H^+^). Elemental analysis for C_18_H_21_ClF_2_N_4_O_3_S: found C 48.38, H 4.74, N 12.54; calcd C 48.21, H 4.92, N 12.34.

*N-(3-fluorophenyl)-2-(1-methyl-3-difluoromethyl-1H-pyrazole-4-carboxylamino]-cyclohexylsulfonamide.* III-32. White solid, yield: 84%. mp 185.7~187.1 °C. ^1^H-NMR (600 MHz, DMSO-*d*_6_): δ (ppm) 9.97 (s, 1H, NH-SO_2_), 8.38 (s, 1H, pyrazole-H), 7.70 (d, *J* = 8.4 Hz, 1H, NH–C=O), 7.33–6.84 (m, 4H, C_6_H_4_), 7.27(t, *J* = 54.6 Hz, 1H, HCF_2_), 4.62 (dd, *J*= 7.2, 3.6 Hz, 1H, CH–SO_2_), 3.94 (s, 3H, CH_3_), 3.43 (dt, *J* = 11.4, 3.6 Hz, 1H, CH–N), 2.13–1.26 (m, 8H, 4CH_2_). ^13^C-NMR (151 MHz, DMSO-*d*_6_): δ (ppm) 162.79 (d, ^1^*J*_FC_ = 242.81 Hz, F-Ph), 161.11, 144.76 (t, ^2^*J*_FC_ = 23.25 Hz), 140.57 (d, ^3^*J*_FC_ = 10.72 Hz), 133.51, 131.29 (d, ^3^*J*_FC_ = 9.66 Hz), 116.42, 114.95, 110.18 (t, ^1^*J*_FC_ = 234.50 Hz, HCF_2_), 106.01, 105.84, 60.48, 45.02, 40.42, 30.36, 23.78, 22.11, 20.01. EIMS, *m/z* 429.10 (M-H^+^). Elemental analysis for C_18_H_21_F_3_N_4_O_3_S: found C 50.23, H 4.92, N 13.02; calcd C 50.44, H 5.16, N 12.89.

*N-(4-bromophenyl)-2-(1-methyl-3-difluoromethyl-1H-pyrazole-4-carboxylamino]-cyclohexylsulfonamide* III-33. White solid, yield: 84%. mp 229.9~231.1 °C. ^1^H-NMR (600 MHz, DMSO-*d*_6_): δ (ppm) 9.86 (s, 1H, NH-SO_2_), 8.37 (s, 1H, pyrazole-H), 7.69 (d, *J* = 8.4 Hz, 1H, NH–C=O), 7.47–7.14 (m, 4H, C_6_H_4_), 7.27 (t, *J* = 54.0 Hz, 1H, HCF_2_), 4.61 (dd, *J*= 7.8, 3.6 Hz, 1H, CH–SO_2_), 3.94 (s, 3H, CH_3_), 3.34 (m, 1H, CH–N) (overlap with water peak), 2.11–1.23 (m, 8H, 4CH_2_). ^13^C-NMR (151 MHz, DMSO-*d*_6_): δ (ppm) 161.12, 144.71 (t, ^2^*J*_FC_ = 23.25 Hz), 138.11, 133.53, 132.42 (two carbon atoms), 121.41 (two carbon atoms), 116.42, 115.85, 110.20 (t, ^1^*J*_FC_ = 234.35 Hz, HCF_2_), 60.30, 44.94, 40.40, 30.40, 23.83, 22.03, 19.93. EIMS, *m/z* 491.00 (M+H^+^). Elemental analysis for C_18_H_21_BrF_2_N_4_O_3_S: found C 44.00, H 4.31, N 11.40; calcd C 43.87, H 4.19, N 11.56.

*N-(3-bromophenyl)-2-(1-methyl-3-difluoromethyl-1H-pyrazole-4-carboxylamino]-cyclohexylsulfonamide.* III-34. White solid, yield: 79%. mp 221.1~223.8 °C. ^1^H-NMR (600 MHz, DMSO-*d*_6_): δ (ppm) 9.93 (s, 1H, NH-SO_2_), 8.37 (s, 1H, pyrazole-H), 7.69(d, *J* = 8.4 Hz, 1H, NH–C=O), 7.35–7.18 (m, 4H, C_6_H_4_), 7.27(t, *J* = 54.0 Hz, 1H, HCF_2_), 4.62 (dd, *J*= 7.2, 3.6 Hz, 1H, CH–SO_2_), 3.94 (s, 3H, CH_3_), 3.42 (dt, *J* = 11.4, 3.6 Hz, 1H, CH–N), 2.13–1.29 (m, 8H, 4CH_2_). ^13^C-NMR (151 MHz, DMSO-*d*_6_): δ (ppm) 161.10, 144.87 (t, ^2^*J*_FC_ = 23.41 Hz), 140.43, 133.48, 131.53, 126.35, 122.33, 121.51, 117.97, 116.40, 110.18 (t, ^1^*J*_FC_ = 234.05 Hz, HCF_2_), 60.74, 45.00, 40.43, 30.37, 23.78, 22.13, 19.99. EIMS, *m/z* 491.00 (M+H^+^). Elemental analysis for C_18_H_21_BrF_2_N_4_O_3_S: found C 44.00, H 4.31, N 11.40; calcd C 44.19, H 4.52, N 11.27.

*N-(2,4,5-trifluorophenyl)-2-(1-methyl-3-difluoromethyl-1H-pyrazole-4-carboxylamino]-cyclohexylsulfonamide* III-35. White solid, yield: 89%. mp 68.5~70.4 °C. ^1^H-NMR (600 MHz, DMSO-*d*_6_): δ (ppm) 9.78 (s, 1H, NH-SO_2_), 8.37 (s, 1H, pyrazole-H), 7.66 (d, *J* = 8.4 Hz, 1H, NH–C=O), 7.64–7.43 (m, 2H, C_6_H_2_), 7.23(t, *J* = 54.6 Hz, 1H, HCF_2_), 4.63 (dd, *J*= 7.2, 3.6 Hz, 1H, CH–SO_2_), 3.93 (s, 3H, CH_3_), 3.42 (dt, *J* = 11.4, 3.6 Hz, 1H, CH–N), 2.11–1.33 (m, 8H, 4CH_2_). ^13^C-NMR (151 MHz, DMSO-*d*_6_): δ (ppm) 161.17, 150.94 (dd, ^1^*J*_FC_ = 245.38, 8.91 Hz, F-Ph), 146.78 (dt, *J*_FC_ = 244.47, 14.19 Hz, F-Ph), 145.95 (dd, ^1^*J*_FC_ = 242.36, 12.84 Hz, F-Ph) 144.70 (t, ^2^*J*_FC_ = 23.25 Hz), 133.51, 122.14, 116.30, 114.40 (d, ^2^*J*_FC_ = 22.20 Hz), 110.13 (t, ^1^*J*_FC_ = 234.35 Hz, HCF_2_), 106.57 (dd, *J*_FC_ = 26.58, 22.05 Hz), 61.73, 44.78, 40.41, 30.45, 24.02, 21.85, 19.80. EIMS, *m/z* 466.11 (M). Elemental analysis for C_18_H_21_F_5_N_4_O_3_S: found C 46.35, H 4.11, N 12.01; calcd C 46.52, H 3.98, N 12.24.

*N-(4-chlorophenyl)-2-(1-methyl-3-difluoromethyl-1H-pyrazole-4-carboxylamino]-cyclohexylsulfonamide.* III-36. White solid, yield: 55%. mp 255.7~257.0 °C. ^1^H-NMR (600 MHz, DMSO-*d*_6_): δ (ppm) 9.84 (s, 1H, NH-SO_2_), 8.37 (s, 1H, pyrazole-H), 7.68 (d, *J* = 8.4 Hz, 1H, NH–C=O), 7.34–7.19 (m, 4H, C_6_H_4_), 7.27(t, *J* = 54.0 Hz, 1H, HCF_2_), 4.61 (dd, *J*= 6.6, 3.0 Hz, 1H, CH–SO_2_), 3.94 (s, 3H, CH_3_), 3.35 (dt, *J* = 11.4, 3.0 Hz, 1H, CH–N), 2.14–1.25 (m, 8H, 4CH_2_). ^13^C-NMR (151 MHz, DMSO-*d*_6_): δ (ppm) 161.11, 144.72 (t, ^2^*J*_FC_ = 23.57 Hz), 137.67, 133.52, 129.53 (two carbon atoms), 127.86, 121.09 (two carbon atoms), 116.43, 110.20 (t, ^1^*J*_FC_ = 234.50 Hz, HCF_2_), 60.29, 44.94, 40.44, 30.41, 23.84, 22.04, 19.94. EIMS, *m/z* 447.10 (M+H^+^). Elemental analysis for C_18_H_21_ClF_2_N_4_O_3_S: found C 48.38, H 4.74, N 12.54; calcd C 48.54, H 4.51, N 12.70.

### 3.3. Fungicidal Activity Bioassays

The in vitro and in vivo fungicidal activities of all the target compounds against *B. cinerea* were tested by mycelium growth inhibition assay, spore germination experiment and tomato pot experiment, respectively. The *B. cinerea* strains (KZ-9, CY-09) were collected from different regions of Liaoning, China, and cultured on potato dextrose agar (PDA) for several generations. Boscalid and procymidone (with the purity more than 95%) are provided by the Shenyang Research Institute of the Chemical Industry.

#### 3.3.1. Evaluation of the Fungicidal Activity on *B. cinerea* by Mycelium Growth Experiments

Using the mycelium growth rate method, the compounds were dissolved in acetone and mixed with sterile molten PDA to obtain four concentrations of 50, 12.50, 3.125 and 0.78 μg/mL, poured them into sterile 90 mm diameter Petri dishes under aseptic conditions, respectively. A 5 mm *B. cinerea* plug was inoculated in the center of 90 mm PDA petri dish. Boscalid and procymidone were used as positive control agents while acetone was used as blank control. The EC_50_ value was calculated according to mycelium growth. [25]

#### 3.3.2. Evaluation of the Fungicidal Activity on *B. cinerea* by Spore Germination Experiments

The effect of target compounds III on spore germination against *B. cinerea* was determined by the concave slide method. The method was given in reference [25]. The commercial fungicide boscalid was used as the positive control, and the results were given in Table 3.

#### 3.3.3. In Vivo Fungicidal Activity against *B. cinerea* by Tomato Pot Experiments

The compound was first prepared to 5% emulsifiable concentrate (EC) and diluted with water into an aqueous solution of 200 μg/mL. Tomato seedlings at the 6–8 leaves stage on the main stem were selected as objects. Spraying the solution evenly on the tomato plants and upon naturally drying, a spore suspension of *B. cinerea* with 5 × 10^5^ spores per mL was evenly sprayed on the tomato seedlings. It was placed in a greenhouse at 20–25 °C, with humidity above 80%. Waiting for observation, using boscalid as control agents, and an emulsifiable concentrate without agent was the blank control and then disease incidence and index on tomato leaves were calculated. [25]

## 4. Conclusions

In conclusion, 36 novel sulfonamide compounds were designed and synthesized in this paper. The thiazole and pyrazole active groups were introduced by the method of active substructure splicing, and benzene ring (linked to NH) was optimized. In vitro and in vivo fungicidal activities against *B.cinerea* were evaluated, and some target compounds showed excellent activity. Moreover, the structure–activity relationship revealed that the compounds containing phenylpyrazole amide structure showed better activity effects, while fungicidal activity was highest at the meta-substituted fluorine atom on the benzene ring (linked to NH), such as compound III-19 showing notable in vitro fungicidal activity. Thus, the present results laid the foundation for further structural design and fungicide screening.

## Figures and Tables

**Table 1:** The EC_50_ values of target compounds III against *B. cinerea* (KZ-9) in vitro.

Compound	R_1_	R_2_		EC_50_ (μg/mL)
III-1	2-F	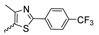		4.96
III-2	3-F	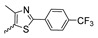		2.32
III-3	2-Cl	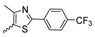		2.99
III-4	3-Cl	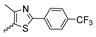		2.33
III-5	4-Cl	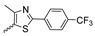		21.12
III-6	2-Br	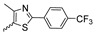		18.70
III-7	3-Br	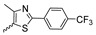		89.45
III-8	4-Br	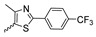		18.79
III-9	2,4,5-F	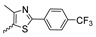		2.10
III-10	2-F	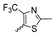		37.00
III-11	3-F	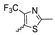		3.65
III-12	2-Cl	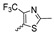		44.3
III-13	3-Cl	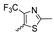		>100
III-14	4-Cl	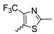		17.97
III-15	2-Br	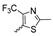		>100
III-16	3-Br	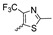		25.78
III-17	4-Br	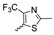		10.01
III-18	3-CN	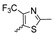		44.72
III-19	3-F	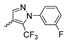		1.99
III-20	2-Cl	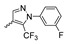		5.94
III-21	4-Cl	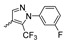		2.11
III-22	2-Br	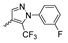		7.01
III-23	3-Br	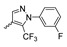		3.27
III-24	4-Br	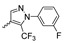		2.56
III-25	2,4,5-F	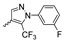		5.76
III-26	3-Cl	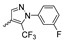		14.75
III-27	2-F	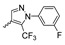		2.04
III-28	3-CN	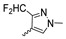		>100
III-29	2-F	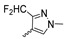		31.47
III-30	2-Br	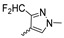		17.53
III-31	2-Cl	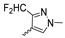		9.93
III-32	3-F	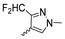		44.91
III-33	4-Br	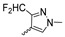		21.16
III-34	3-Br	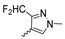		18.63
III-35	2,4,5-F	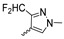		6.47
III-36	4-Cl	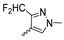		3.37
boscalid	/	/		1.72
procymidone	/	/		1.79

**Table 2:** The EC_50_ values of selected target compounds III against *B. cinerea* (CY-09) in vitro.

Compound	R_1_	R_2_	EC_50_ (μg/mL)
III-2	3-F	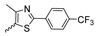	>100
III-3	2-Cl	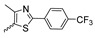	>100
III-4	3-Cl	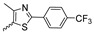	8.01
III-9	2,4,5-F	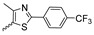	>100
III-19	3-F	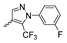	12.64
III-21	4-Cl	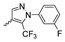	2.33
III-23	3-Br	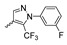	7.66
III-27	2-F	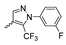	5.70
boscalid	/	/	2.25
procymidone	/	/	5.19

**Table 3:** Inhibition rate of target compounds III for spore germination at 10 μg/mL.

Compound	Inhibition Rate%	Compound	Inhibition Rate%
III-1	24.23	III-19	93.89
III-2	72.34	III-20	36.80
III-3	75.98	III-21	75.00
III-4	21.01	III-22	67.79
III-5	41.00	III-23	42.00
III-6	61.78	III-24	67.86
III-7	49.33	III-25	70.94
III-8	17.69	III-26	30.00
III-9	26.44	III-27	80.82
III-10	24.37	III-28	60.00
III-11	34.89	III-29	39.57
III-12	36.48	III-30	42.58
III-13	50.77	III-31	98.00
III-14	61.34	III-32	77.66
III-15	10.10	III-33	41.70
III-16	14.36	III-34	42.57
III-17	73.77	III-35	37.87
III-18	72.09	III-36	35.82
boscalid	95.97	/	/

**Table 4:** Control efficiency of specific target compounds against *B. cinerea* in tomato pot experiments.

Compd.	Control Effect%
III-2	14.15
III-3	52.33
III-19	78.00
III-21	89.80
III-27	87.90
boscalid	81.99

## Supplementary Materials

Supplementary Materials are available online at https://www.mdpi.com/1420-3049/24/14/2607/s1 including the ^1^H-NMR and ^13^C-NMR spectra of target compounds III and a detailed description of the crystal structure of III-31.
