# Supplementary material for: Synthesis, Fungicidal Activity and SAR of 2-Thiazolamide/Pyrazolamide-Cyclohexylsulfonamides against Botrytis cinerea
Source: Molecules. 2019 Jul 17;24(14):2607. doi: 10.3390/molecules24142607 (PMC6680688; doi:10.3390/molecules24142607)
Supplement: Supplementary file 1 [file molecules-24-02607-s001.pdf]

All ^{1}H-NMR spectra used dimethyl sulfoxide (DMSO-d_6) as solvent and tetramethylsilane (TMS) as internal standard. Solvent peak was at 2.5ppm and water peak was at 3.35ppm.

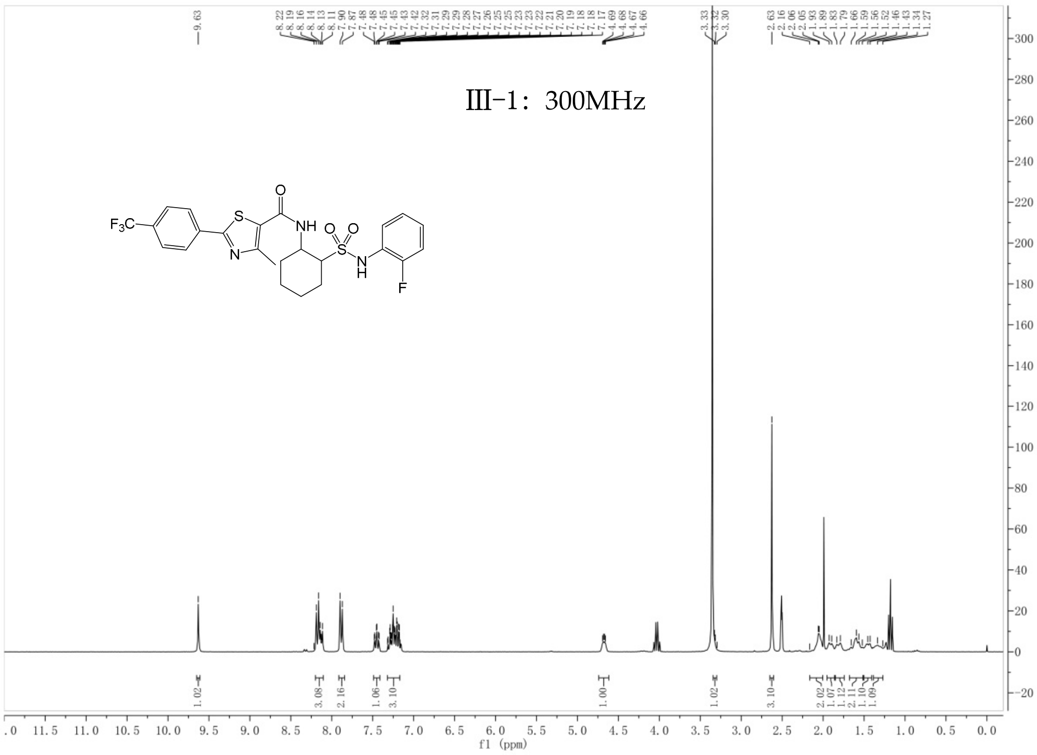

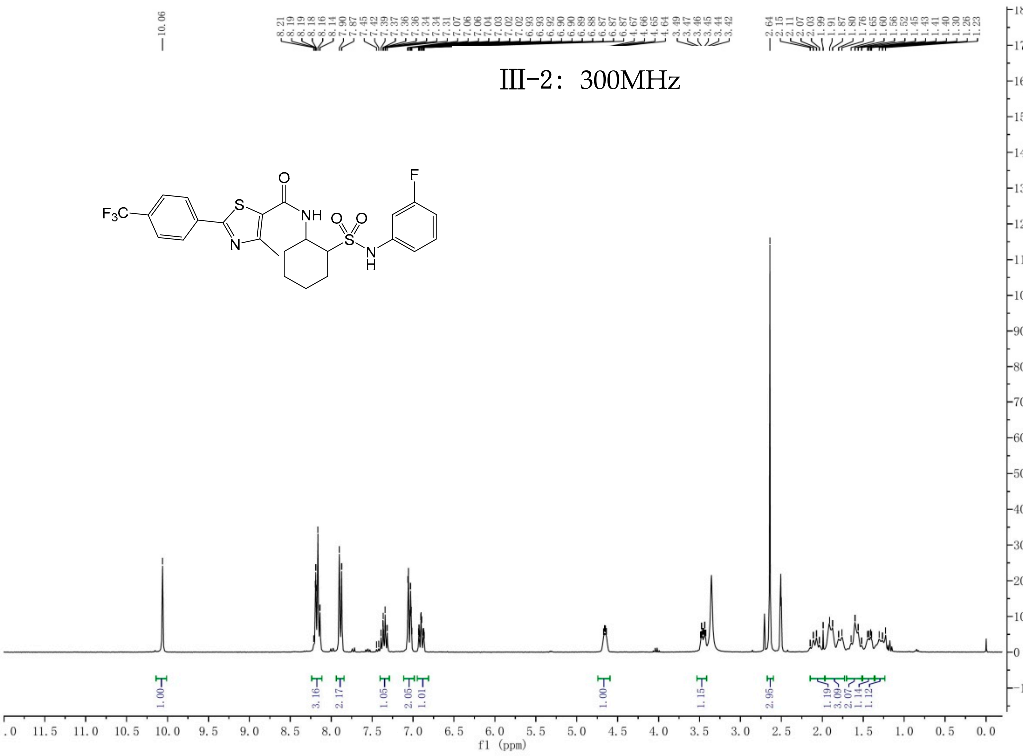

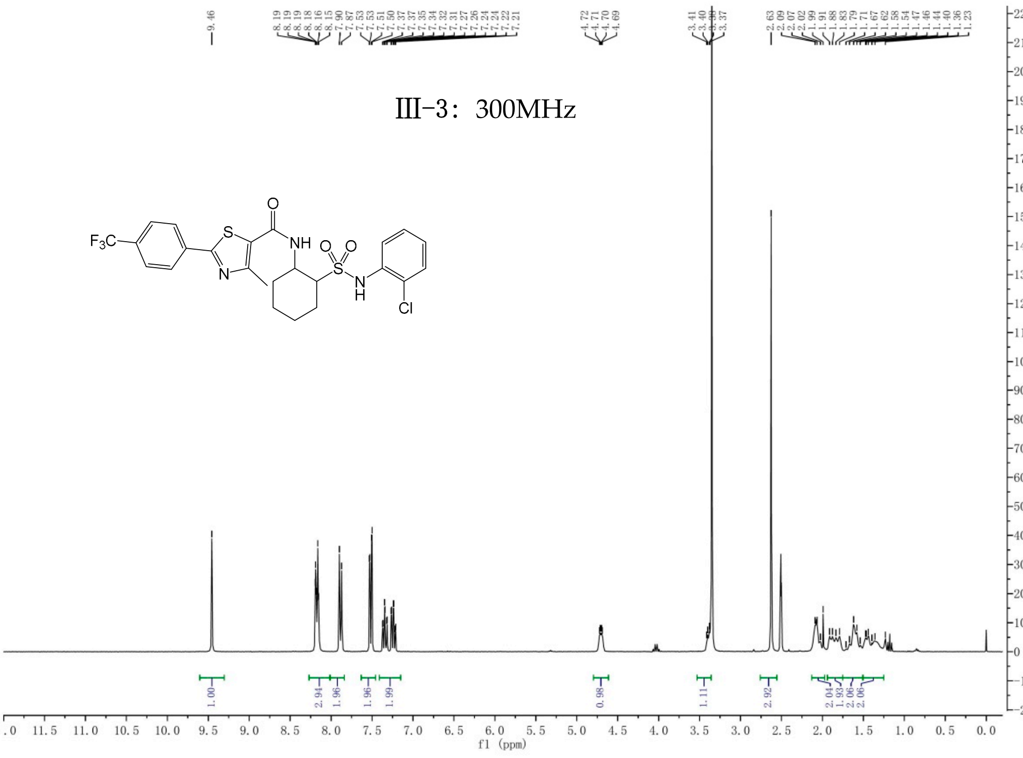
Ⅲ-3：300MHz
f1 (ppm)

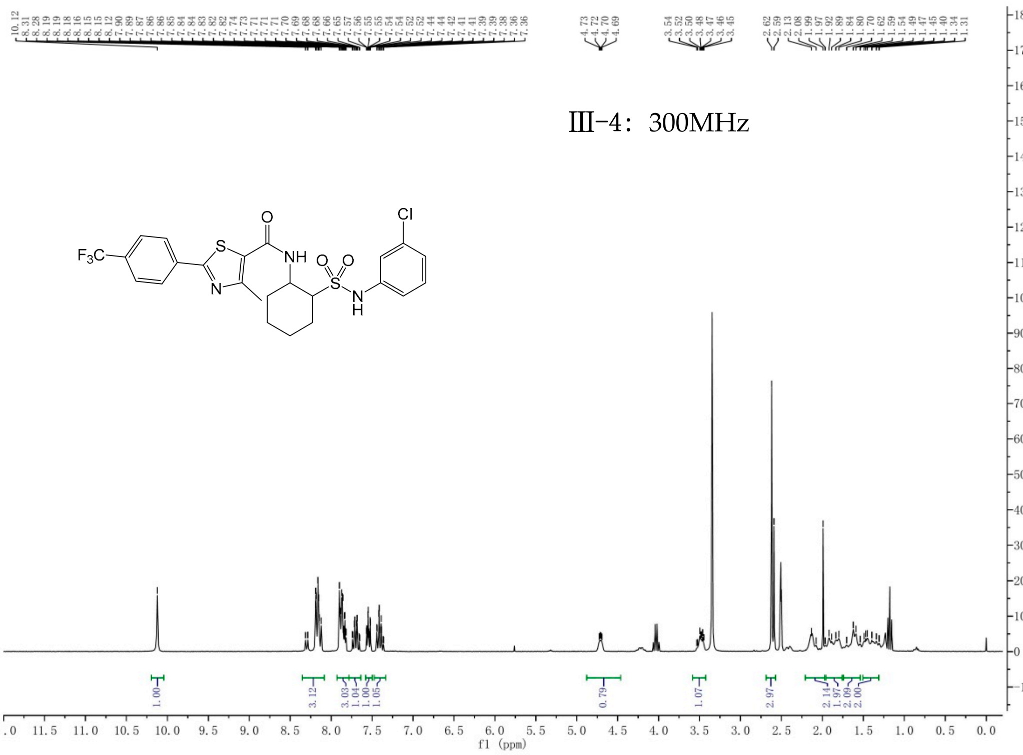
Ⅲ-4：300MHz
f1 (ppm)

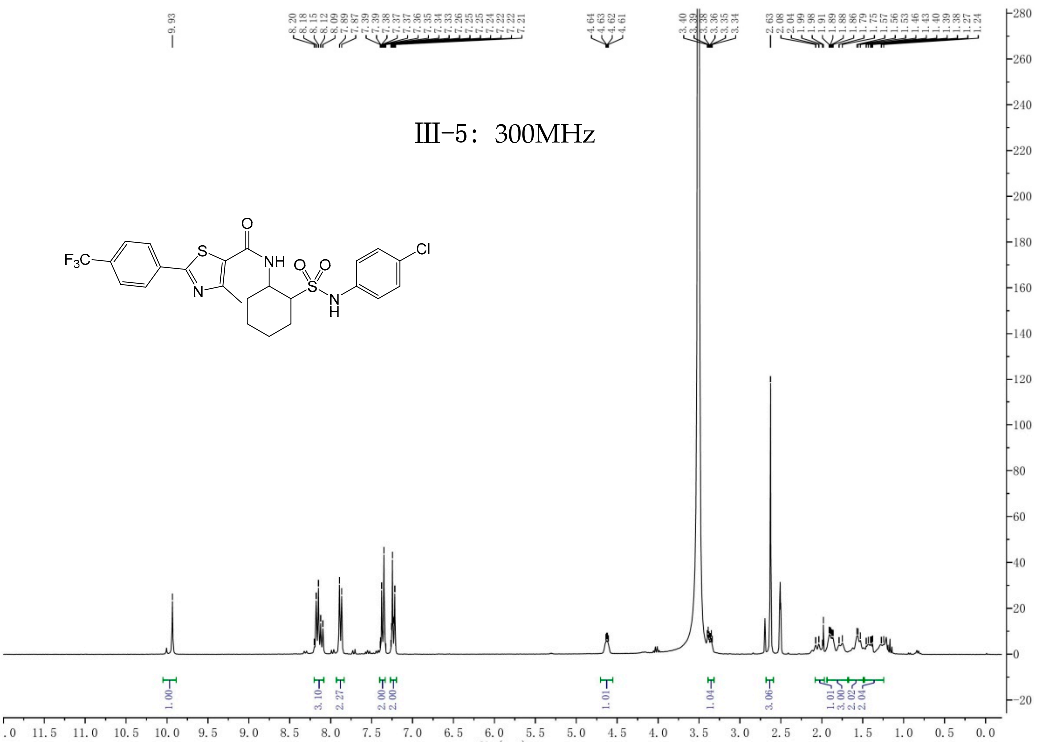
Ⅲ-5：300MHz
F3C
S
N
O
NH
O
S
O
N
H
Cl
f1 (ppm)

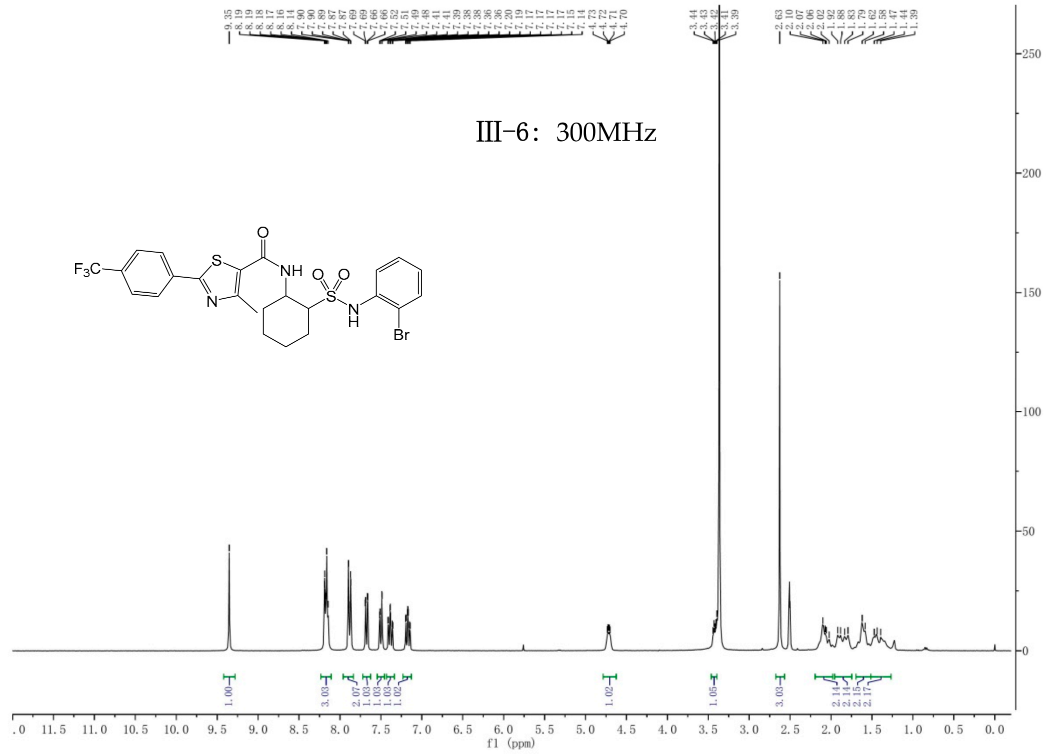
Ⅲ-6：300MHz
F3C
S
N
O
NH
O
S
O
N
H
Br
f1 (ppm)

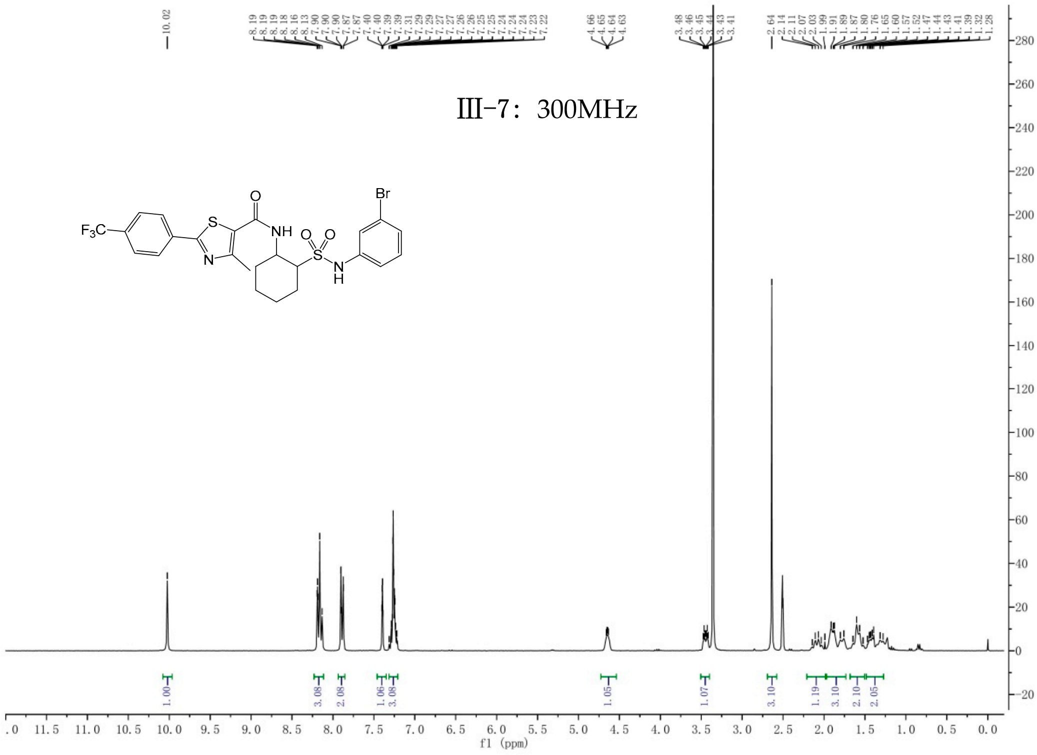
Ⅲ-7：300MHz
F3C
S
N
O
NH
S
O O
N
H
Br
f1 (ppm)

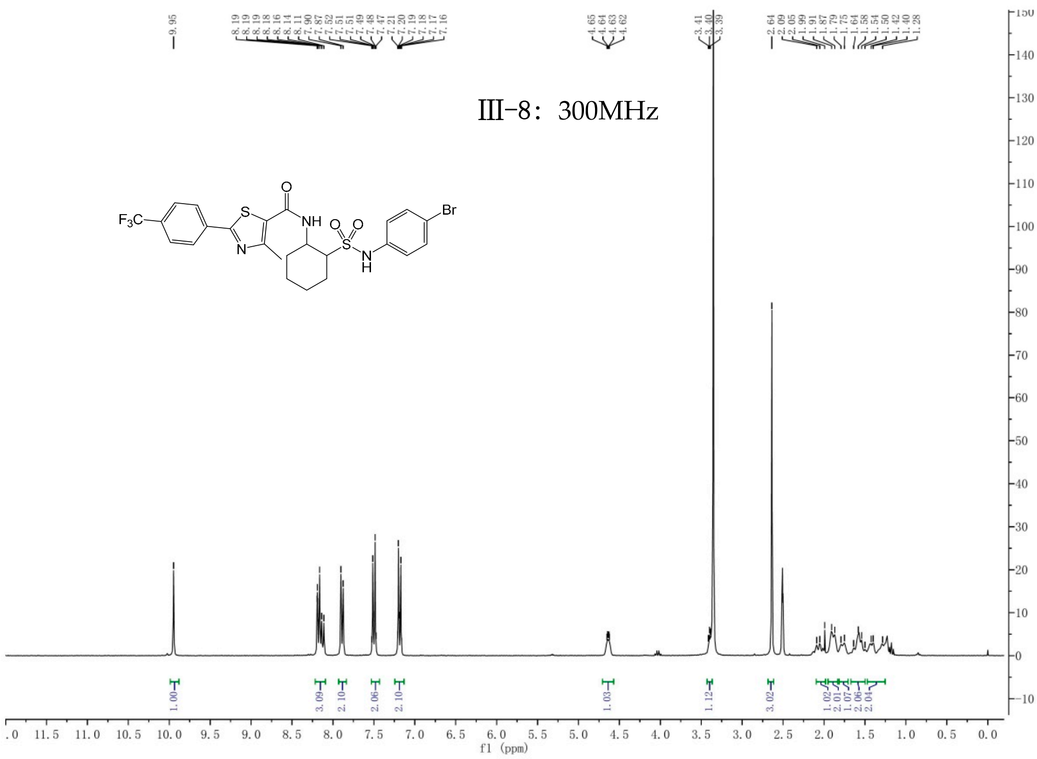
Ⅲ-8：300MHz
F3C
S
N
O
NH
S
O O
N
H
Br
f1 (ppm)

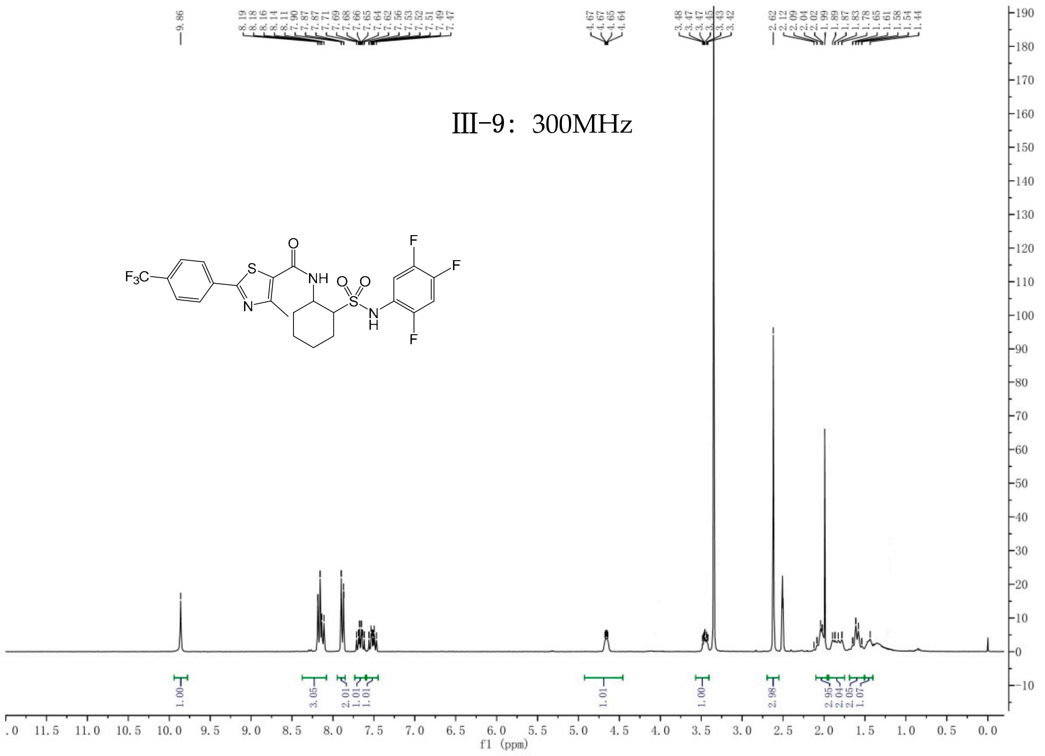
Ⅲ-9：300MHz
f1 (ppm)

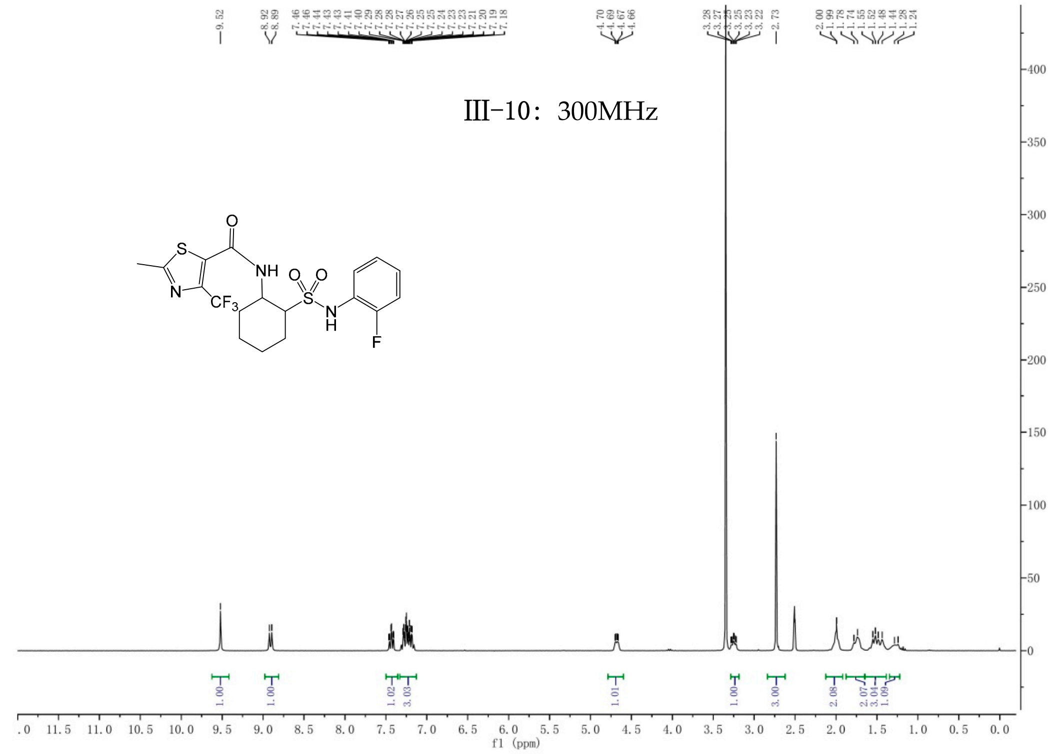
Ⅲ-10：300MHz
f1 (ppm)

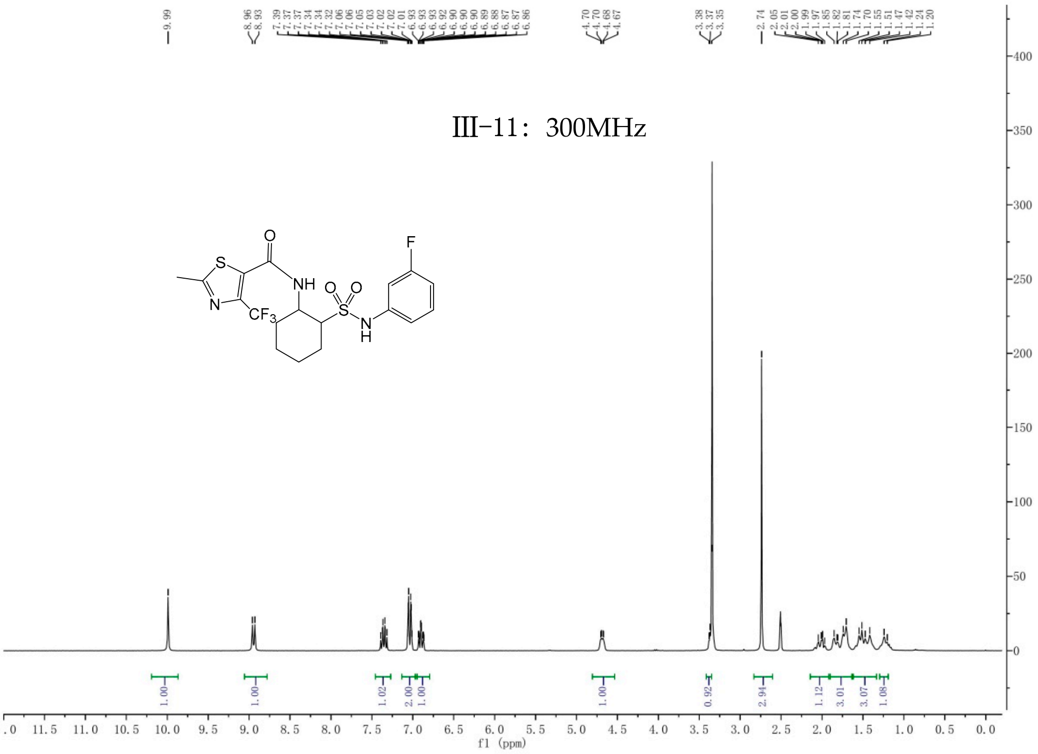
Ⅲ-11：300MHz
f1 (ppm)

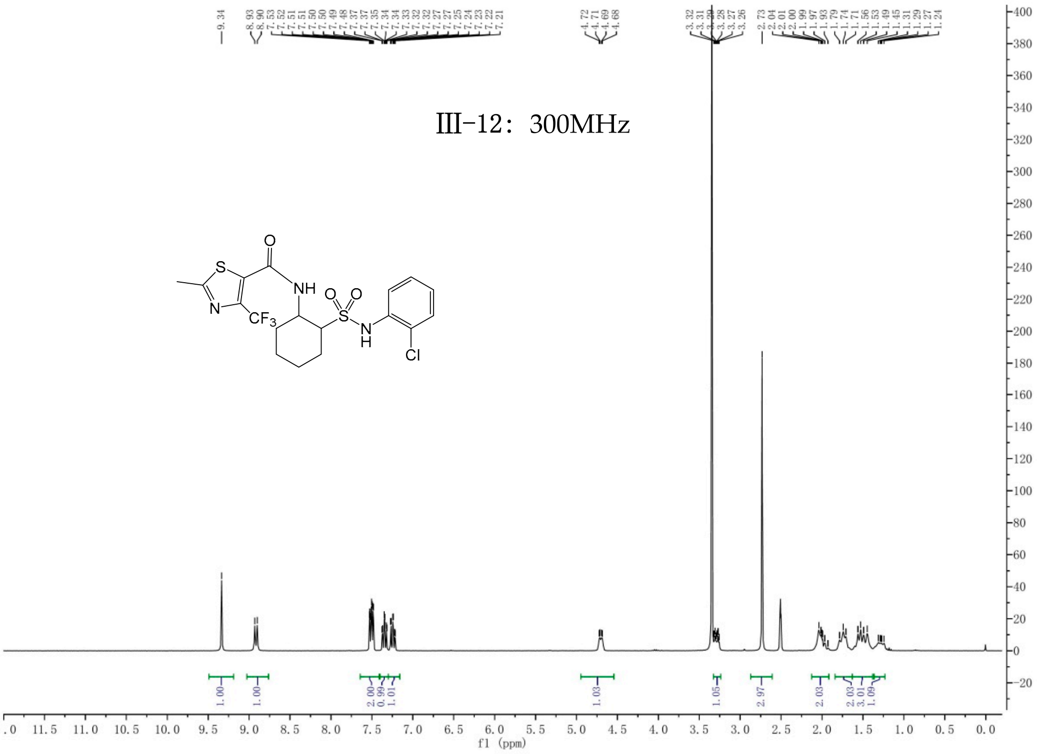
Ⅲ-12：300MHz
f1 (ppm)

Ⅲ-13：300MHz

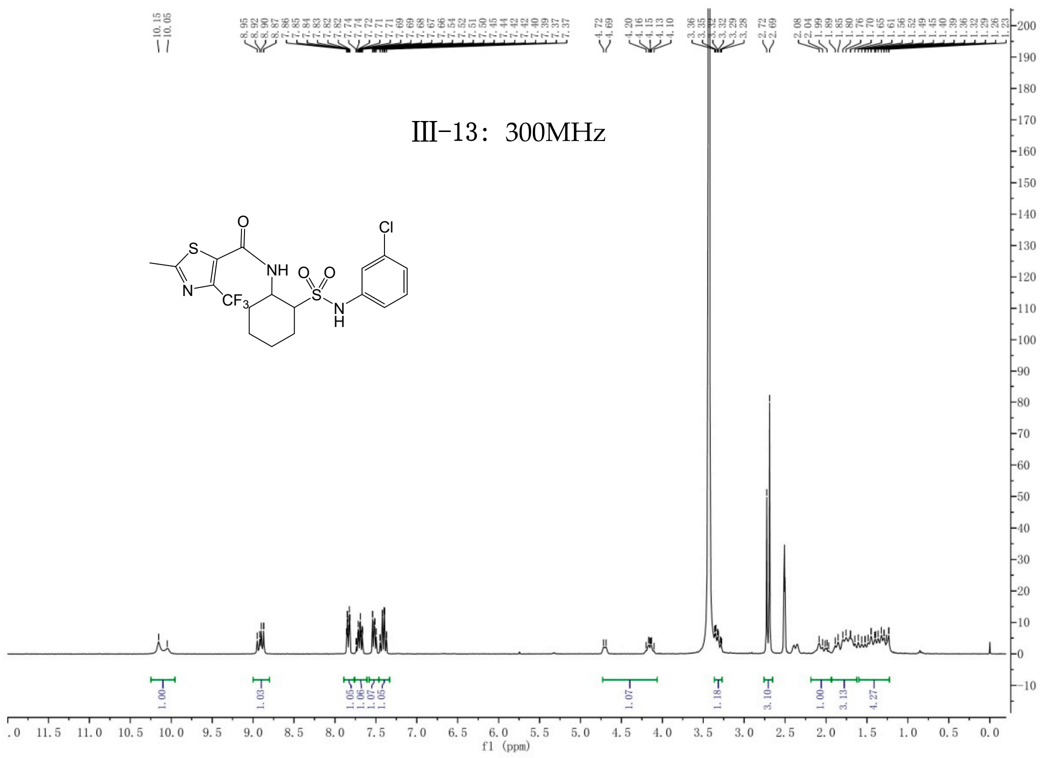

Ⅲ-14：300MHz

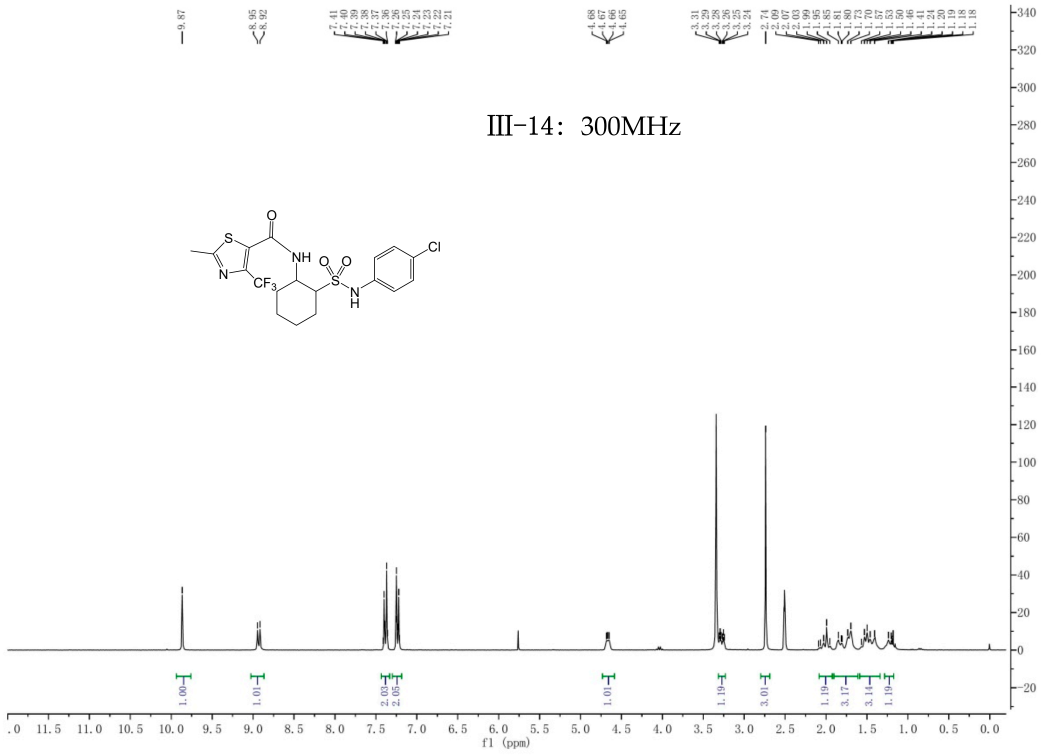

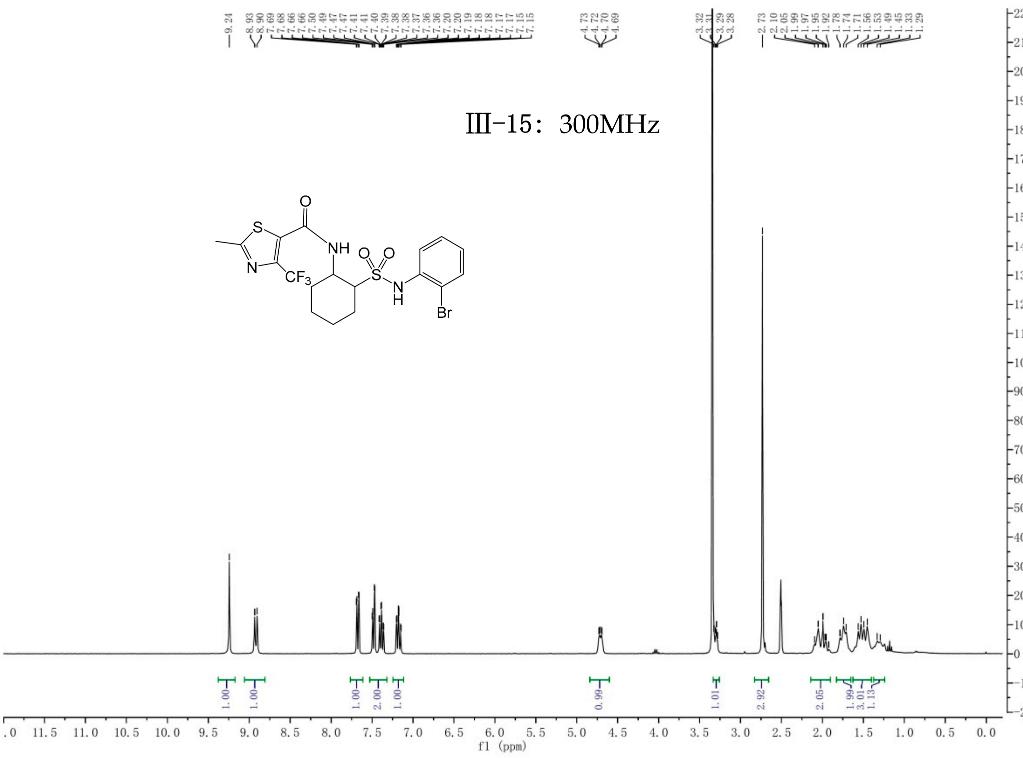
Ⅲ-15：300MHz
f1 (ppm)

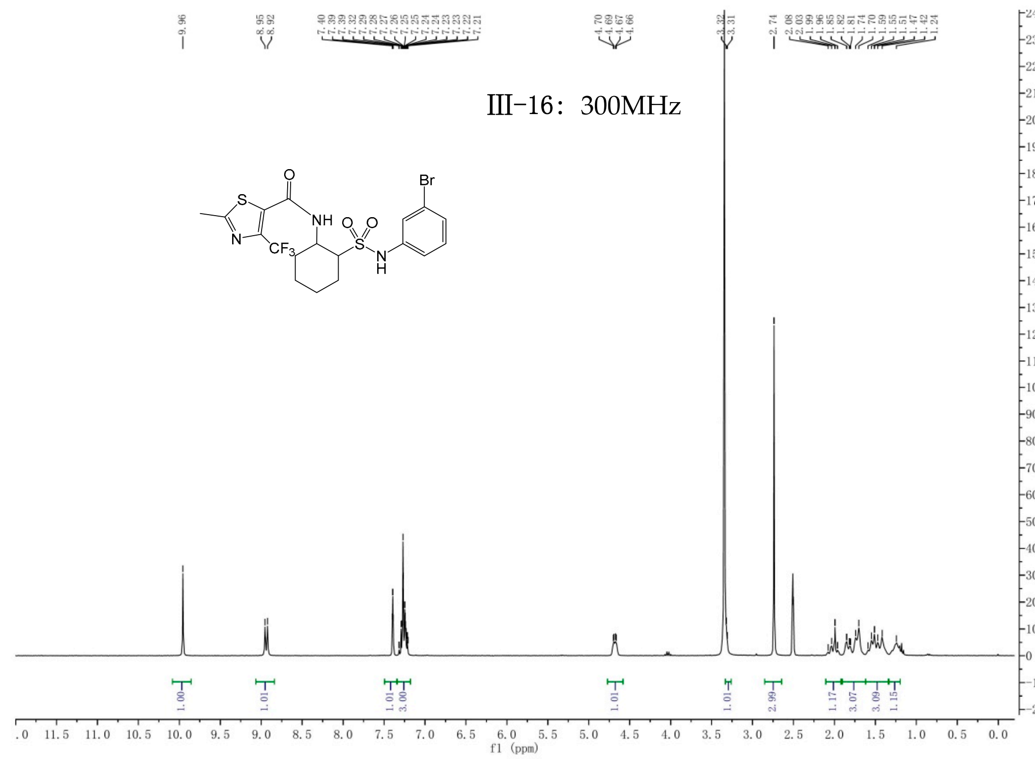
Ⅲ-16：300MHz
f1 (ppm)

Ⅲ-17：300MHz

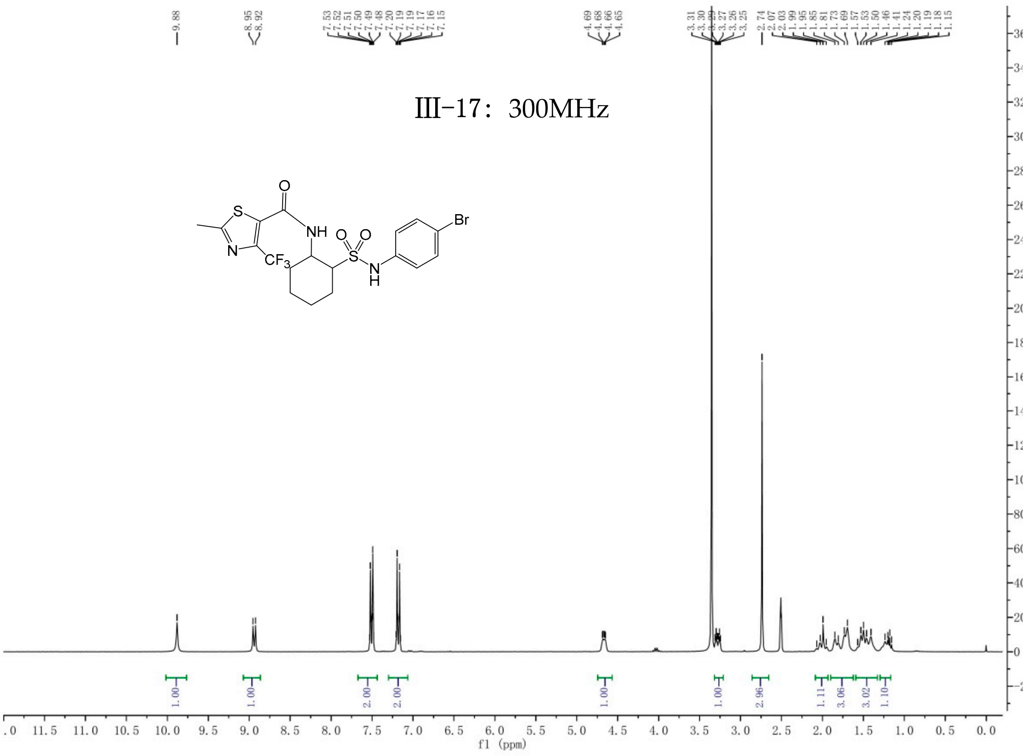

Ⅲ-18：300MHz

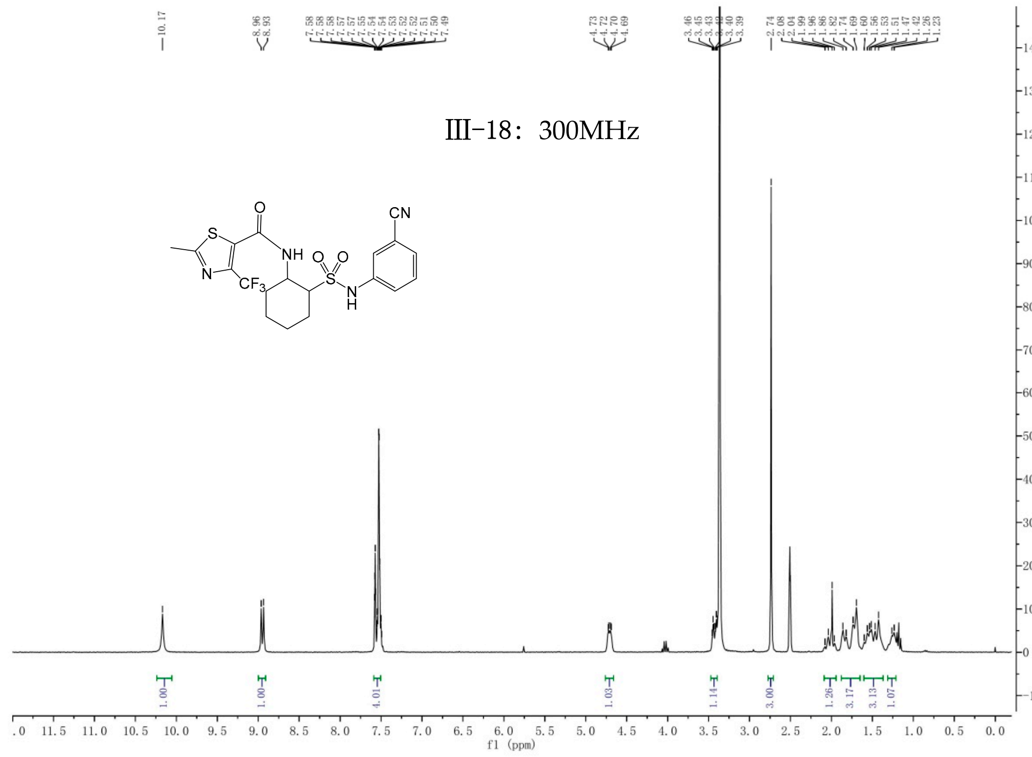

Ⅲ-19：600MHz

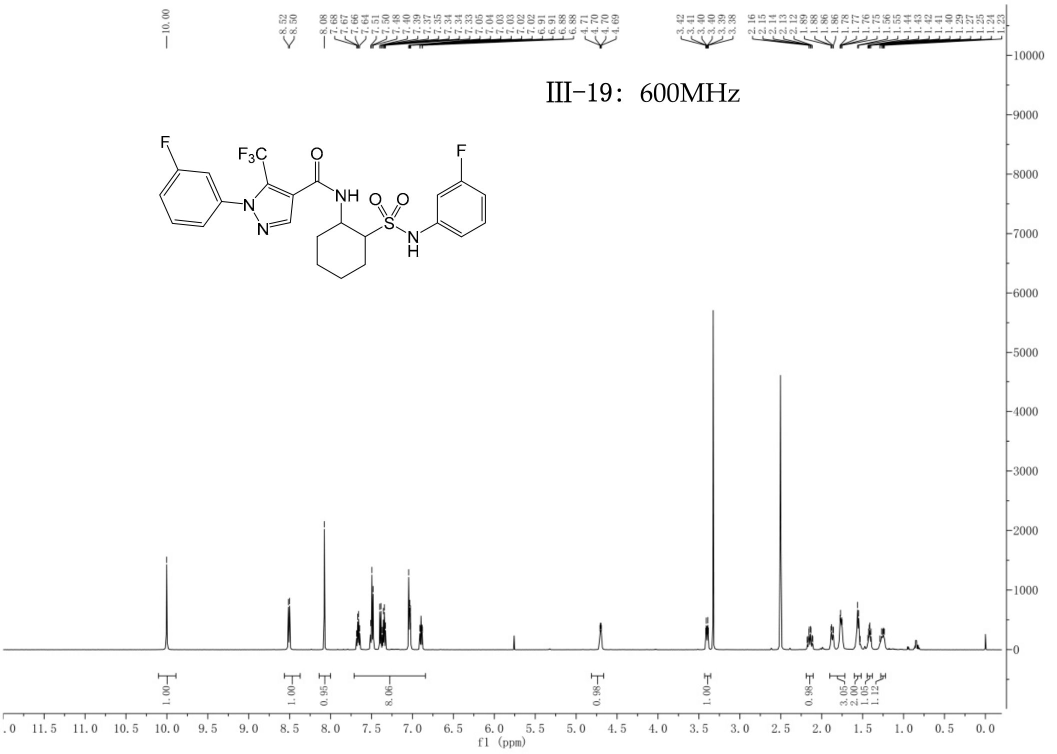

Ⅲ-20：600MHz

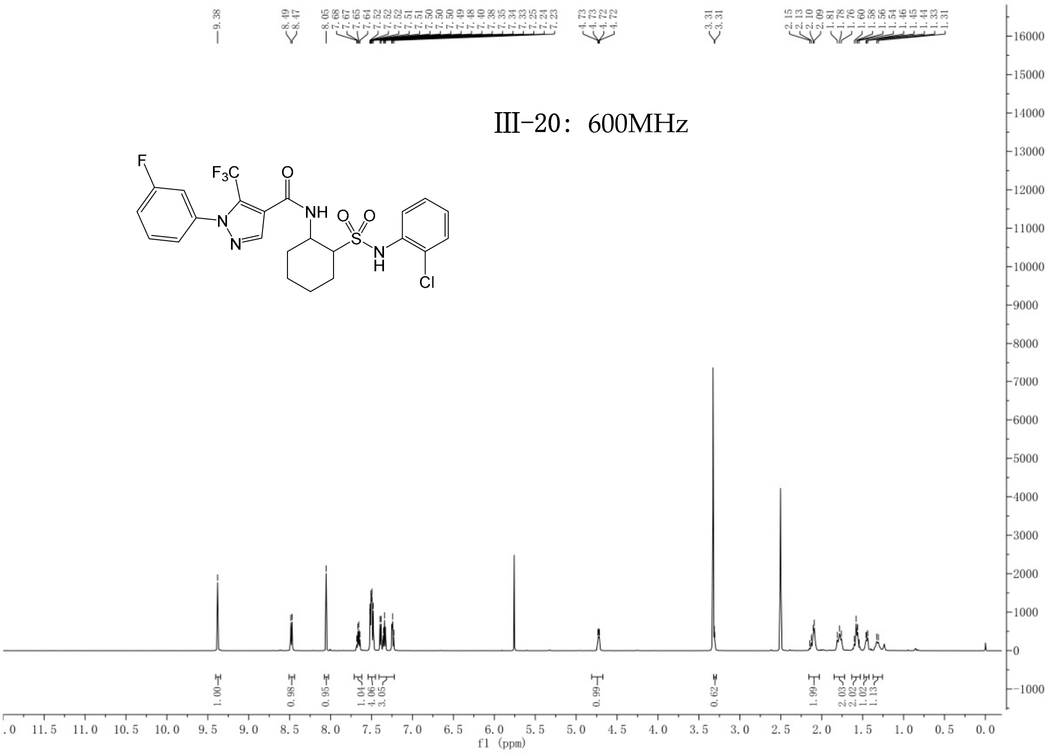

Ⅲ-21：600MHz

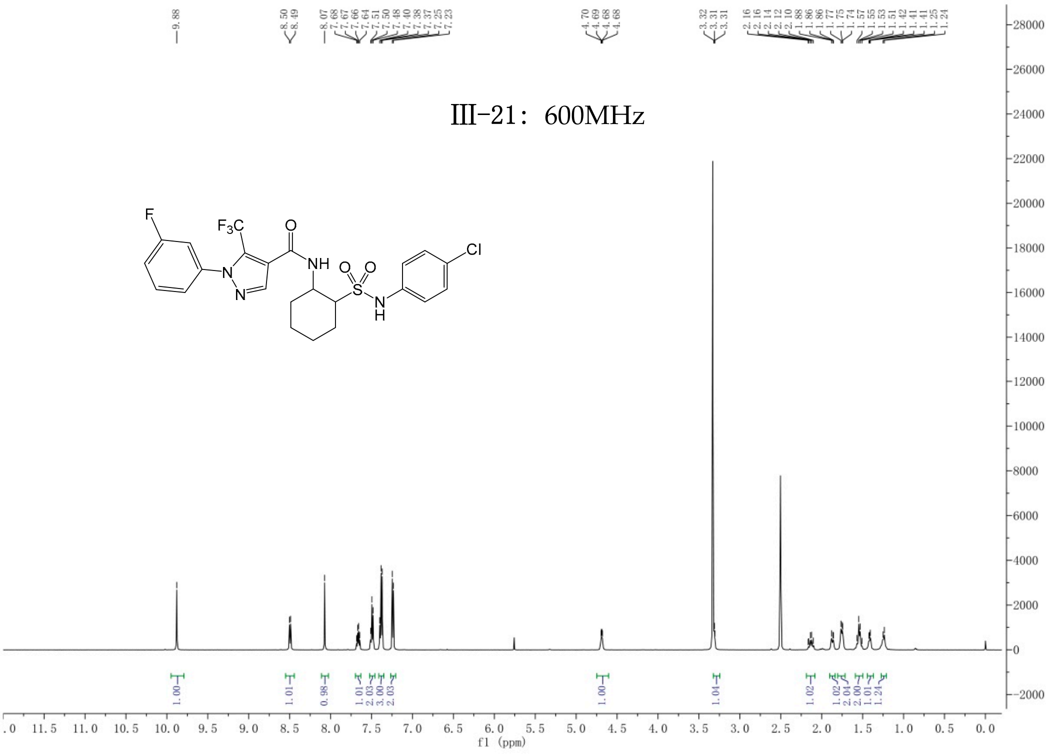

Ⅲ-22：600MHz

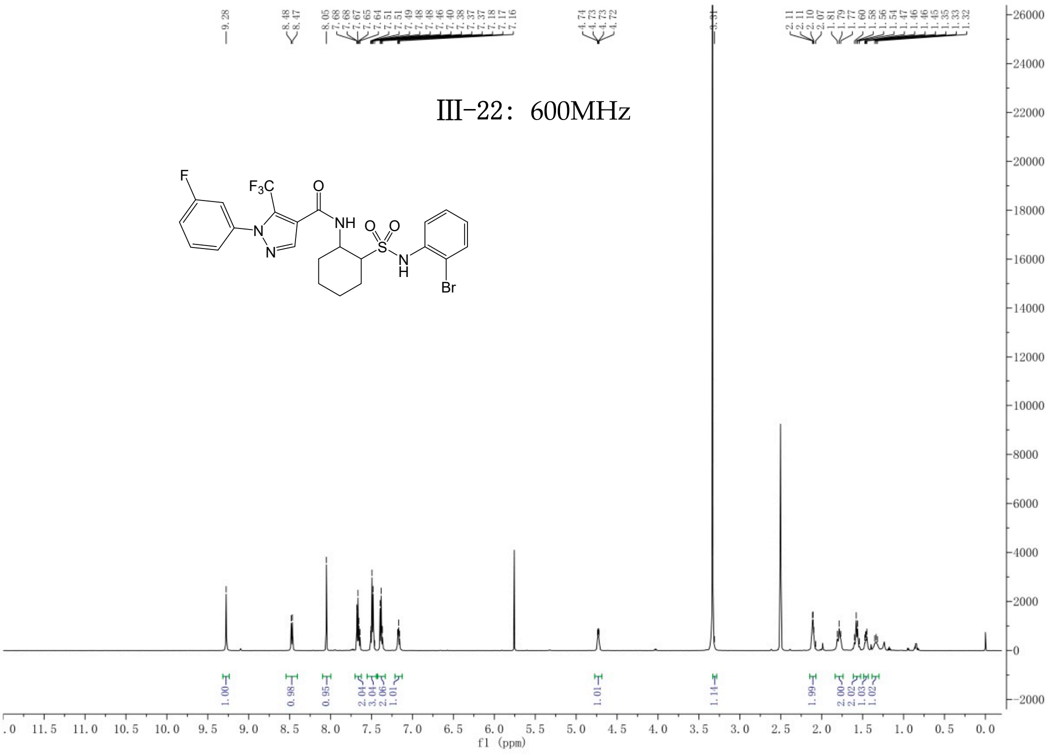

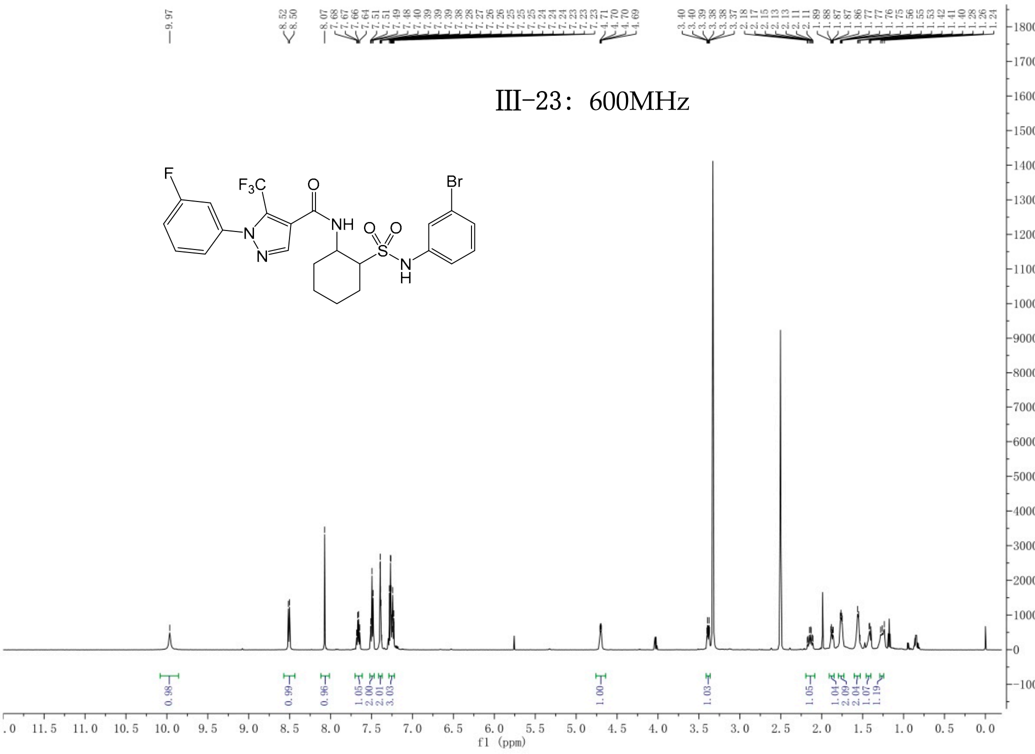
Ⅲ-23：600MHz
F
F_3C
O
NH
N
N
S
O O
N
H
Br
f1 (ppm)

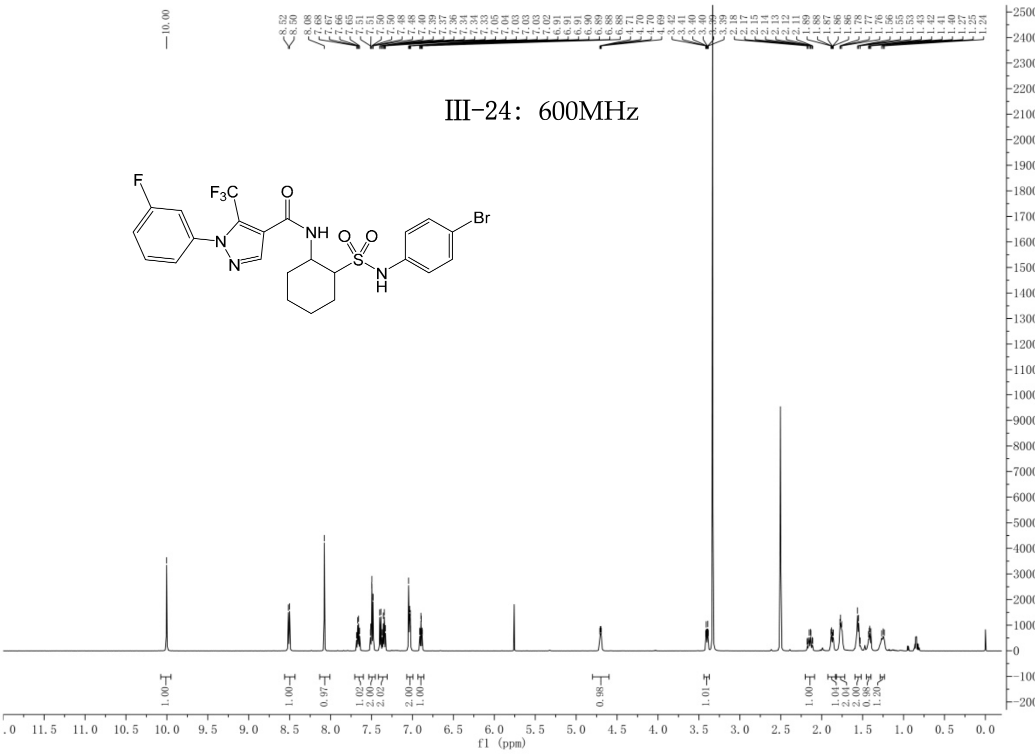
Ⅲ-24：600MHz
F
F_3C
O
NH
N
N
S
O O
N
H
Br
f1 (ppm)

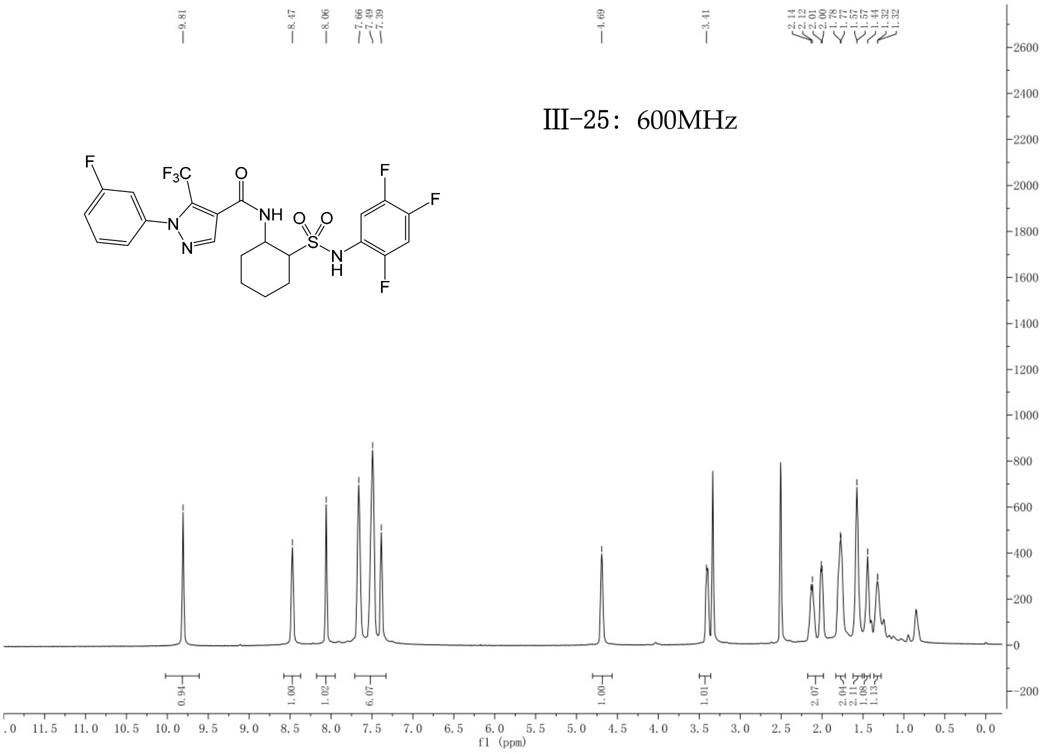
Ⅲ-25：600MHz
9.81
8.47
8.06
7.66
7.49
7.39
4.69
3.41
2.14 2.12 2.01 2.00 1.78 1.77 1.57 1.57 1.44 1.32 1.32
F
F_3C
O
NH
O
O
S
N
H
N
N
F
F
F
f1 (ppm)

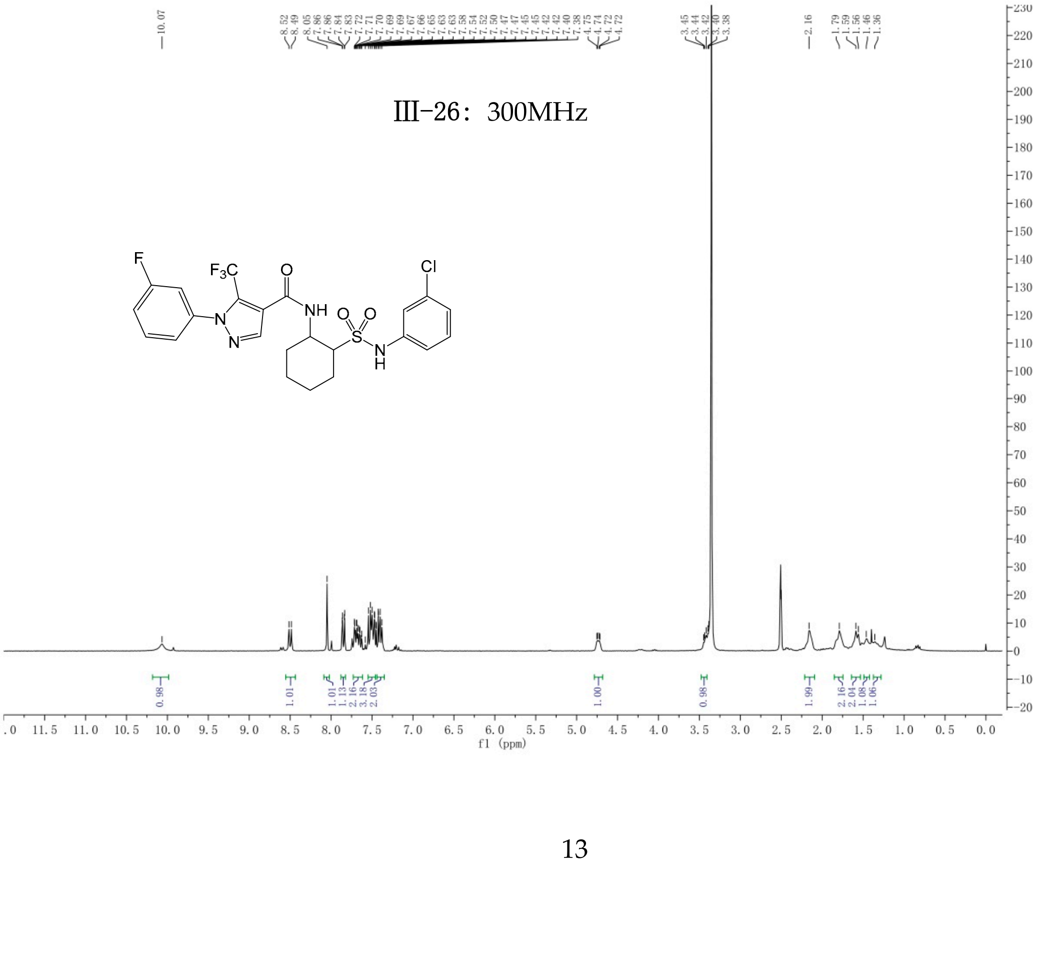
Ⅲ-26：300MHz
F
F_3C
O
NH
O
O
S
N
H
N
N
Cl
f1 (ppm)

III-27：600MHz

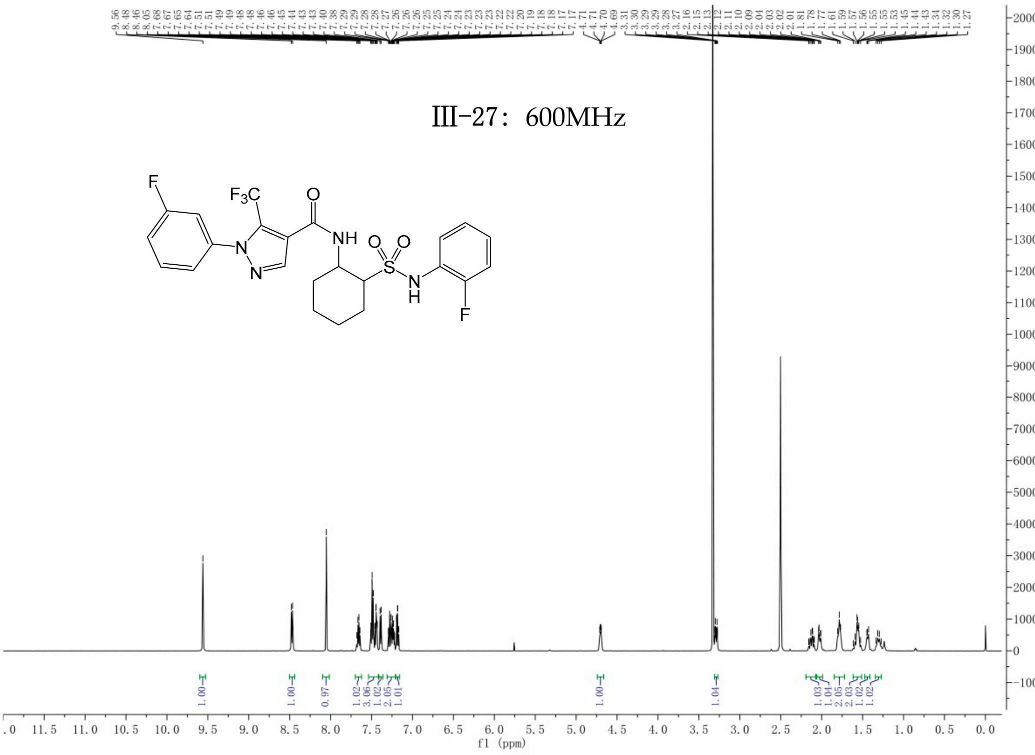

III-28：600MHz

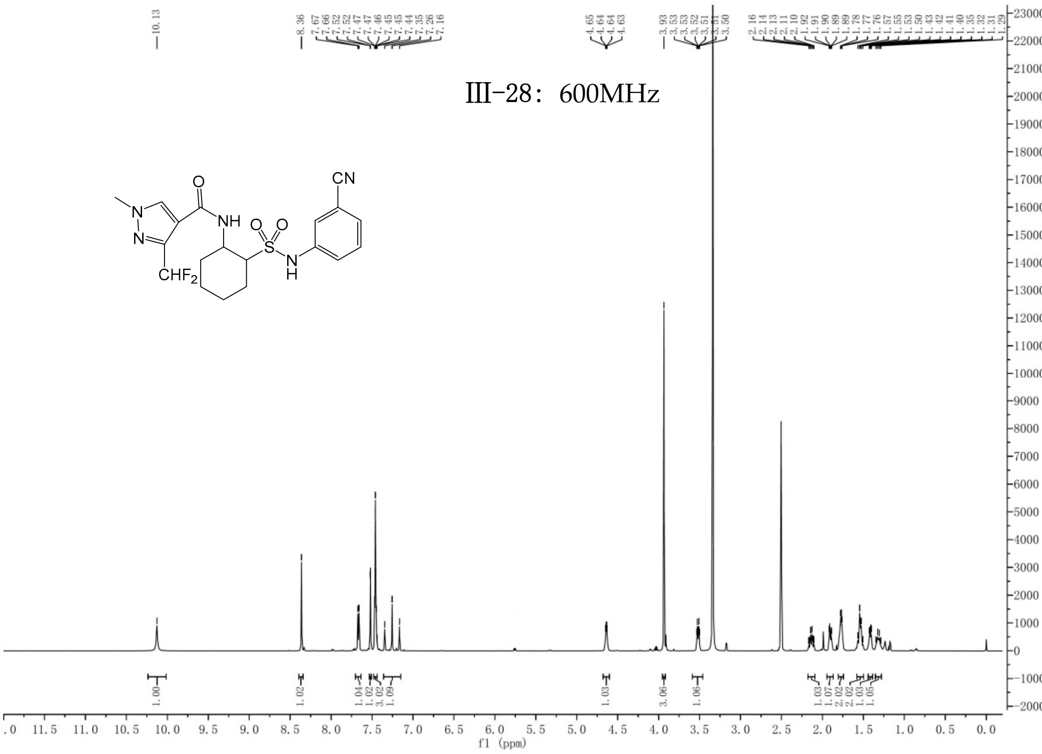

III-29：600MHz

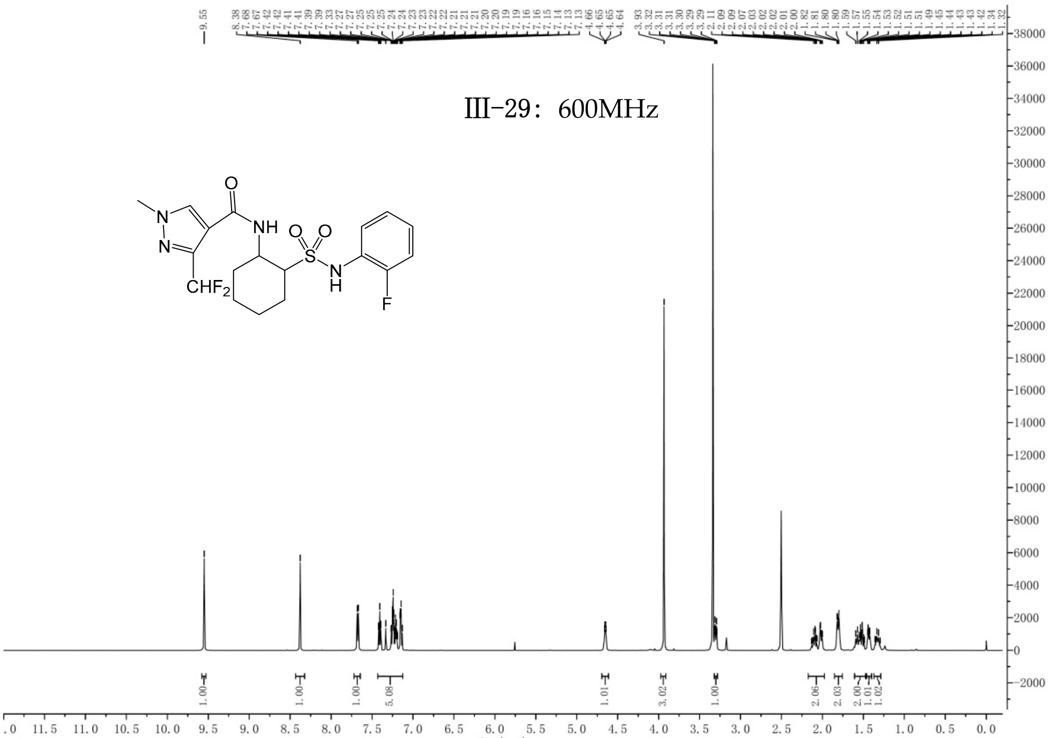

III-30：600MHz

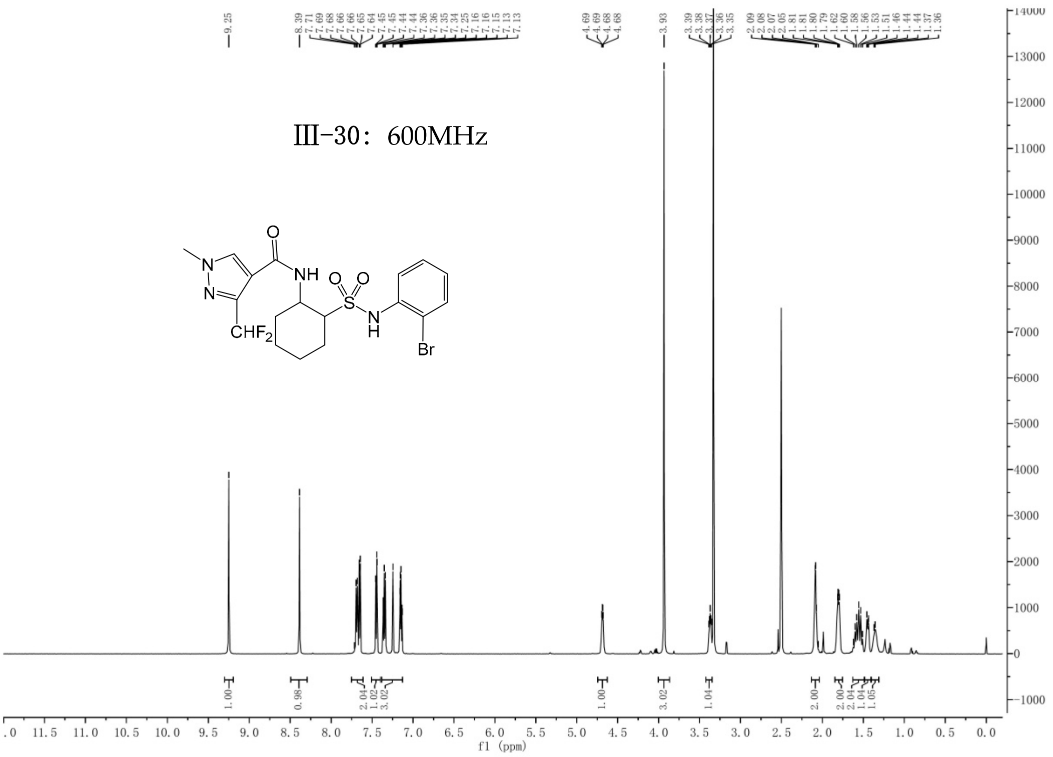

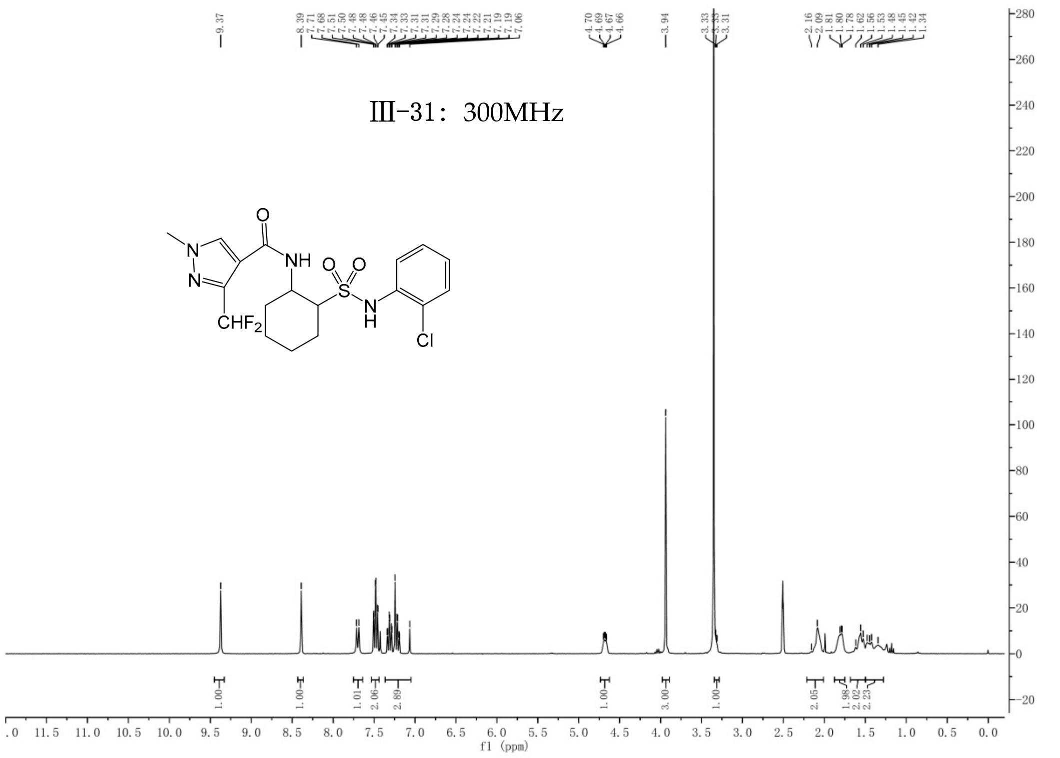
Ⅲ-31：300MHz
CHF2
Cl
f1 (ppm)

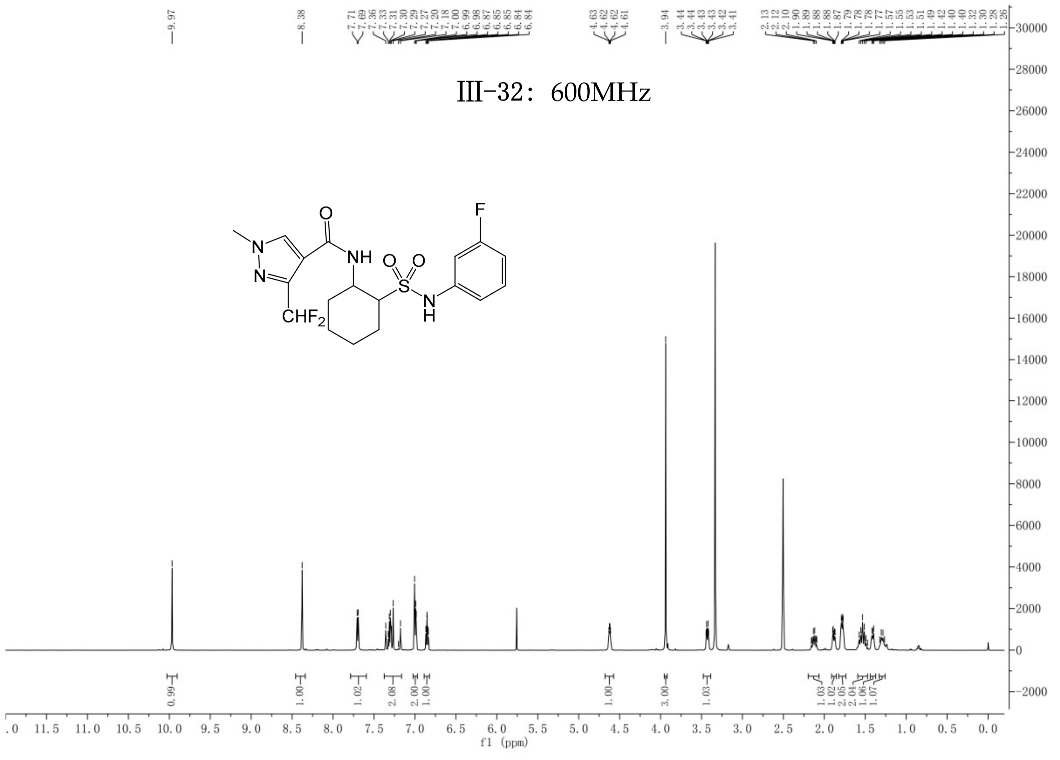
Ⅲ-32：600MHz
F
CHF2
f1 (ppm)

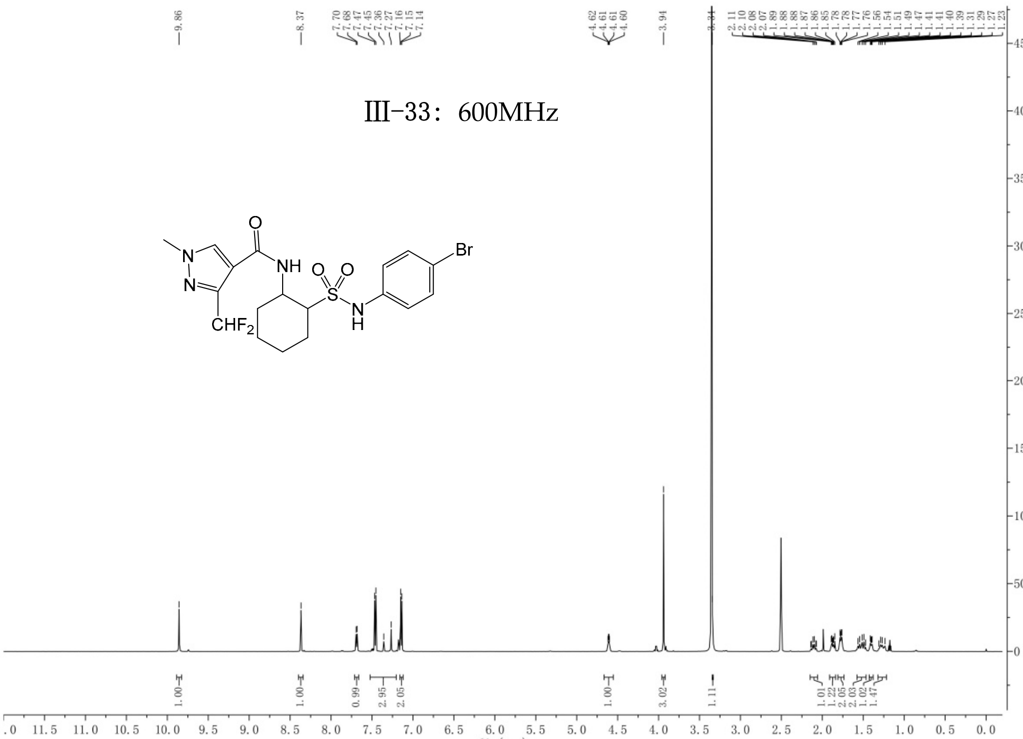
Ⅲ-33：600MHz
f1 (ppm)

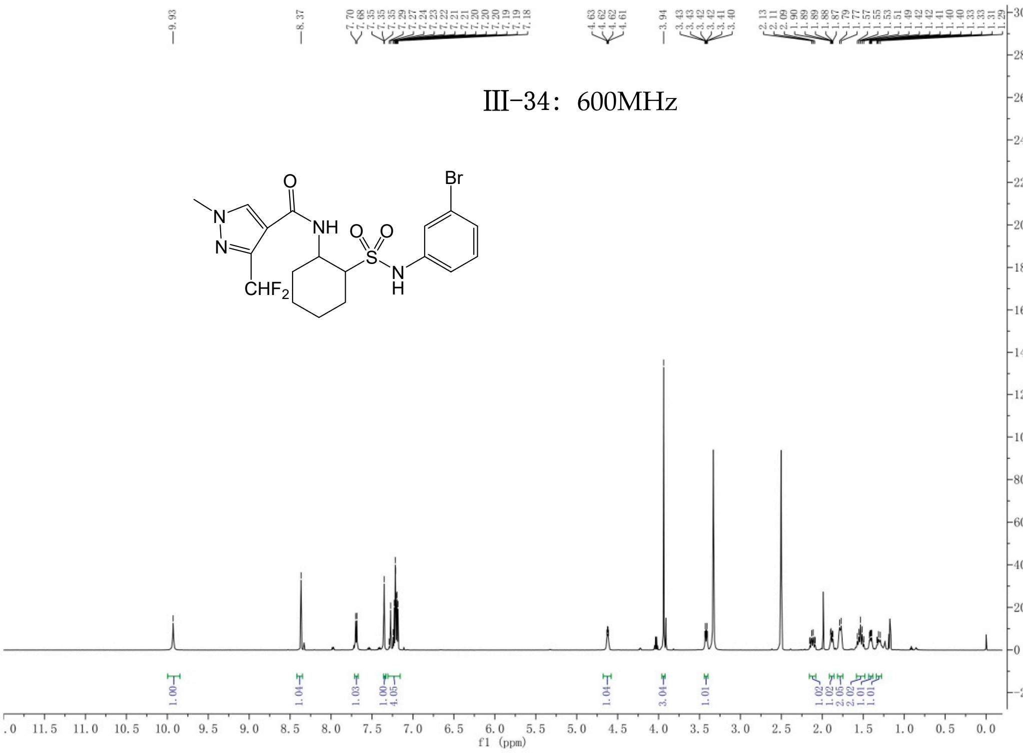
Ⅲ-34：600MHz
f1 (ppm)

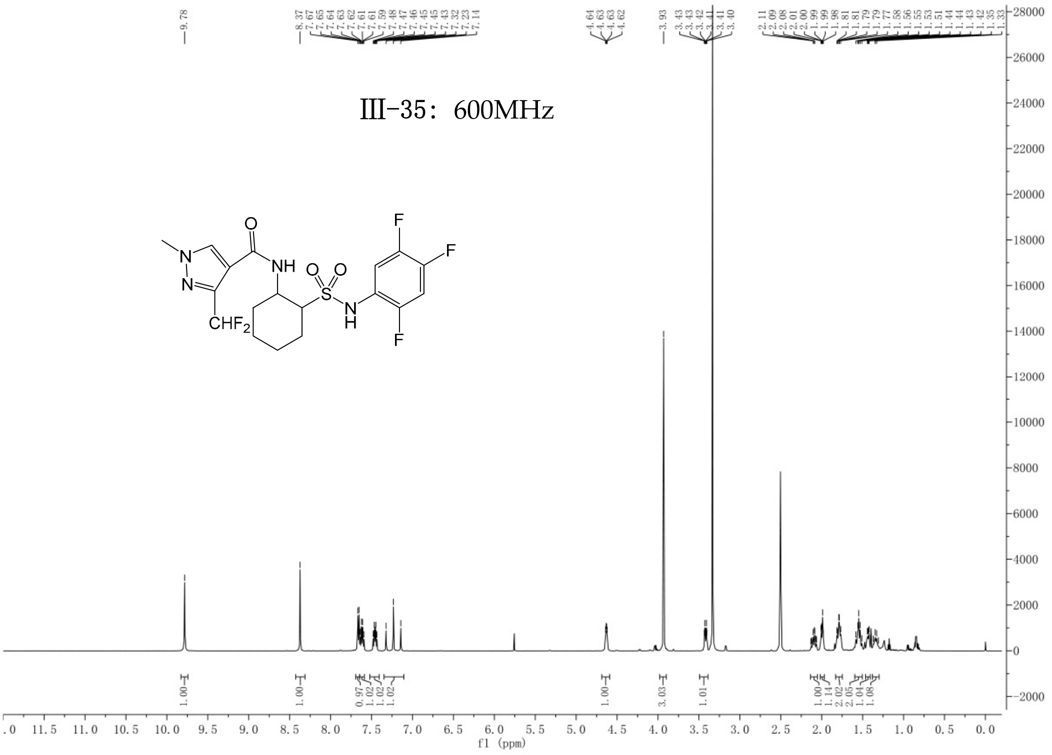
Ⅲ-35：600MHz
f1 (ppm)

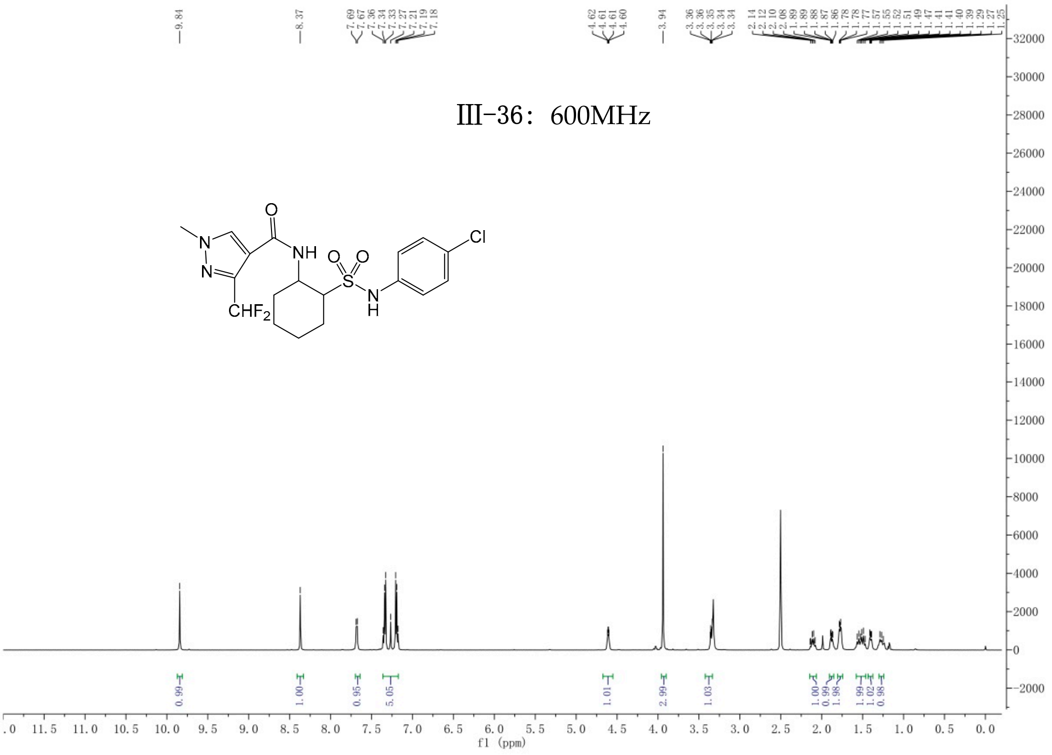
Ⅲ-36：600MHz
f1 (ppm)

All ^{13}C-NMR spectra used dimethyl sulfoxide (DMSO-d_6) as solvent. Solvent peak was at 40ppm.

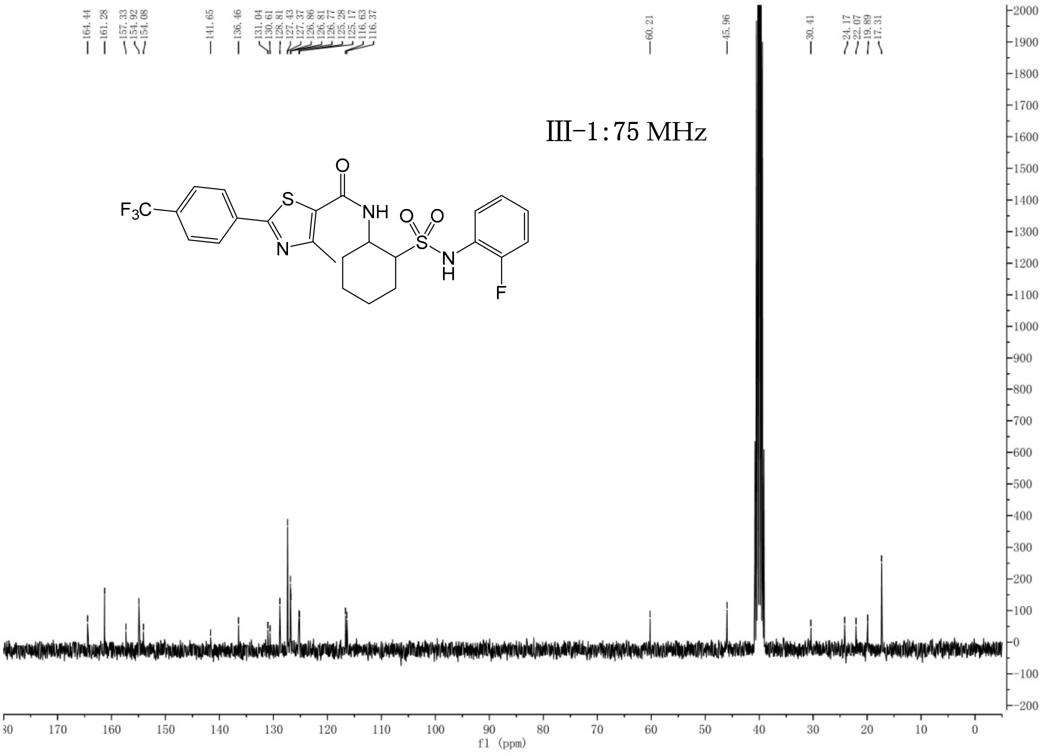

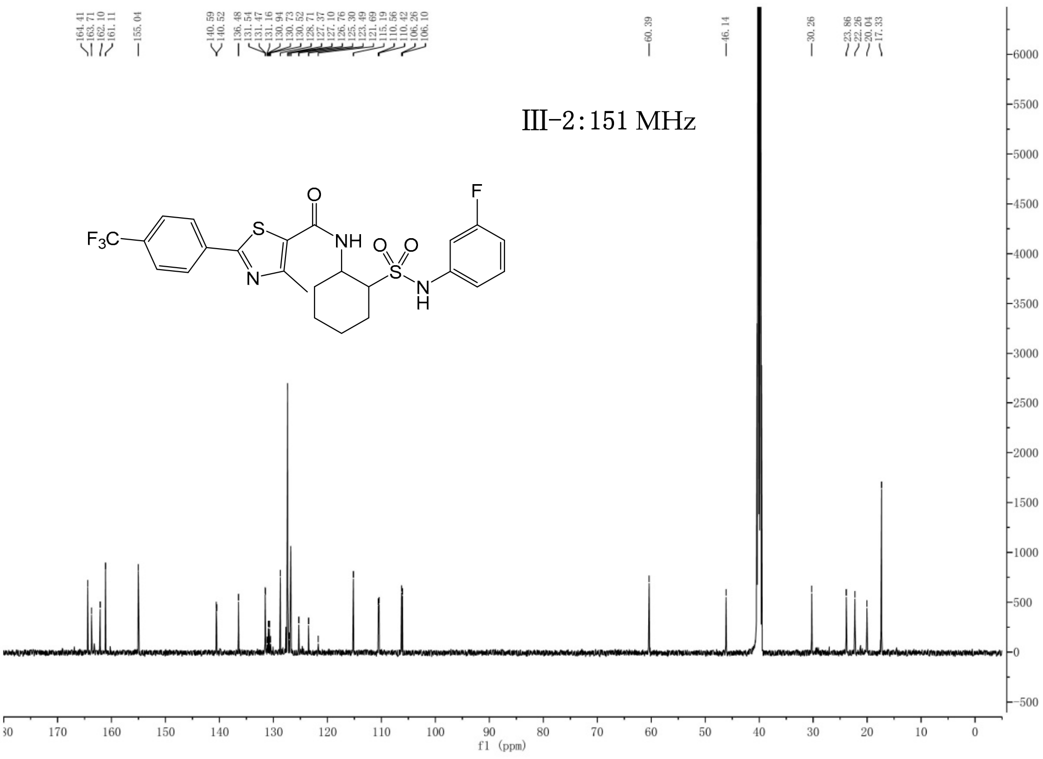

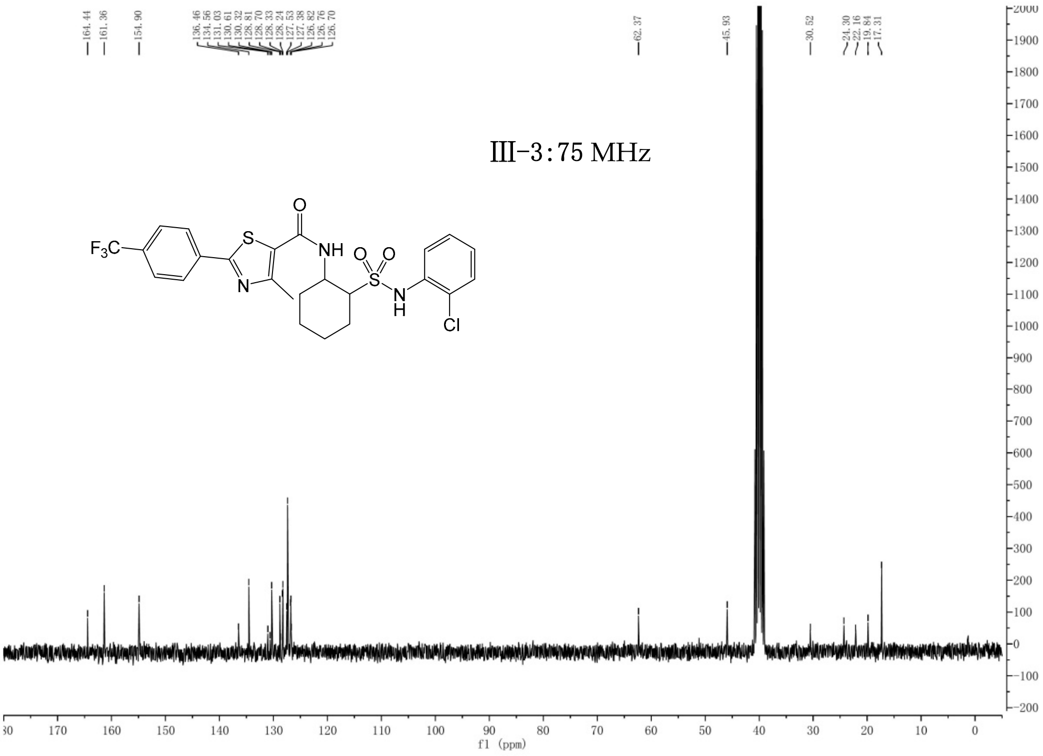

Ⅲ-3:75 MHz
F3C
S
N
O
NH
O
S
O
N
H
Cl
f1 (ppm)

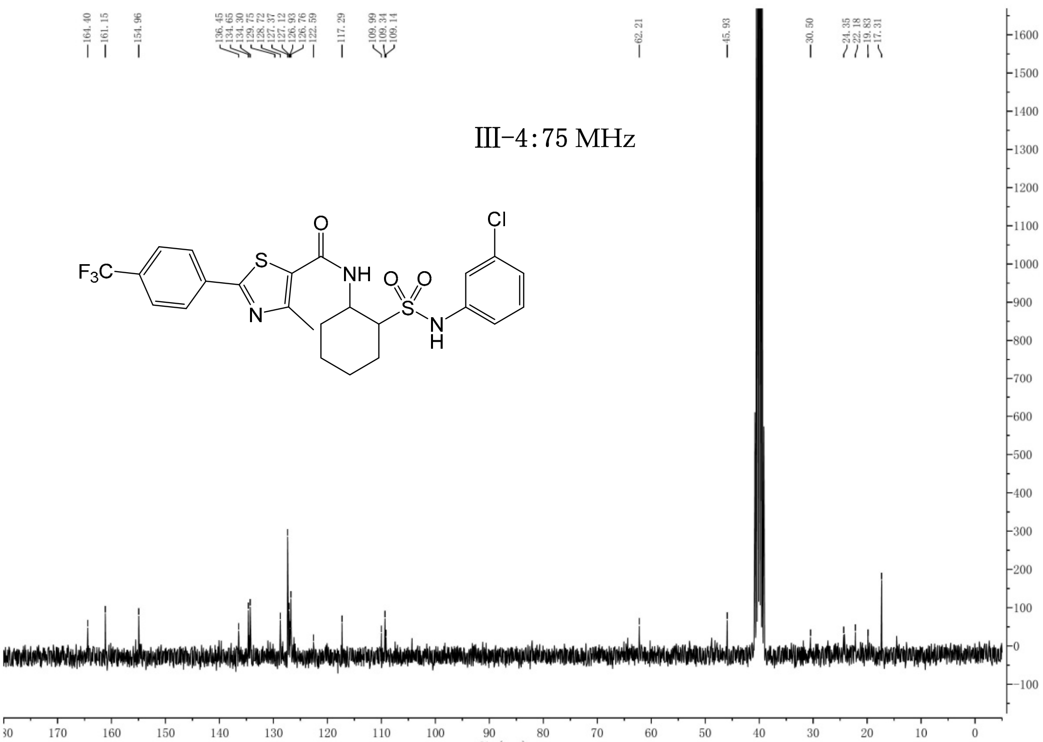

Ⅲ-4:75 MHz
F3C
S
N
O
NH
O
S
O
N
H
Cl
f1 (ppm)

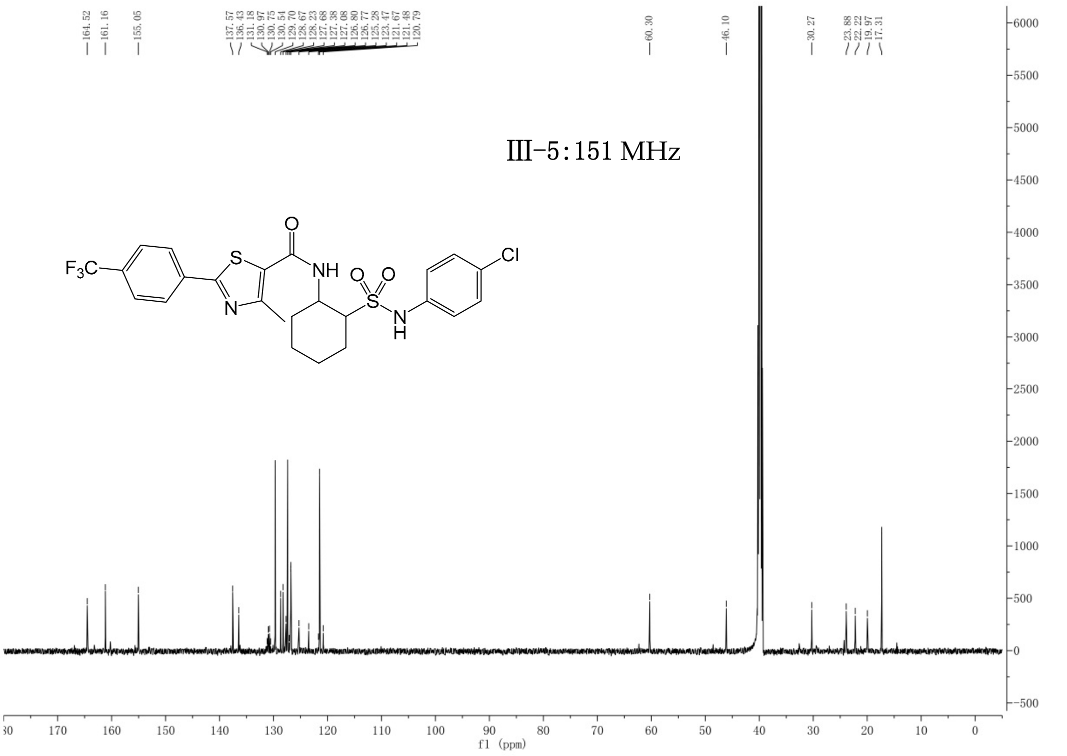
III-5:151 MHz
F3C
S
N
O
NH
O
O
S
N
H
Cl
f1 (ppm)

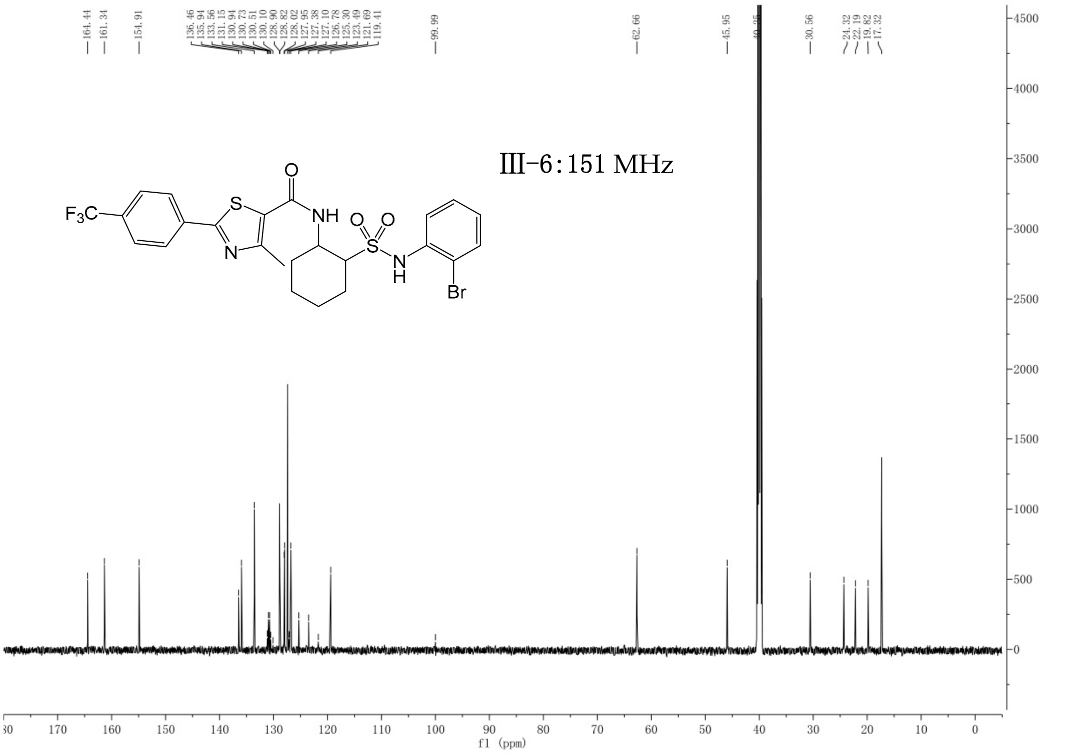
III-6:151 MHz
F3C
S
N
O
NH
O
O
S
N
H
Br
f1 (ppm)

Ⅲ-7: 151 MHz

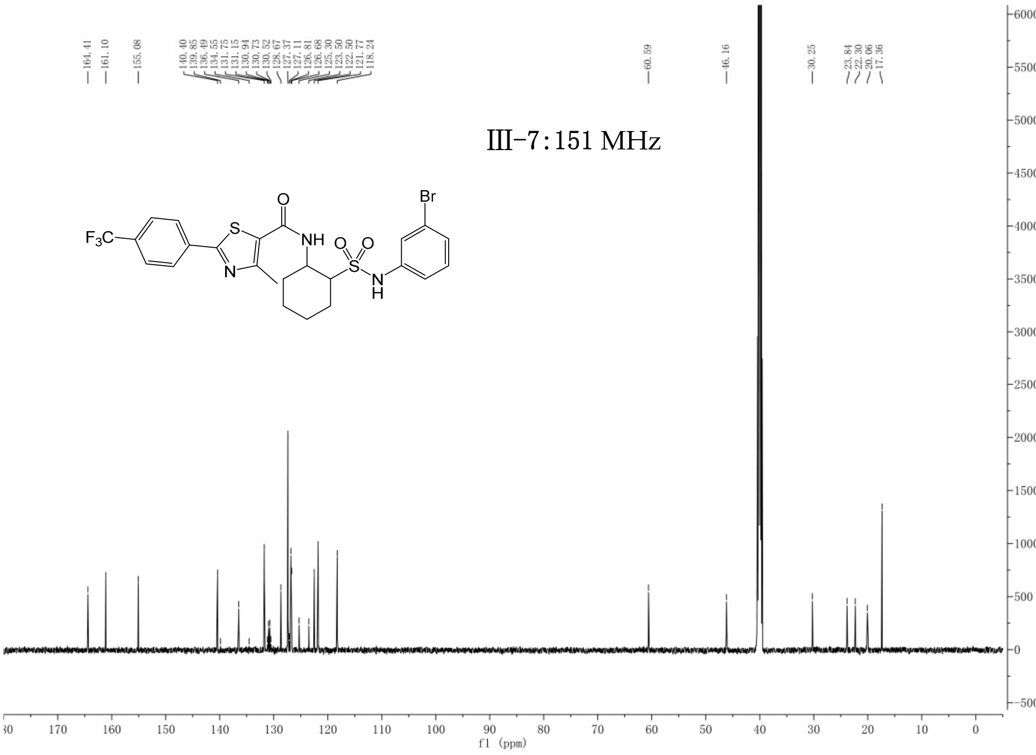

Ⅲ-8: 151 MHz

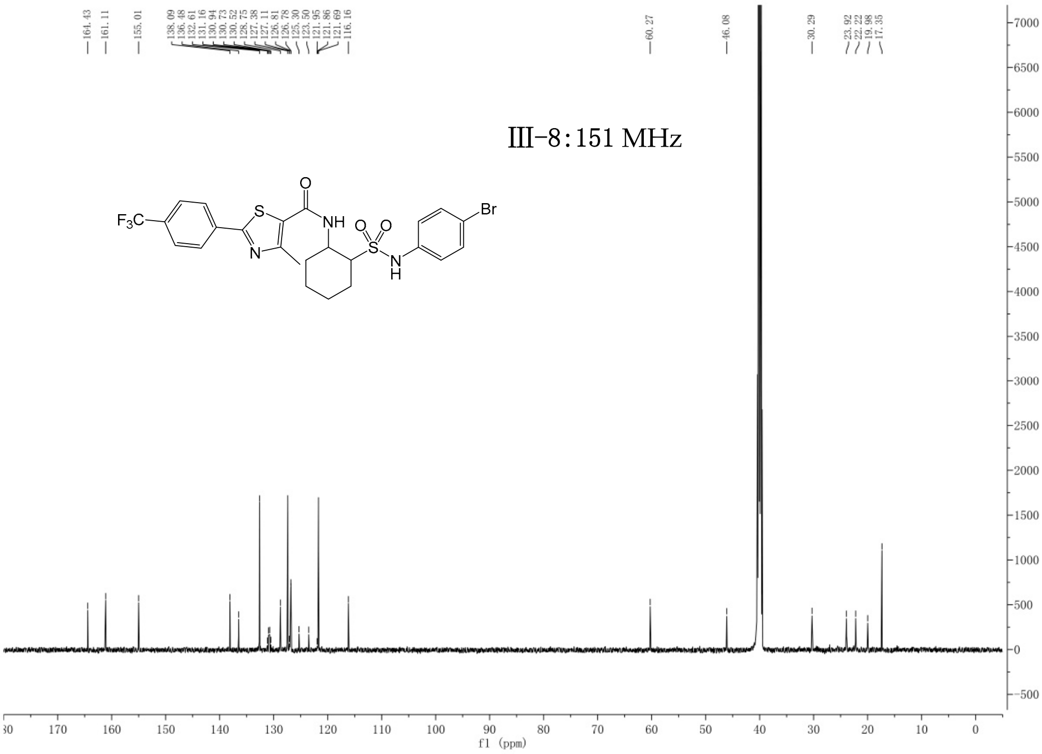

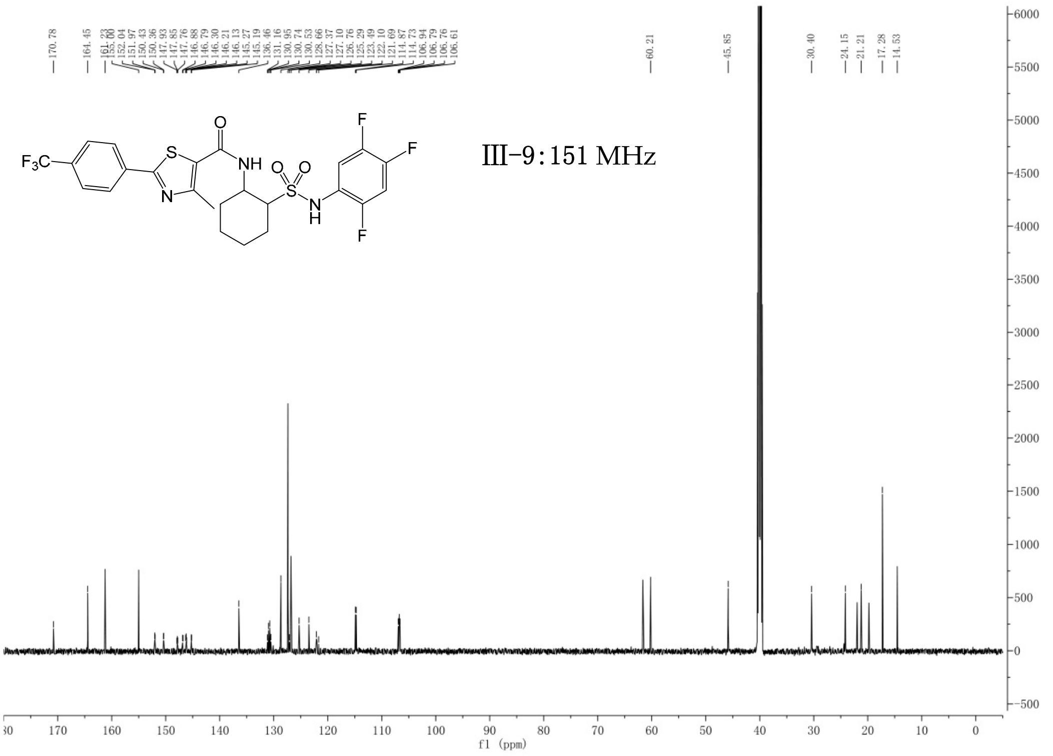
Ⅲ-9：151 MHz
f1 (ppm)

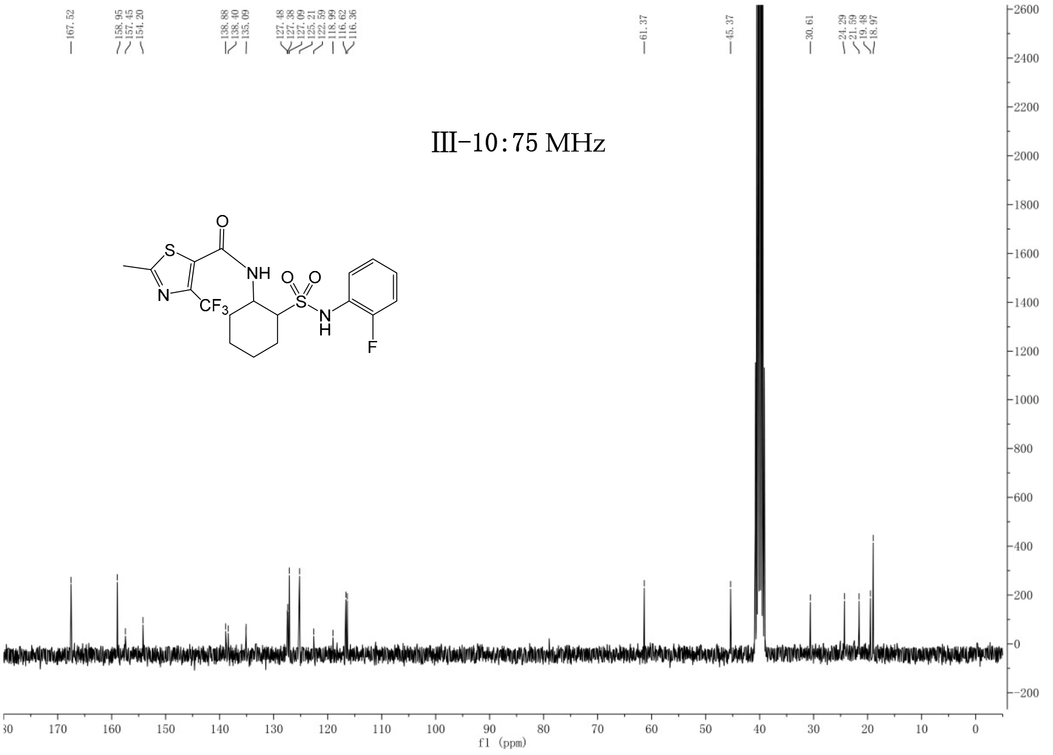
Ⅲ-10：75 MHz
f1 (ppm)

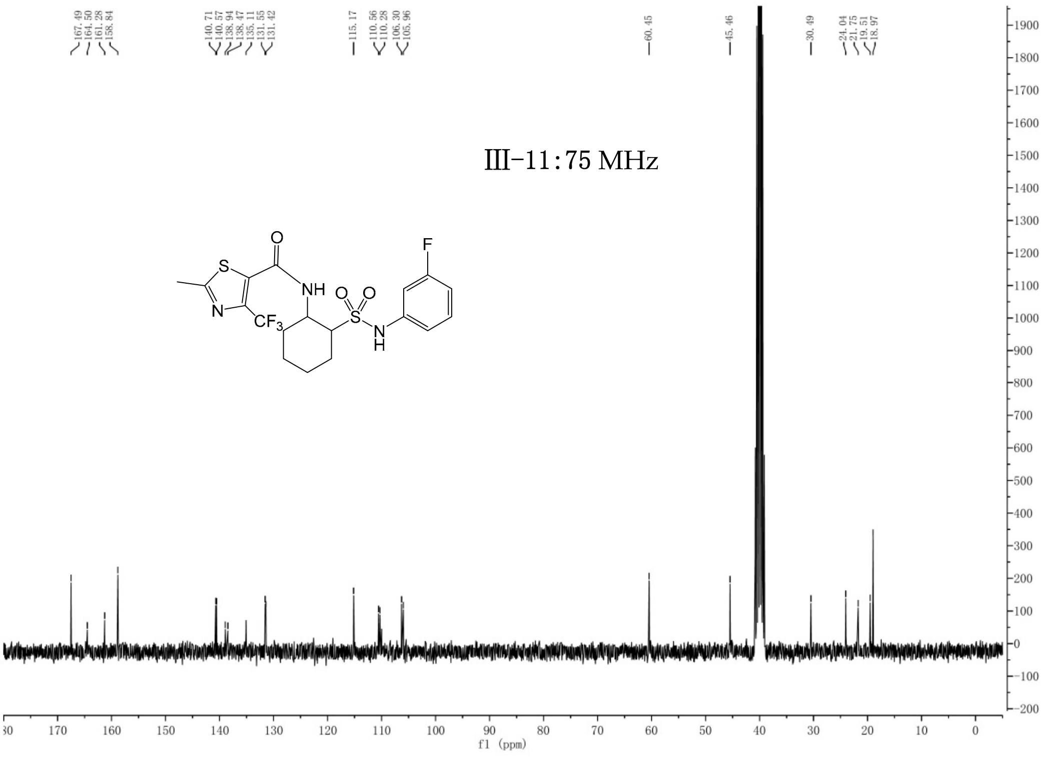
III-11:75 MHz
f1 (ppm)

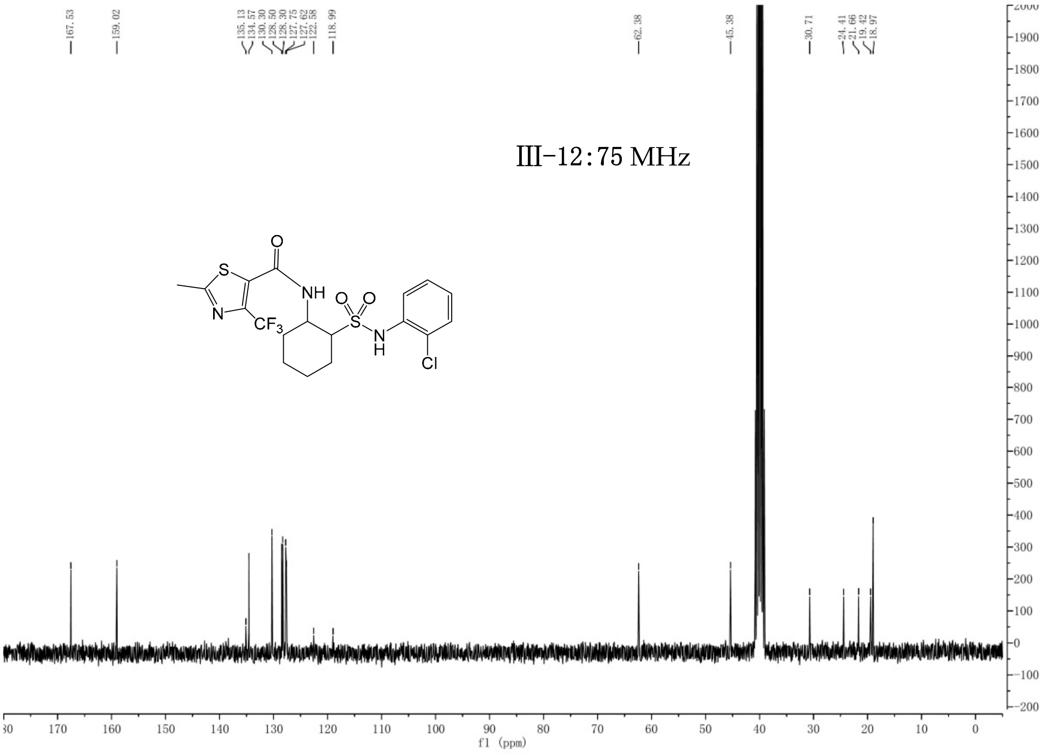
III-12:75 MHz
f1 (ppm)

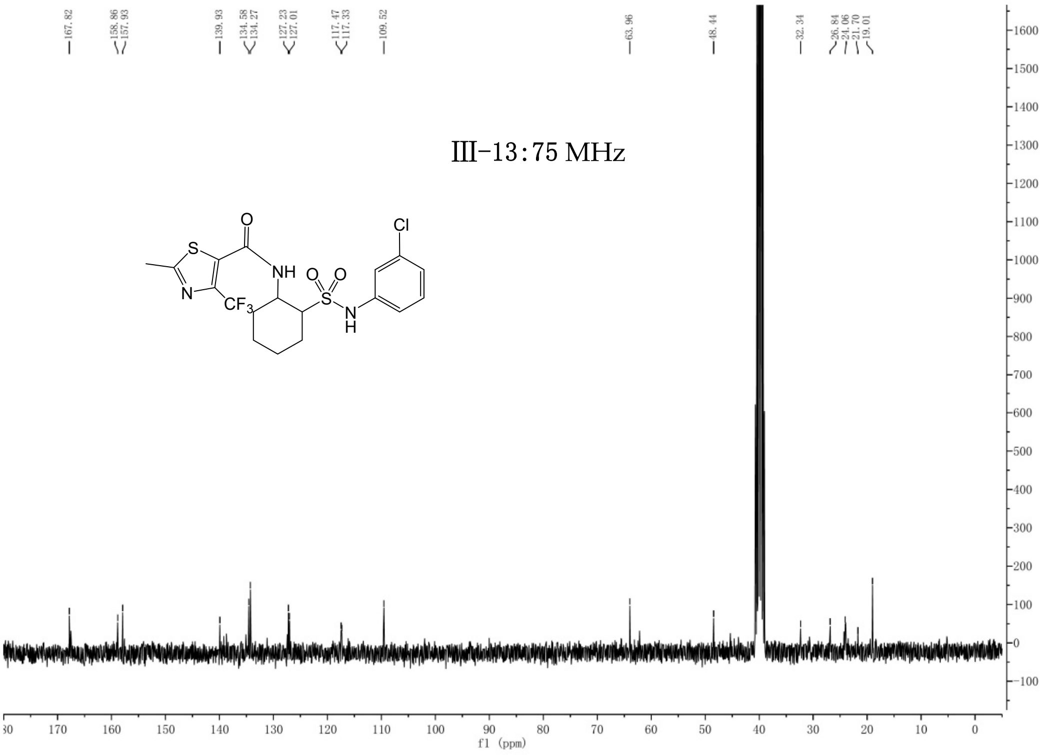
167.82
158.86
157.93
139.93
134.58
134.27
127.23
127.01
117.47
117.33
109.52
63.96
48.44
32.34
26.84
24.06
21.70
19.01
Ⅲ-13:75 MHz
f1 (ppm)

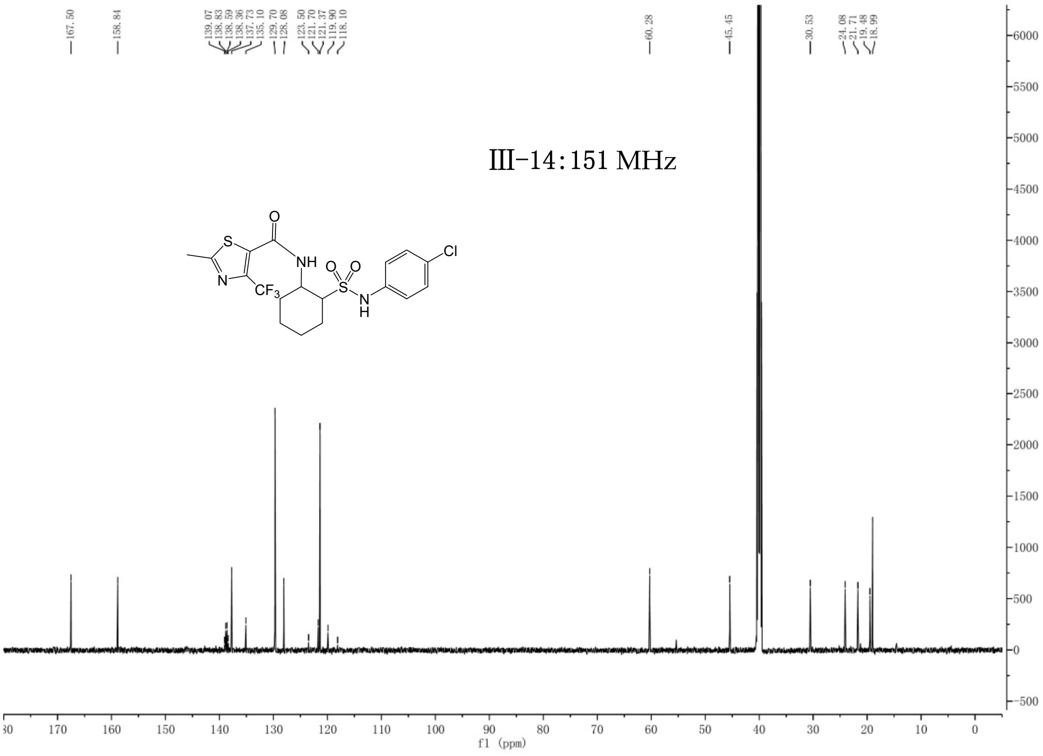
167.50
158.84
139.07
138.83
138.59
138.36
137.73
135.10
129.70
128.08
123.50
121.70
121.37
119.90
118.10
60.28
45.45
30.53
24.08
21.71
19.48
18.99
Ⅲ-14:151 MHz
f1 (ppm)

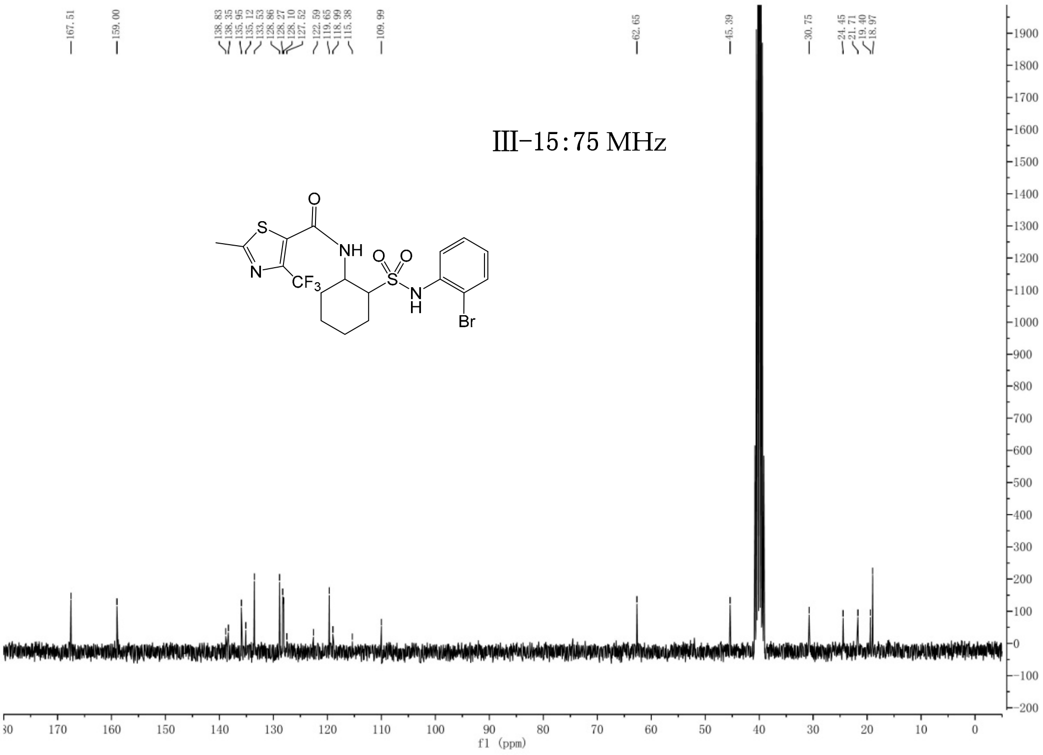
167.51
159.00
138.83
138.35
135.95
135.12
133.53
128.86
128.27
128.10
127.52
122.59
119.65
118.99
115.38
109.99
62.65
45.39
30.75
24.45
21.71
19.40
18.97
Ⅲ-15:75 MHz
O
S
NH
N
CF3
O
O
S
N
H
Br
f1 (ppm)

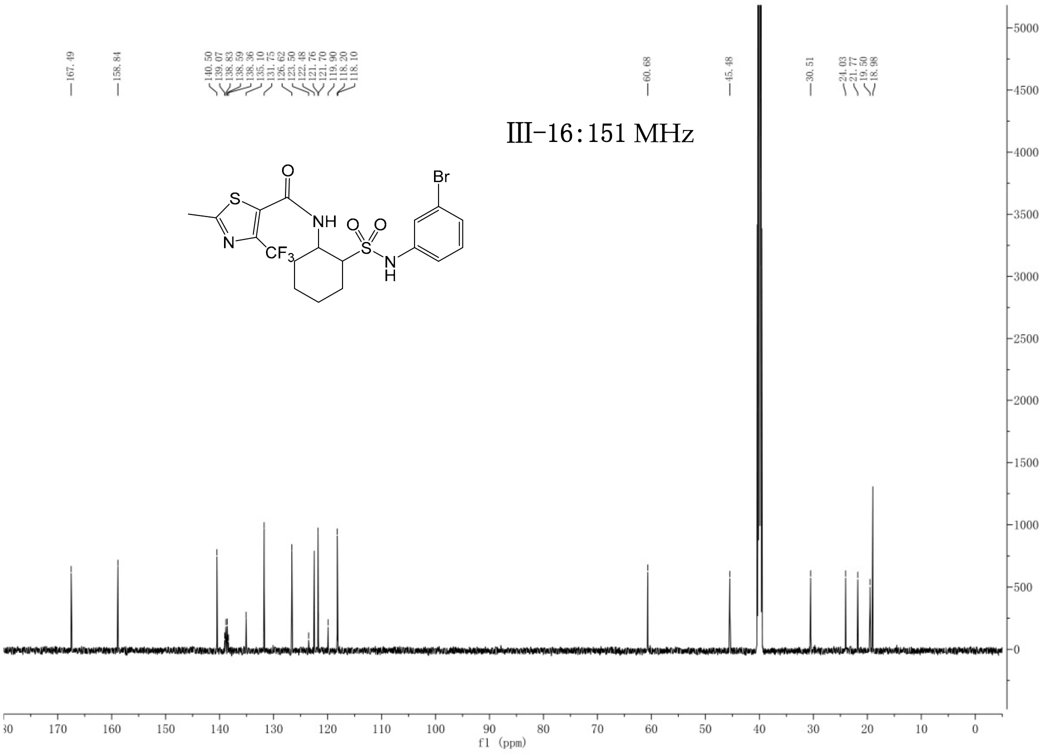
167.49
158.84
140.50
139.07
138.83
138.59
138.36
135.10
131.75
126.62
123.50
122.48
121.76
121.70
119.90
118.20
118.10
60.68
45.48
30.51
24.03
21.77
19.50
18.98
Ⅲ-16:151 MHz
O
S
NH
N
CF3
O
O
S
N
H
Br
f1 (ppm)

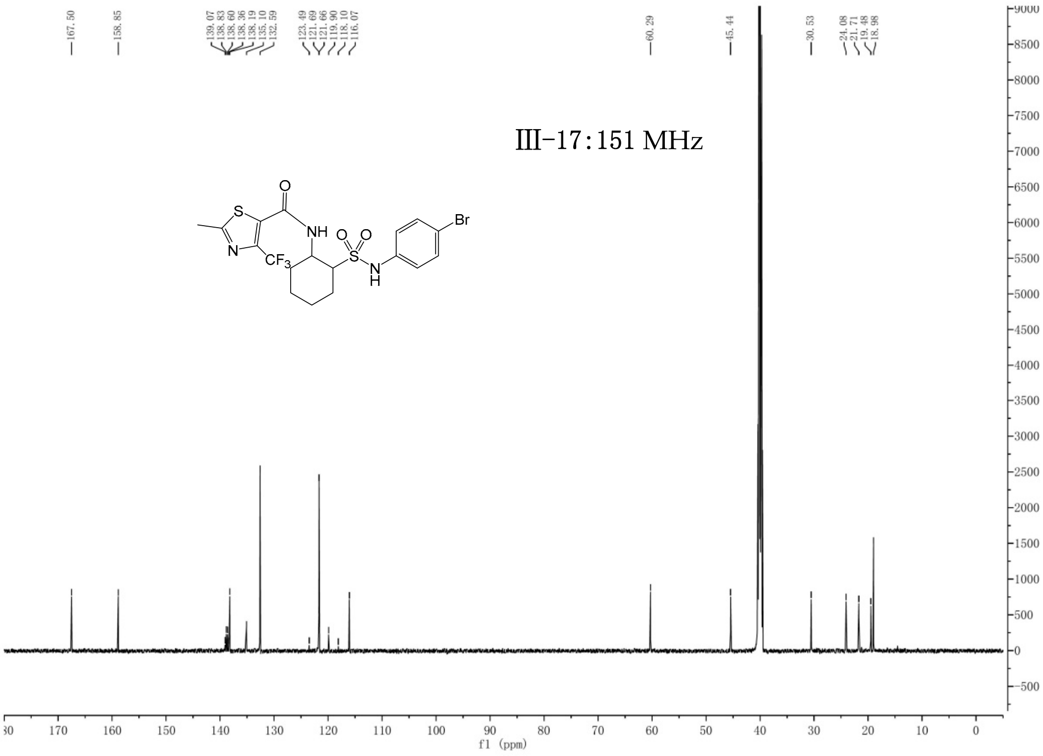
Ⅲ-17：151 MHz
f1 (ppm)

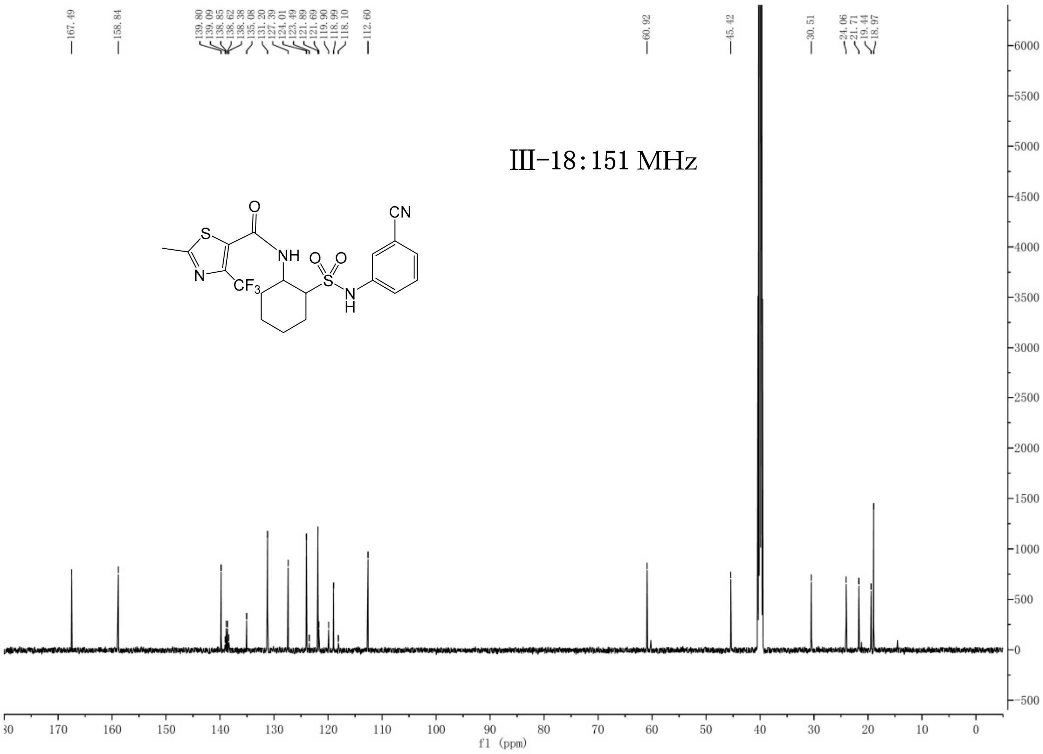
Ⅲ-18：151 MHz
f1 (ppm)

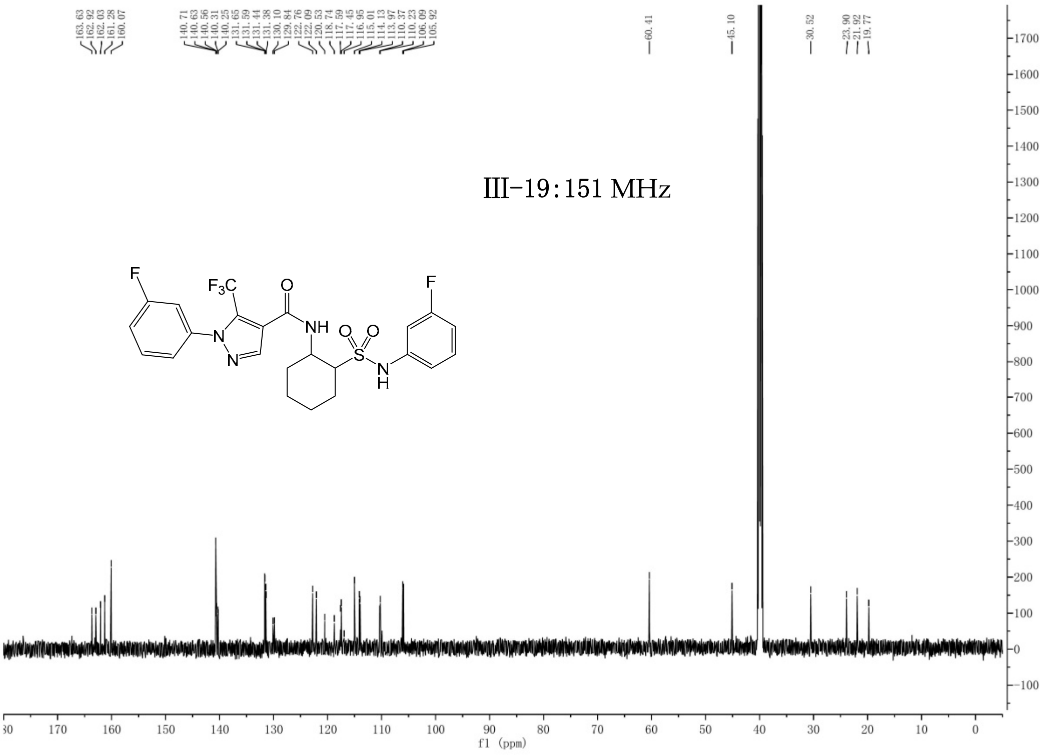

Ⅲ-19:151 MHz

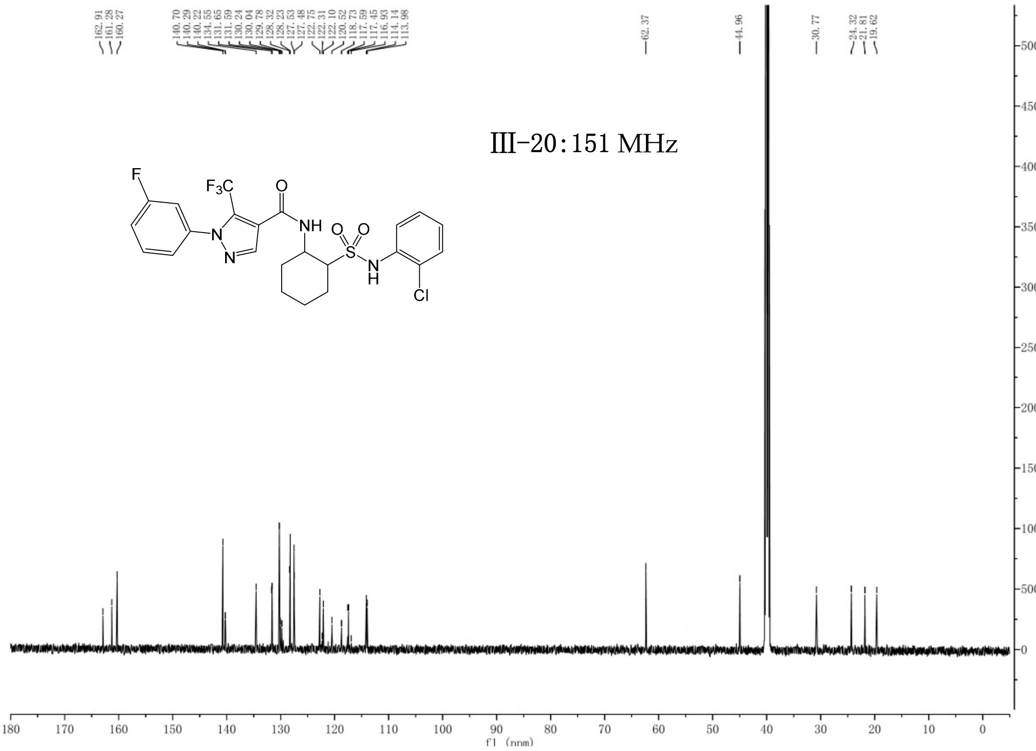

Ⅲ-20:151 MHz

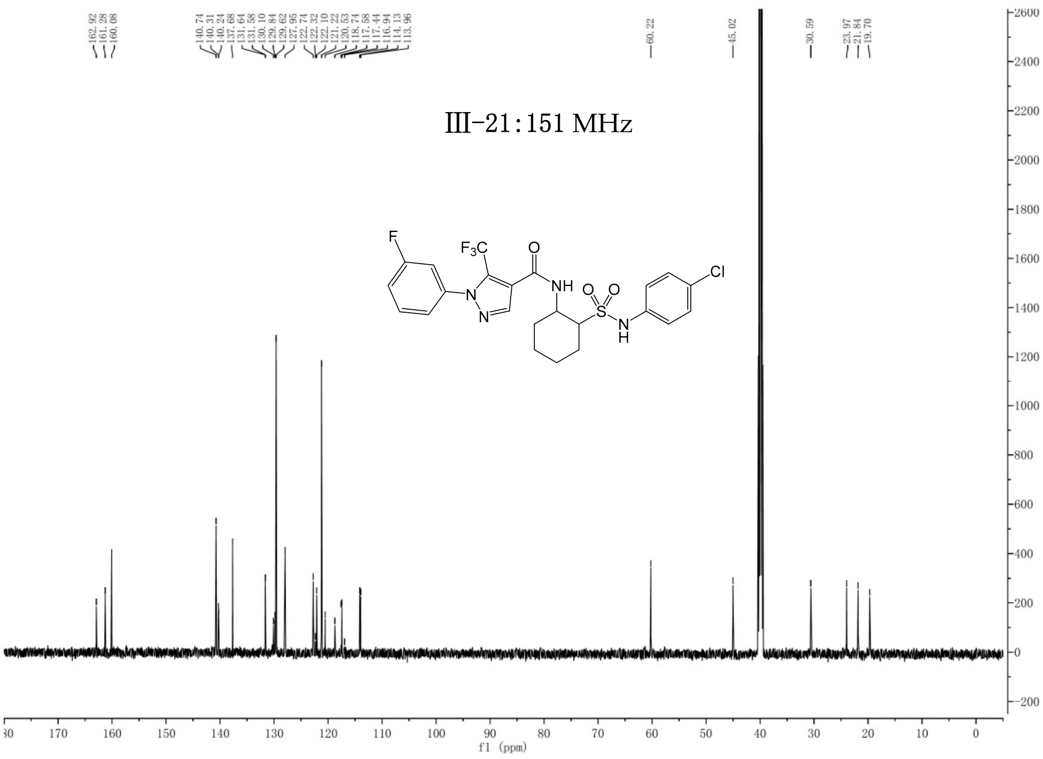
Ⅲ-21：151 MHz
162.92
161.28
160.08
140.74
140.31
140.24
137.68
131.64
131.58
130.10
129.84
129.62
127.95
122.74
122.32
122.10
121.22
120.53
118.74
117.58
117.44
116.94
114.13
113.96
60.22
45.02
30.59
23.97
21.84
19.70
F
F_3C
O
NH
N
N
O
S
O
N
H
Cl
f1 (ppm)

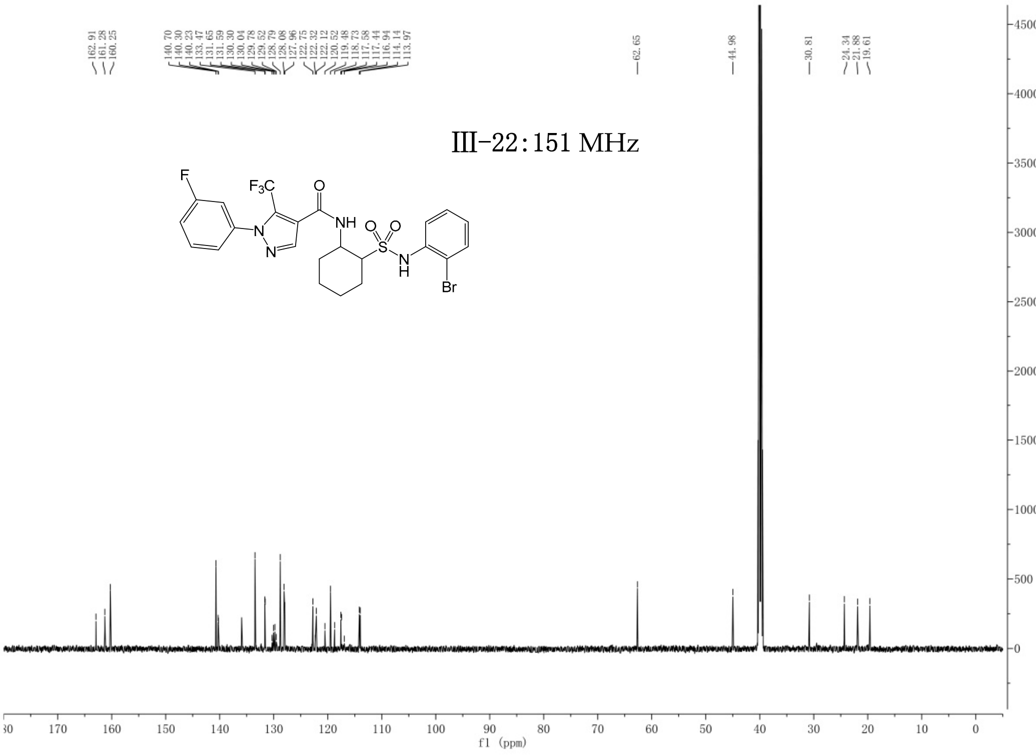
Ⅲ-22：151 MHz
162.91
161.28
160.25
140.70
140.30
140.23
133.47
131.65
131.59
130.30
130.04
129.78
129.52
128.79
128.08
127.96
122.75
122.33
122.12
120.52
119.48
118.73
117.58
117.44
116.94
114.14
113.97
62.65
44.98
30.81
24.34
21.88
19.61
F
F_3C
O
NH
N
N
O
S
O
N
H
Br
f1 (ppm)

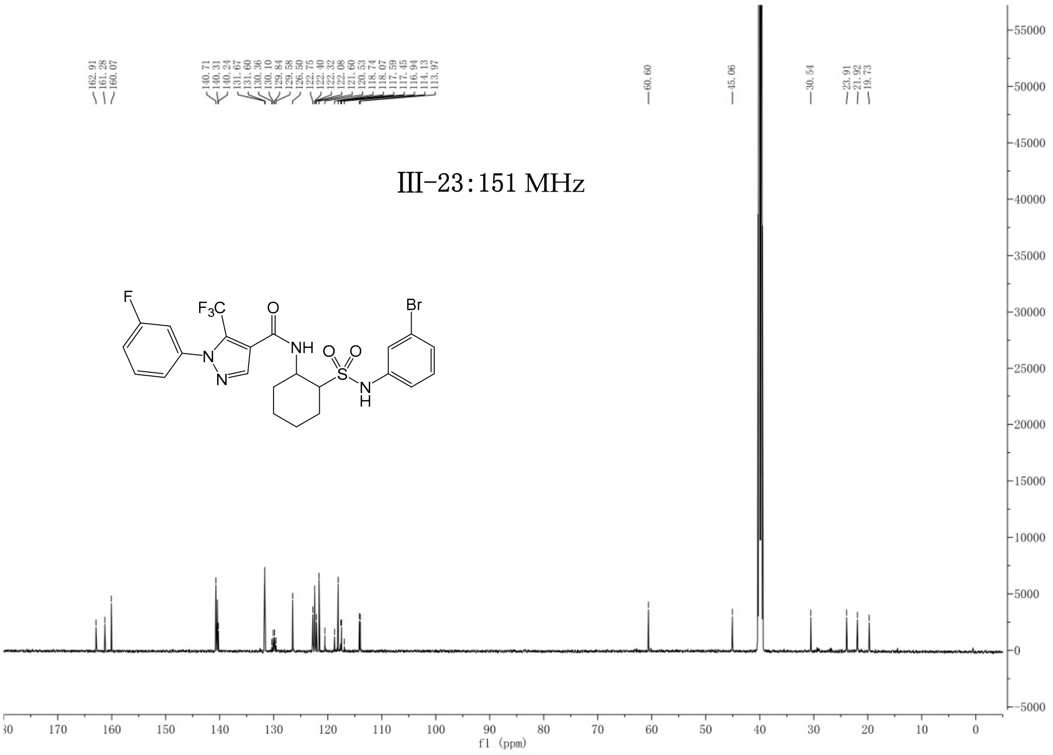
Ⅲ-23:151 MHz
162.91
161.28
160.07
140.71
140.31
140.24
131.67
131.60
130.36
130.10
129.84
129.58
126.50
122.75
122.40
122.32
122.08
121.60
120.53
118.74
118.07
117.59
117.45
116.94
114.13
113.97
60.60
45.06
30.54
23.91
21.92
19.73
f1 (ppm)

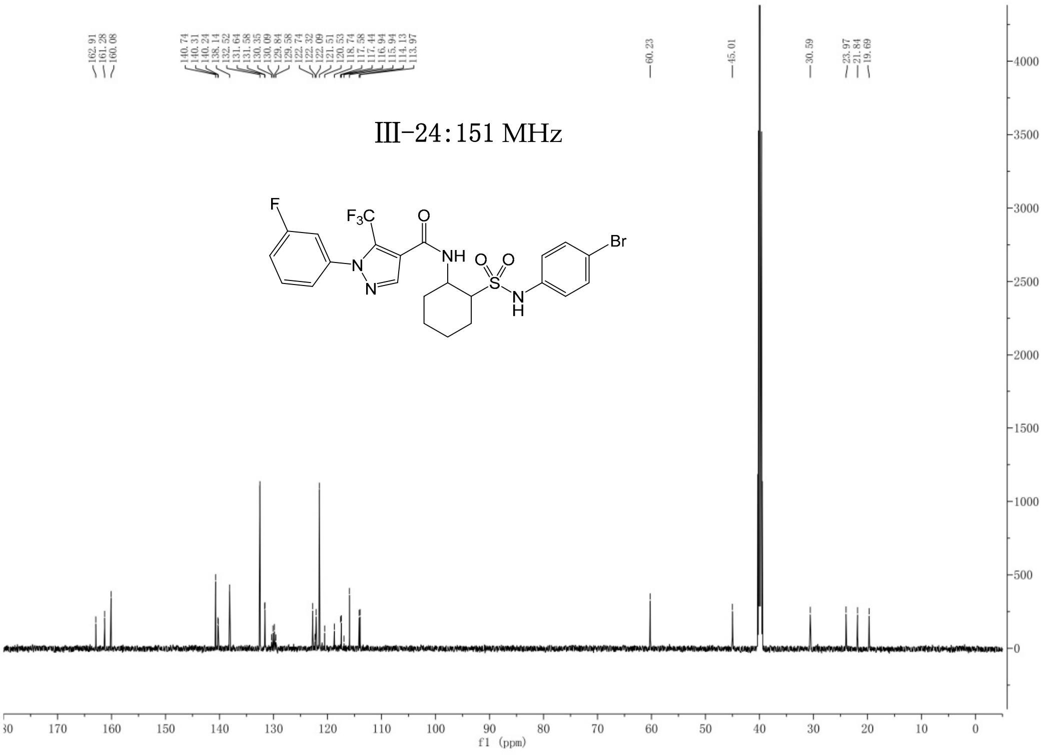
Ⅲ-24:151 MHz
162.91
161.28
160.08
140.74
140.31
140.24
138.14
132.52
131.64
131.58
130.35
130.09
129.84
129.58
122.74
122.32
122.09
121.51
120.53
118.74
117.58
117.44
116.94
115.94
114.13
113.97
60.23
45.01
30.59
23.97
21.84
19.69
f1 (ppm)

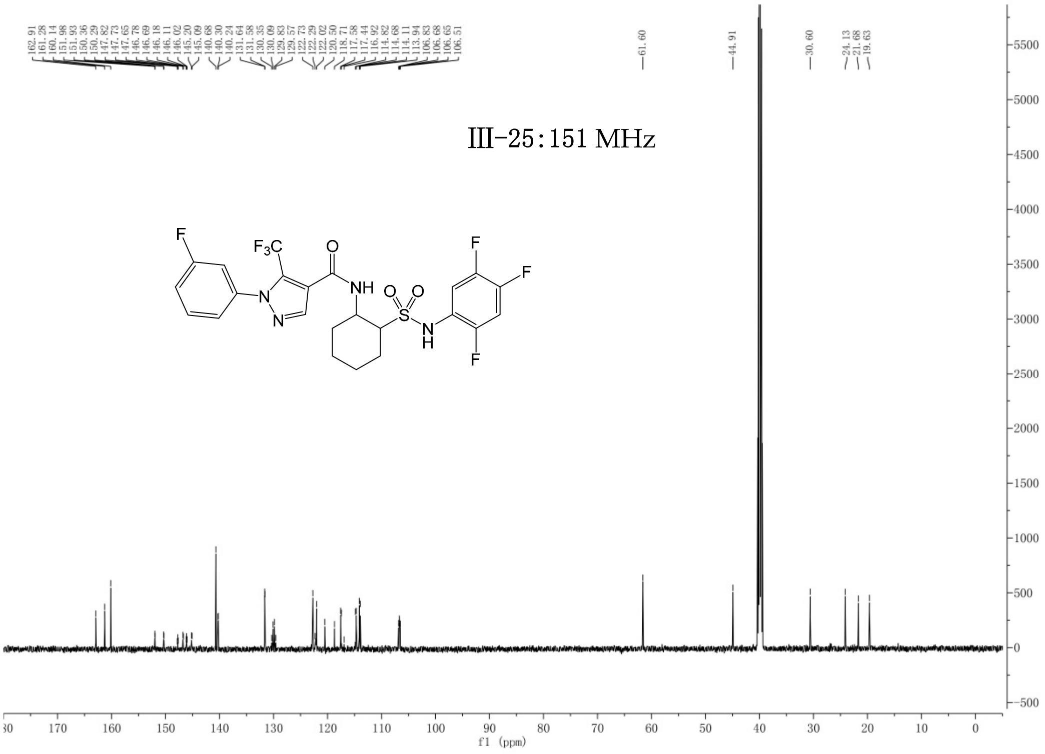
Ⅲ-25:151 MHz
61.60
44.91
30.60
24.13
21.68
19.63
f1 (ppm)

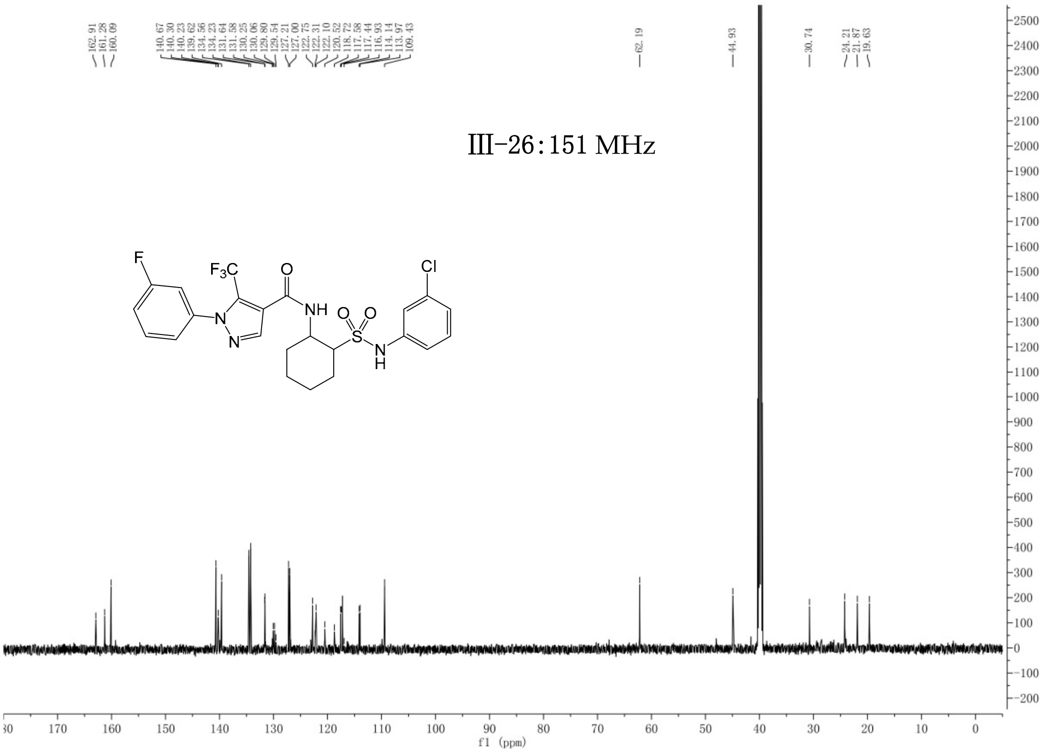
Ⅲ-26:151 MHz
162.91
161.28
160.09
62.19
44.93
30.74
24.21
21.87
19.63
f1 (ppm)

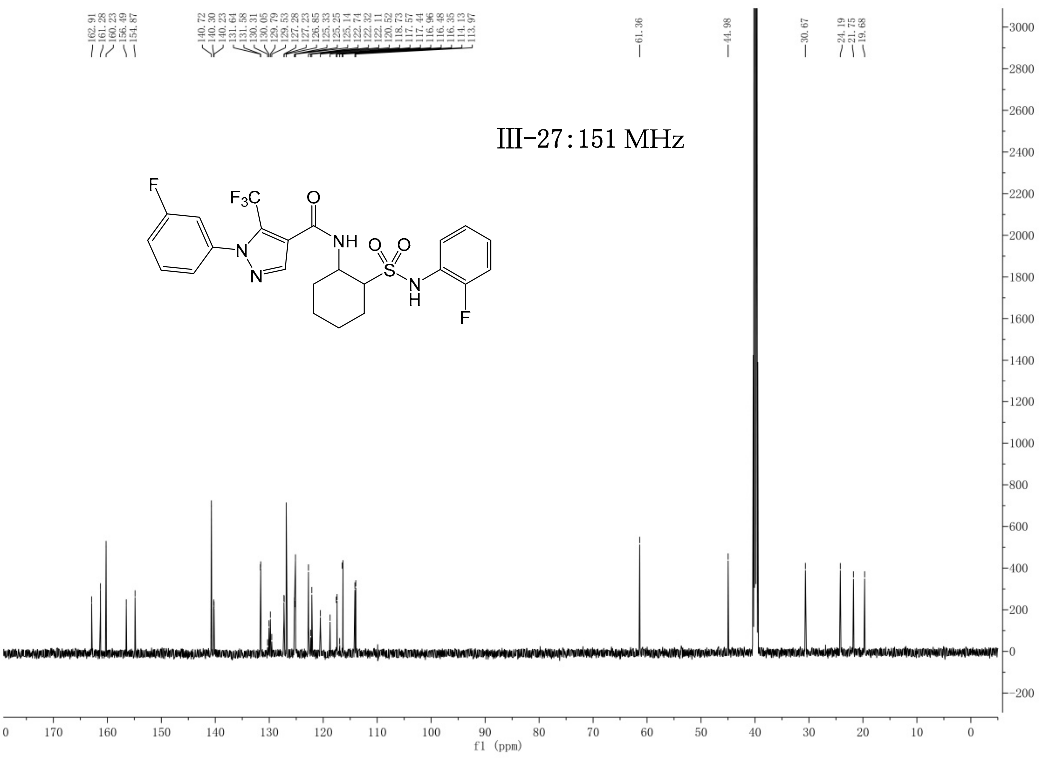
Ⅲ-27:151 MHz
f1 (ppm)

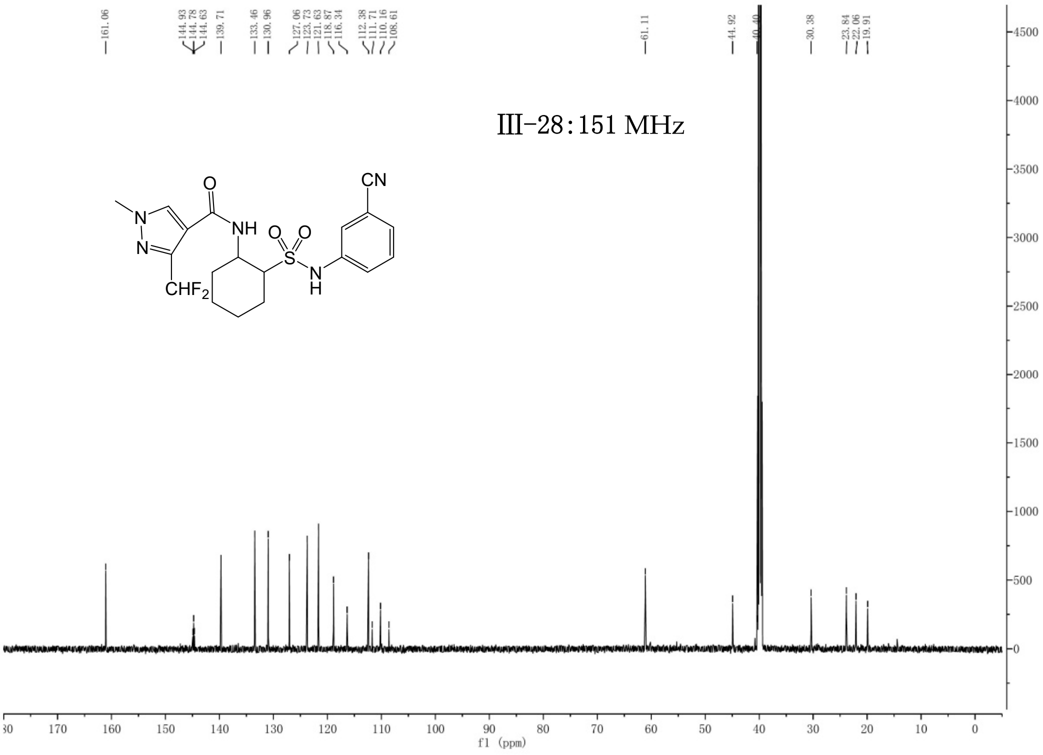
Ⅲ-28:151 MHz
f1 (ppm)

Ⅲ-29:151 MHz

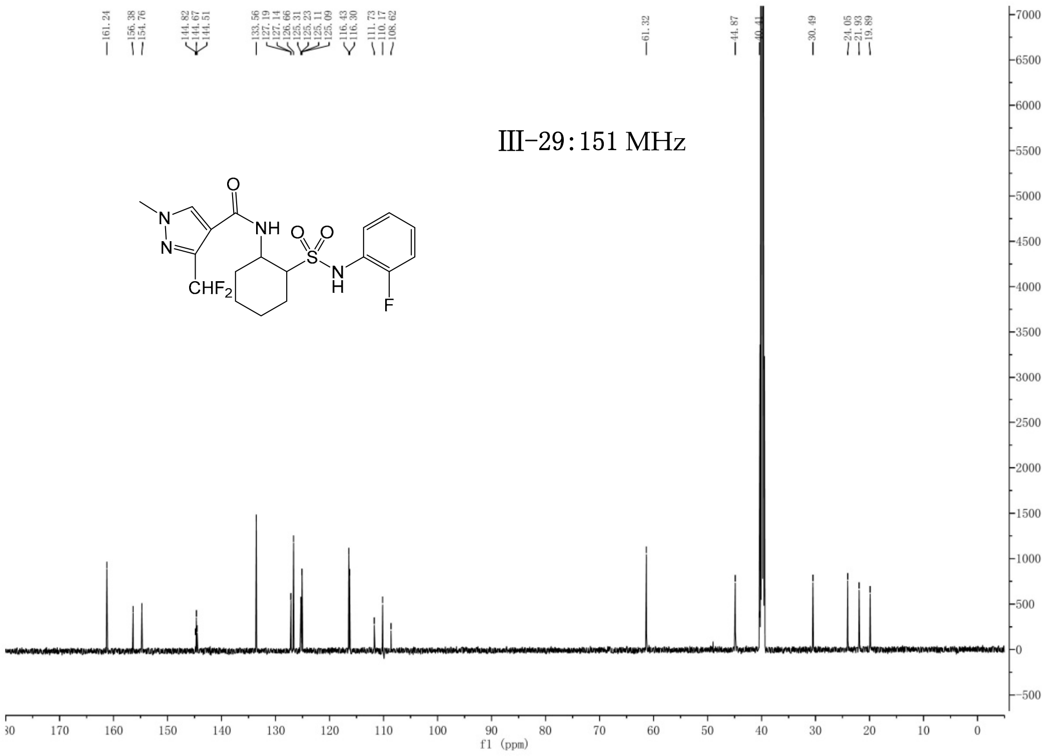

Ⅲ-30:151 MHz

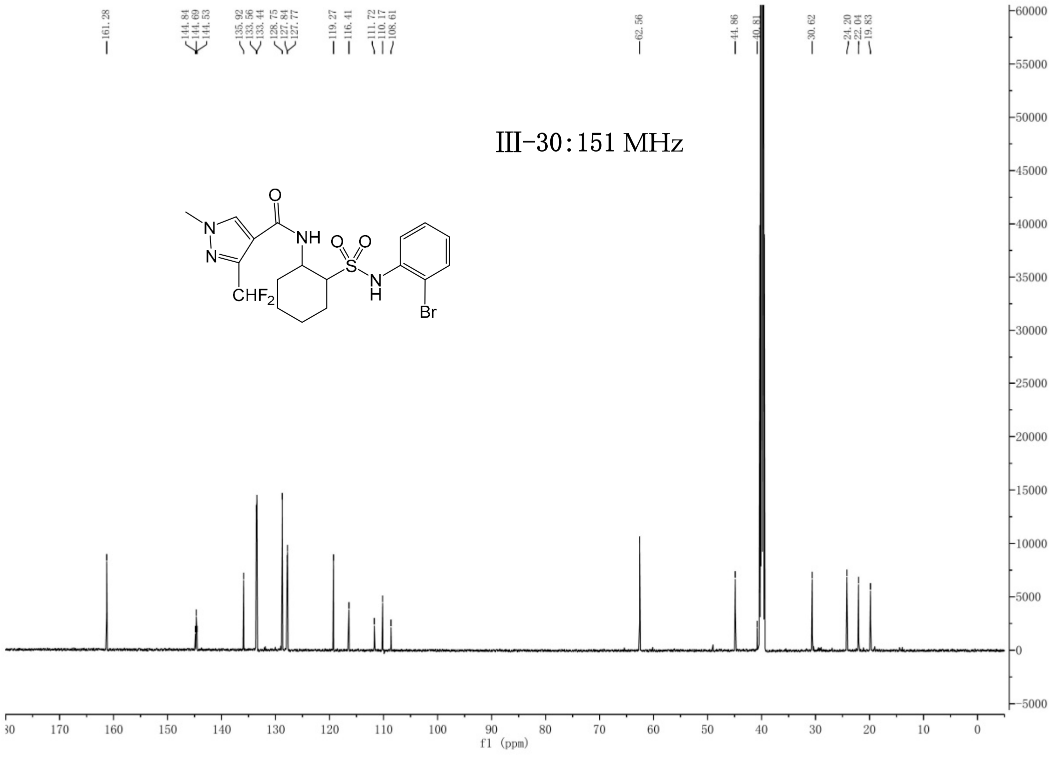

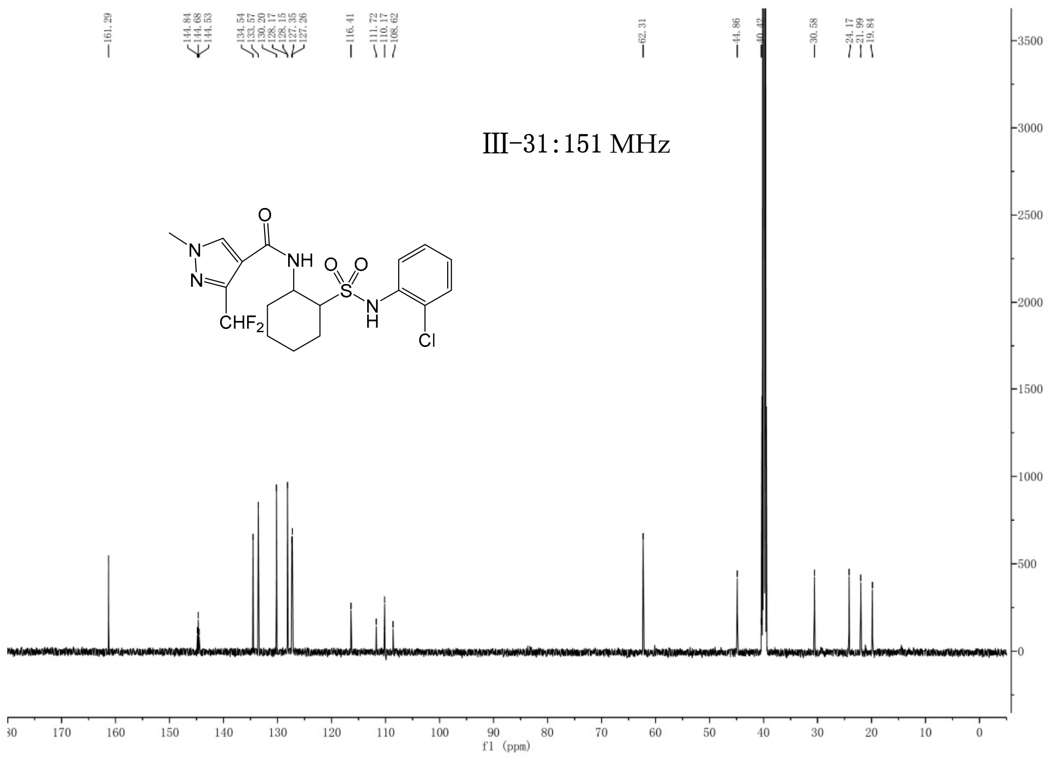
Ⅲ-31：151 MHz
f1 (ppm)

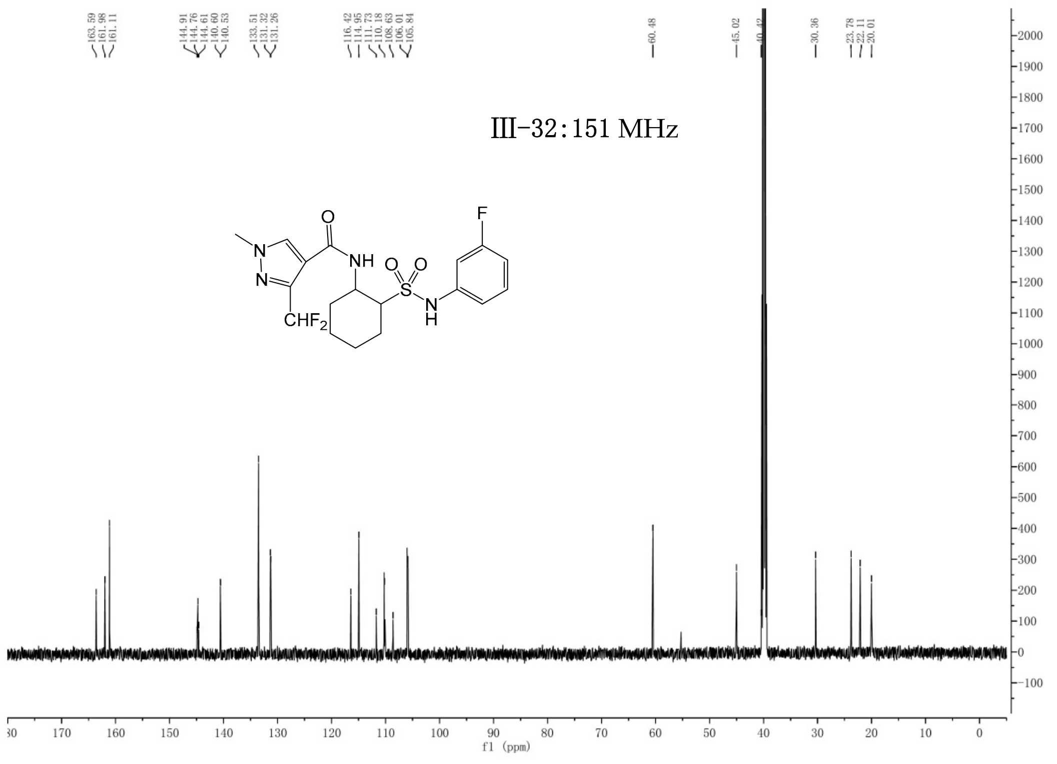
Ⅲ-32：151 MHz
f1 (ppm)

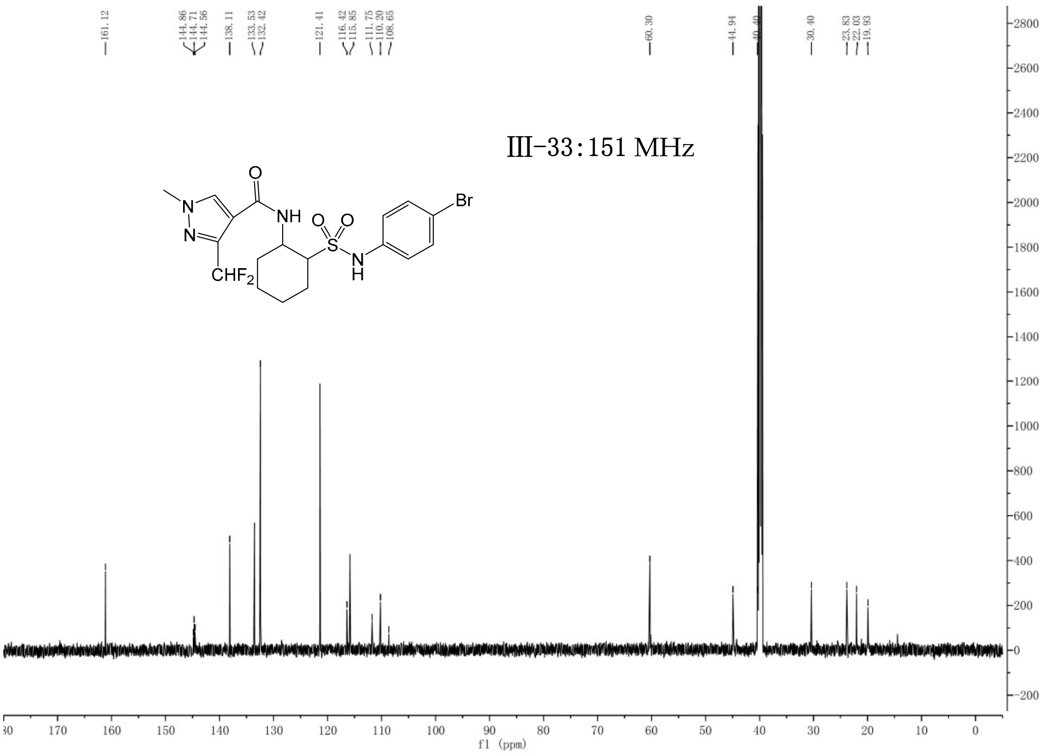
Ⅲ-33：151 MHz
O
NH
N
N
O
O
S
N
H
Br
CHF2
f1 (ppm)

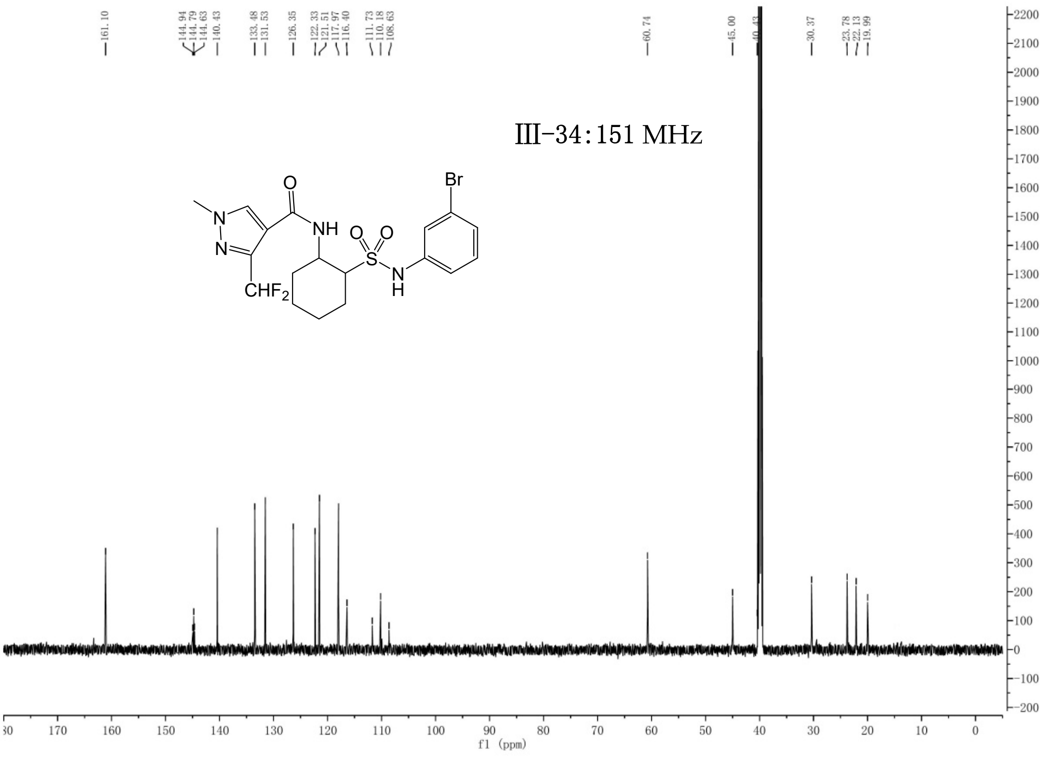
Ⅲ-34：151 MHz
O
NH
N
N
O
O
S
N
H
Br
CHF2
f1 (ppm)

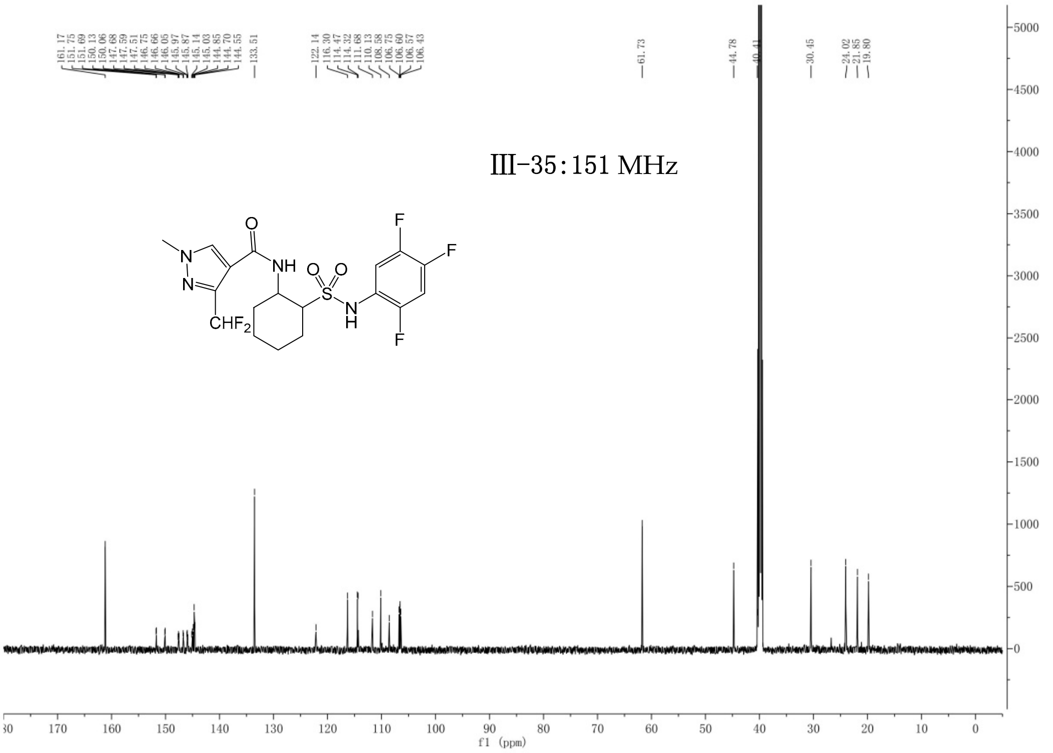
Ⅲ-35:151 MHz
CHF2
f1 (ppm)

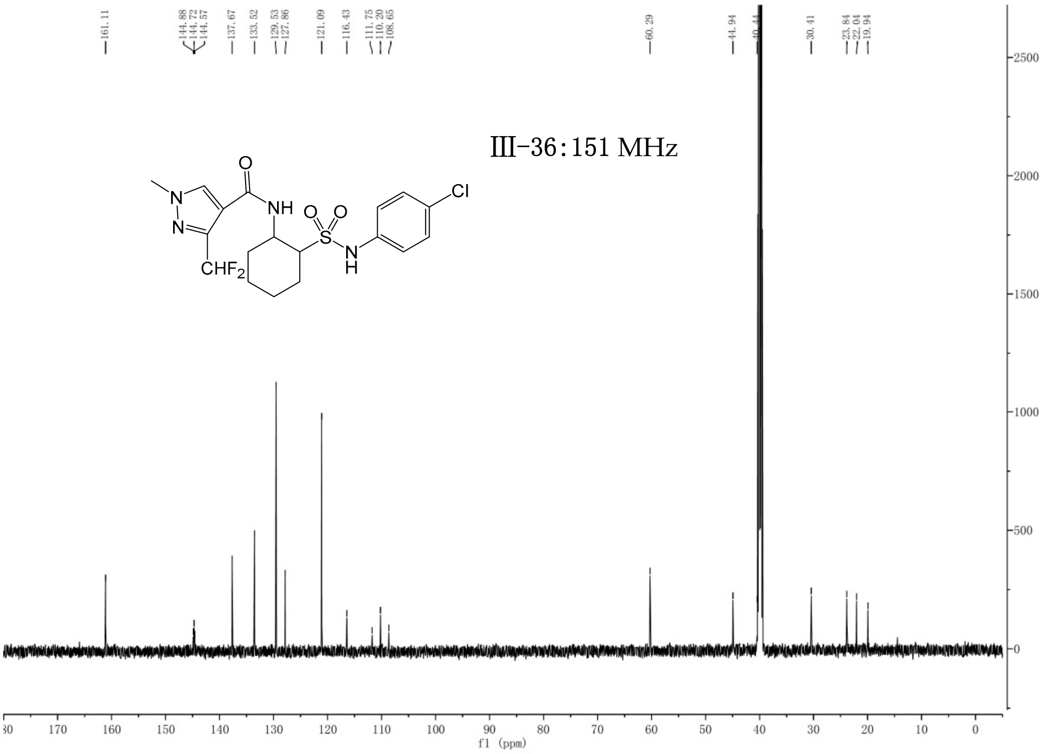
Ⅲ-36:151 MHz
CHF2
f1 (ppm)
